# Dual gold and photoredox catalysed C–H activation of arenes for aryl–aryl cross couplings†
†Electronic supplementary information (ESI) available: Experimental procedures, full optimisation tables, control reaction, mechanistic studies, characterisation data and copies of NMR spectra of new compounds. See DOI: 10.1039/c6sc05469b



**DOI:** 10.1039/c6sc05469b

**Published:** 2017-01-27

**Authors:** V. Gauchot, D. R. Sutherland, A.-L. Lee

**Affiliations:** a Institute of Chemical Sciences , Heriot-Watt University , Edinburgh EH14 4AS , Scotland , UK . Email: A.Lee@hw.ac.uk

## Abstract

A mild and fully catalytic aryl–aryl cross coupling *via* gold-catalysed C–H activation has been achieved by merging gold and photoredox catalysis. The procedure is free of stoichiometric oxidants and additives, which were previously required in gold-catalysed C–H activation reactions. Exploiting dual gold and photoredox catalysis confers regioselectivity *via* the crucial gold-catalysed C–H activation step, which is not present in the unselective photocatalysis-only counterpart.

## Abstract 2

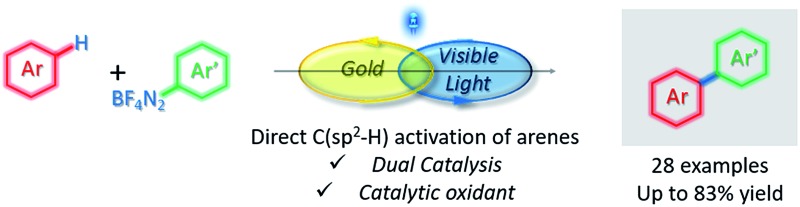
A mild and fully catalytic aryl–aryl cross coupling *via* gold-catalyzed C–H activation has been achieved by merging gold and photoredox catalysis.

## Introduction

**Scheme 1:**
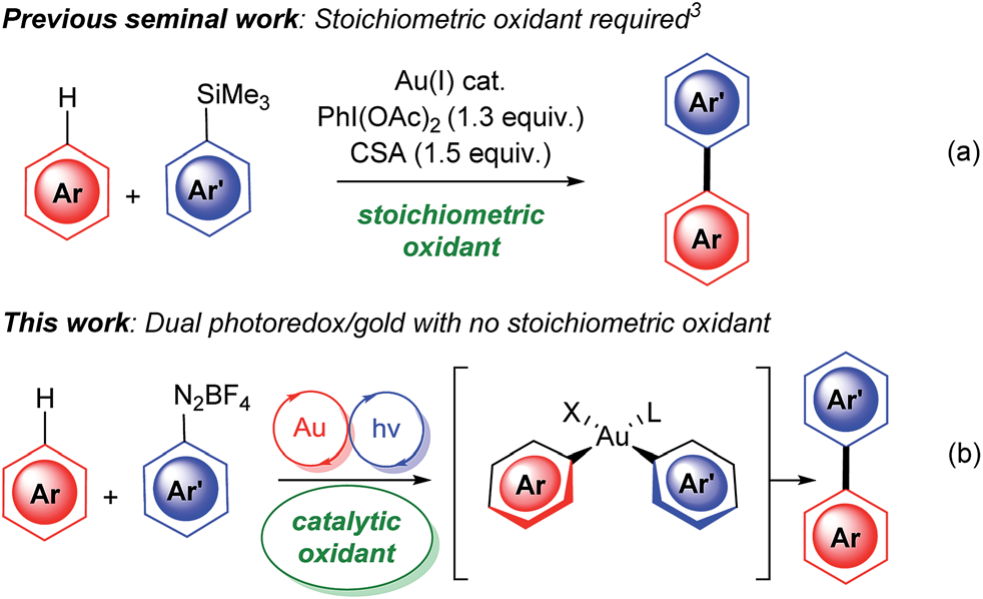
Gold-catalysed aryl–aryl couplings *via* C–H activation.

The increased drive to develop more sustainable methods for synthesis has led to a surge in research on C–H functionalisations.^1^ Within this context, direct aryl C–H functionalisations using gold catalysis^2^ is a relatively young and overlooked field compared to the more developed palladium, ruthenium and rhodium counterparts. Nevertheless, the mild conditions under which gold-catalysis can activate C–H bonds, as well as the regioselectivity observed in the absence of directing groups,^2^ provides many golden opportunities for this developing field. In the specific area of aryl–aryl cross-couplings *via* C–H activation, Lloyd-Jones and Russell elegantly showcased that gold catalysis can be used to site selectively arylate arylsilanes (Scheme 1a).^3^ More recently, Larrosa disclosed his seminal work on oxidative cross-couplings *via* double C–H activation to couple electron-poor with electron-rich arenes.^4^ Despite these advances, there remain several limitations, one of which is the often limited arene substrate scope.^2*a*^ The other major limitation is the requirement for a stoichiometric oxidant to access the Au(i)/Au(iii) cycle required for cross-couplings:^2,5^ the benefit of employing C–H activation to avoid arene prefunctionalisation is thus somewhat offset by the generation of stoichiometric organic waste from the oxidant, and the use of the latter can also limit functional group tolerance. There is therefore a clear need to develop couplings that do not require stoichiometric oxidants.^2*a*^ Within this context, we herein disclose the first dual gold and photoredox catalysed aryl–aryl cross coupling *via* C–H activation (Scheme 1b), which also constitutes the first gold-catalysed C(sp^2^)-H activation reaction which does not require stoichiometric oxidants.

The use of dual^6^ gold and photoredox catalysis^7^ to access Au(i)/Au(iii) catalytic cycles was recently pioneered by Glorius^8^ and Toste.^9–11^ Its use in cross-couplings has only very recently been reported: Sonogashira-type couplings^12^ and Suzuki-type couplings were revealed this year, the latter independently by our group^13^ and Fouquet.^14,15^ To the best of our knowledge, however, aryl–aryl couplings *via* C(sp^2^)-H activation using dual gold and photoredox catalysis has yet to be achieved, although it was recently attempted by Maestri and Malacria.^16^ Under their conditions, they instead discovered that the coupling between unactivated arenes and diazonium salts could occur under photocatalysis-only conditions (no gold) through mechanistically distinct formal homolytic aromatic substitutions, which does not involve C–H activation. However, poor regioselectivities (mixtures of *ortho*, *meta* and *para* coupling) were observed and 40 equivalents of arene were generally required for this radical reaction.^16^ Therefore, aryl–aryl couplings *via* C(sp^2^)-H activation involving *dual gold* and photoredox catalysis is clearly desirable, as it will not only prove for the first time that catalytic oxidants can be utilised in the general field of gold-catalysed C–H activations, but it should also significantly improve the regioselectivities and arene equivalents in the aryl–aryl couplings, compared to the mechanistically distinct photocatalysis-only reaction.

## Results and discussion

Since electrophilic Au(iii) is known to C–H activate electron rich arenes,^3g,17–19^ and using insights gained from our previous studies,^13^ we surmised that a combination of an aryldiazonium salt^20^ with PPh_3_AuNTf_2_ and a photoredox catalyst should furnish an electrophilic aryl Au(iii) species (**III**, Scheme 3) capable of C–H activating a suitable arene in order to form our cross-coupled product (see later for mechanism). We thus initiated our studies using mesitylene **1a** as the arene with Ru(bpy)_3_(PF_6_)_2_ as the photoredox catalyst (Table 1). To our delight, the coupling product **3aa** was observed in a promising 31% yield (Entry 1).

**Table 1:** Selected optimisation reactions

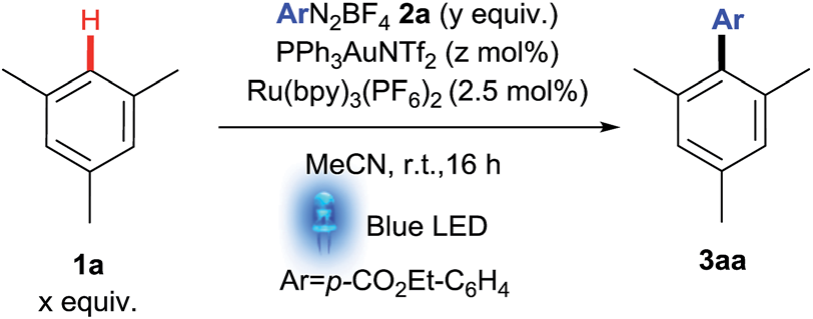					
Entry^*a*^	*x*	*y*	*z*	Modification	Yield^*b*^ (%)
1	1	2	5	—	31
2	1	1	5	—	51
**3**	**3**	**1**	**5**	**—**	**67**
4	10	1	5	—	54
5	3	1	5	Eosin Y instead of [Ru]	62
6	3	1	5	Fluorescein instead of [Ru]	58
**7**	**3**	**1**	**10**	**—**	**81**

^*a*^Degassed MeCN.

^*b*^Determined by ^1^H NMR analysis using dimethylsulfone as internal standard.

**Scheme 2:**
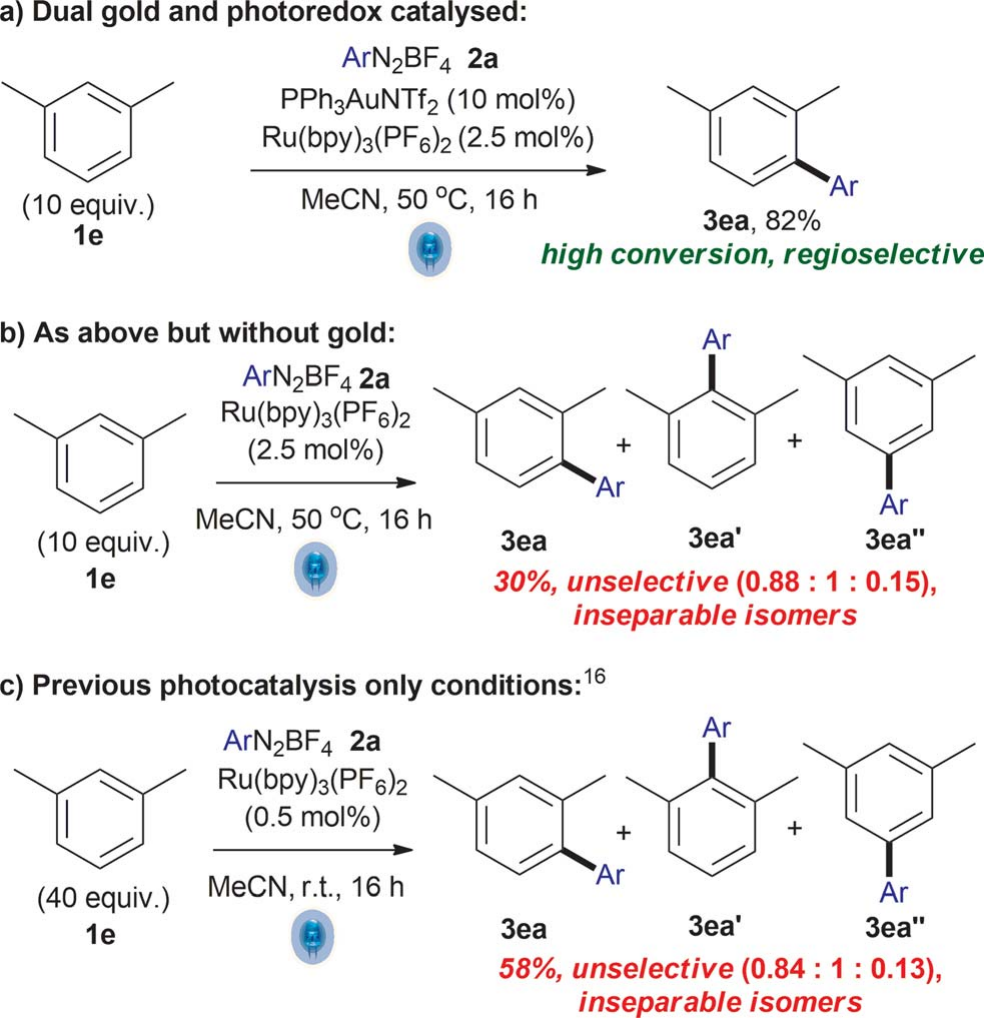
Dual gold and photoredox catalysis confers regioselectivity.

Crucially, control experiments in the absence of gold catalyst,^21^ Ru catalyst or light resulted in little or no conversion (see ESI†), confirming that it is a *dual* gold/photoredox coupling reaction under these conditions (see Scheme 2 for further confirmation). Optimisation studies showed that a small excess of arene **1a** is beneficial (Entry 3) but a large excess hampers the reaction in this case (Entry 4). Employment of organic dyes^22^ eosin Y and fluorescein instead of Ru(bpy)_3_(PF_6_)_2_ proved to be a potentially greener alternative (Entries 5–6), although we opted to continue our studies using the better performing Ru catalyst. Finally, a good **3aa** yield of 81% was achieved by increasing the gold catalyst loading (Entry 7).^23^

With these optimised conditions in hand, an aryldiazonium substrate scope was carried out (Table 2). Ester-(**3aa**) and amide-substituted (**3ak**) substrates, as well as halogenated substrates **3ab–3ae** react smoothly (50–80%), as do *meta*- and *para*-substituted nitro substrates (**3af–3ag**). The *ortho*-substituted **3ah**, however, is furnished in a modest 37% yield, presumably due to steric effects. Yields of **3ai** and **3aj** were moderate under standard conditions, but the yield of **3ai** was successfully improved to 60% under more forcing conditions (10 equiv. **1a** and 50 °C). Predictably,^8a,12b,13^ electron-rich aryldiazoniums react more sluggishly, with decreasing yields observed with more electron rich aryls (**3am–3an** 48%, while **3ao** < 26%).

**Table 2:** Aryldiazonium scope

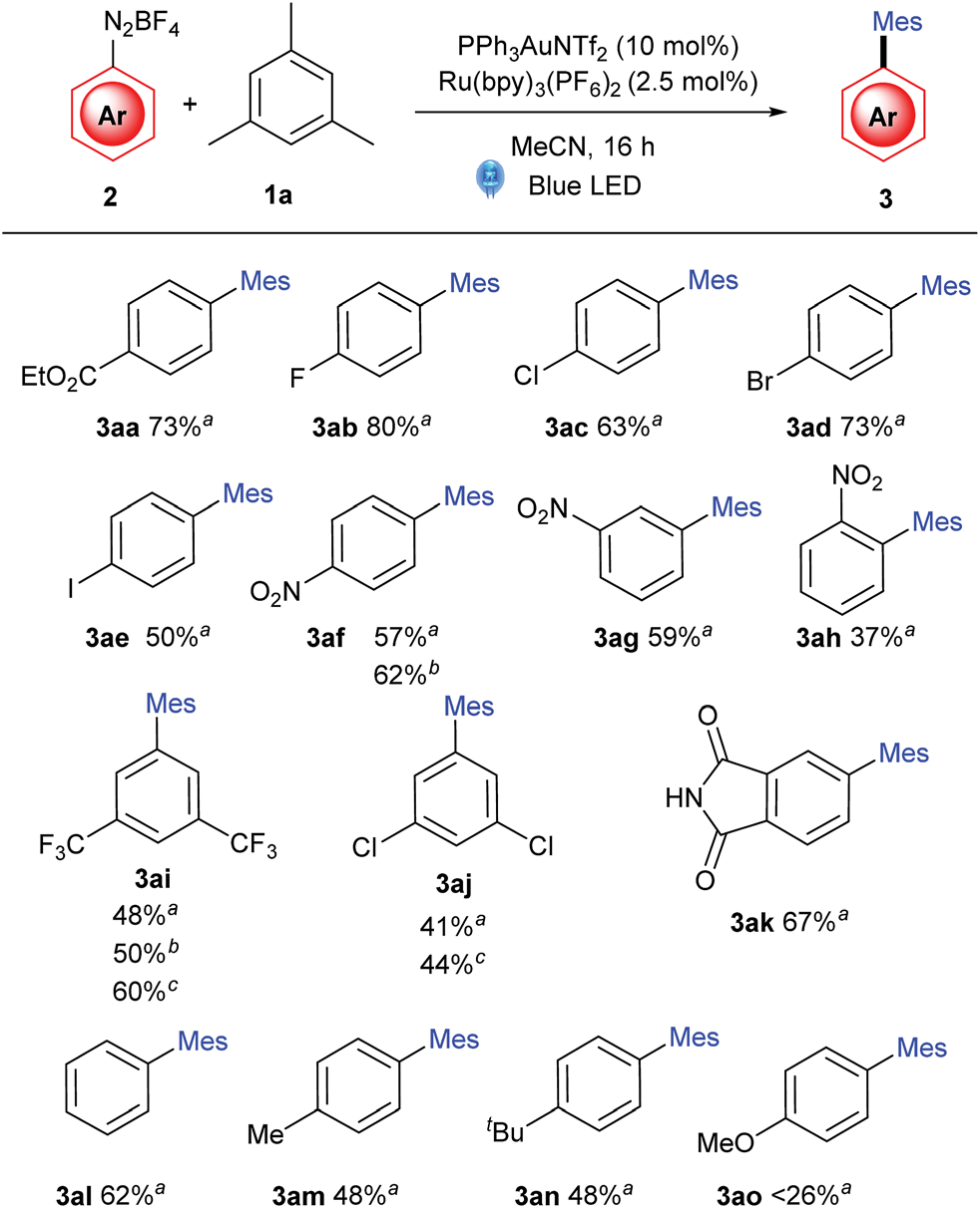

^*a*^Method A: **2** (0.1 mmol), arene (3 equiv.) [Ru] and [Au] were dissolved in degassed MeCN, and stirred at rt under blue LED irradiation.

^*b*^Method B: 3 equiv. of **1a**, 50 °C.

^*c*^Method C: 10 equiv. **1a**, 50 °C. Isolated yields reported.

As for the arene scope, Au(iii)-mediated C–H activation is known to proceed *via* electrophilic aromatic substitution onto Au(iii) (see later), thereby rendering electron-poor arenes unsuitable candidates for these conditions. With this in mind, suitable electron neutral and electron rich arenes were evaluated as shown in Table 3. While steric hindrance in the form of double *ortho* substitution is tolerated in mesitylene **3aa** (73%), the yield begins to drop off with increasingly hindered *ortho*-substituents (**3ba**, **3ca**). *Para*- and *meta*-xylene also couples with high yields (82%), as does toluene (**3da**) and ^*t*^butylbenzene (**3ea**). Predictably, **3da** and **3ea** are formed as *o*-/*p*-isomers, although the major *para*-**3da** can be isolated in a good 56% yield. The *p*-/*o*- ratio is a good 5.7 : 1 for the more hindered **3ga**.

**Table 3:** Arene scope

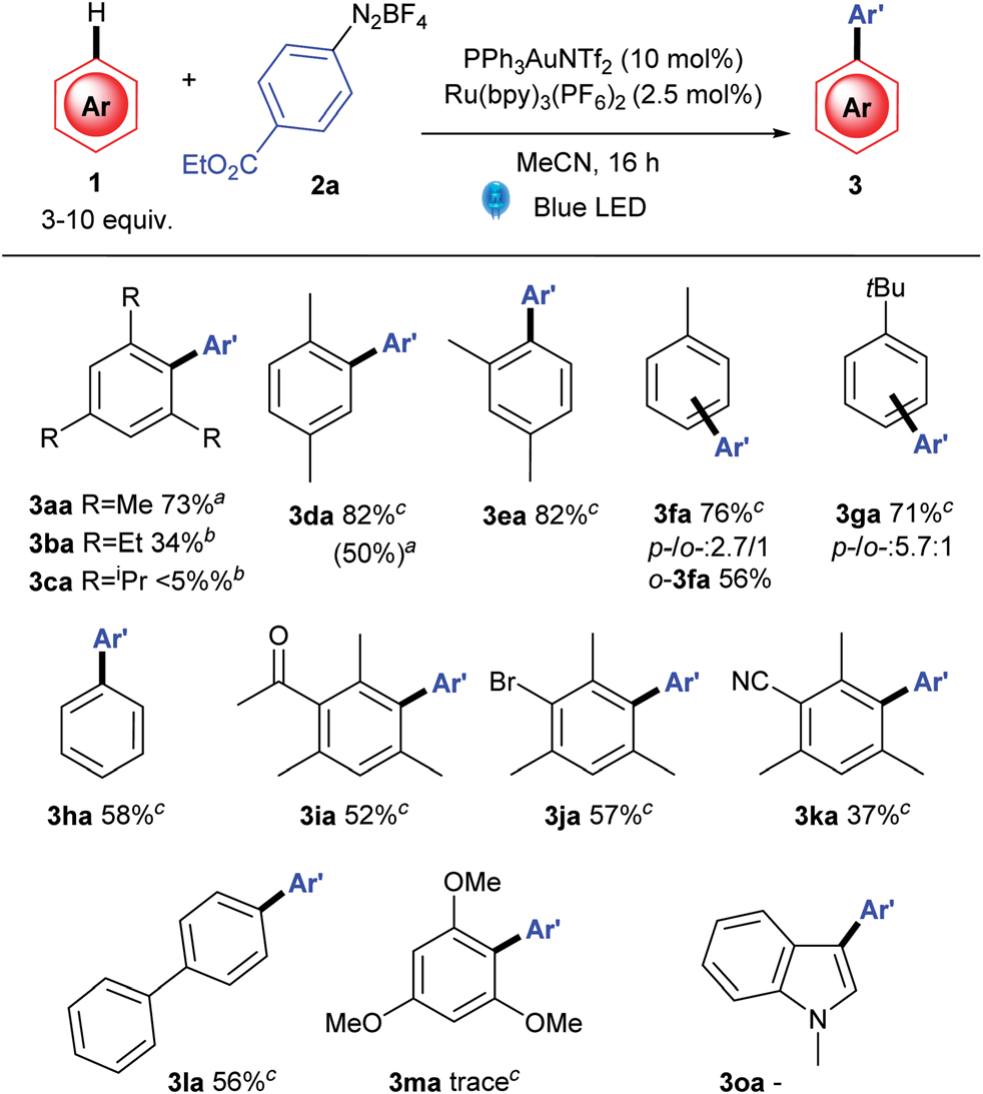

^*a*^Method A: **2** (0.1 mmol), arene (3 equiv.) [Ru] and [Au] were dissolved in degassed MeCN, and stirred at rt under blue LED irradiation.

^*b*^Method B: 3 equiv. of **1a**, 50 °C.

^*c*^Method C: 10 equiv. **1a**, 50 °C. Isolated yields reported.

This is in stark contrast to the photocatalysis-only reaction (Scheme 2). In the absence of gold, yield (30%) and selectivity (0.88 : 1 : 0.15 of **3ea** : **3ea′** : **3ea′′**) are both very poor (Scheme 2b) compared to the fully selective dual catalytic reaction (Scheme 2a). Adopting the literature photocatalysis-only conditions^16^ also result in a similarly unselective reaction, although the conversion is improved (58% combined yield of inseparable isomers, Scheme 2c). These controls show the significant benefit of utilising the regioselectivity conferred by the gold C–H activation step in the dual gold and photoredox reaction (Scheme 2a) and is further proof that the reaction described here is *not* a photocatalysis-only reaction.

Next, mesitylenes bearing electron-withdrawing substituents successfully couple (Table 3, **3ia–3ja**, 52–57%), although the yield drops to 37% in the presence of the more withdrawing CN group (**3ka**). Biphenyl pleasingly reacts exclusively at the *para*-position to yield triaryl **3la** in 56% yield. Finally, very electron rich arenes and heteroarenes such as 1,3,5-trimethoxybenzene and *N*-methyl indole do not currently cross-couple well under these conditions, due to competing azo coupling (see ESI†).^24,25^

**Scheme 3:**
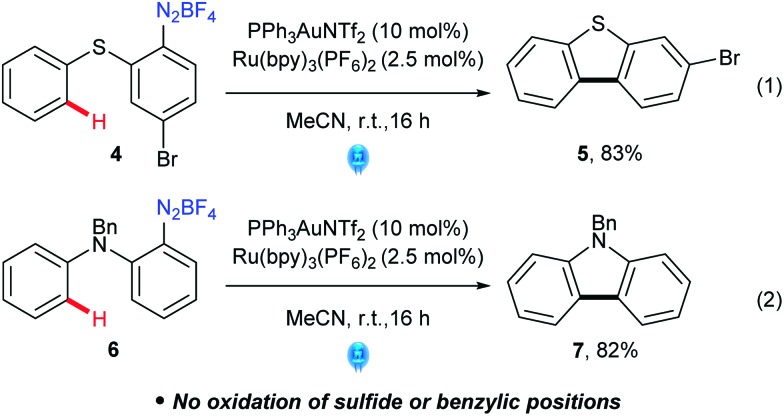
Intramolecular aryl–aryl coupling *via* C–H activation.

Pleasingly, however, more electron rich arenes are viable arenes for intramolecular C–H couplings, as exemplified by the formation of **5** in 83% yield (Scheme 3). While these cyclisations have been attempted under photocatalysis-only conditions, reported yields were very low (0–25%) due to competing deazotisation.^26^ Carbazole^27^ **7** can also be accessed from **6** in 82% yield, which is of note as traditional Pschorr cyclisations^28^ do not typically work well for carbazoles. Indeed, **5** and **7** are only formed in 36% and 41% (NMR yields) respectively in the absence of gold. Moreover, the readily oxidisable sulfide (**4**) and benzyl (**6**) are tolerated under these conditions, showcasing the potential of dual catalysis to significantly improve the C–H activation cross coupling under mild conditions compared to previously required stoichiometric oxidant conditions.

**Scheme 4:**
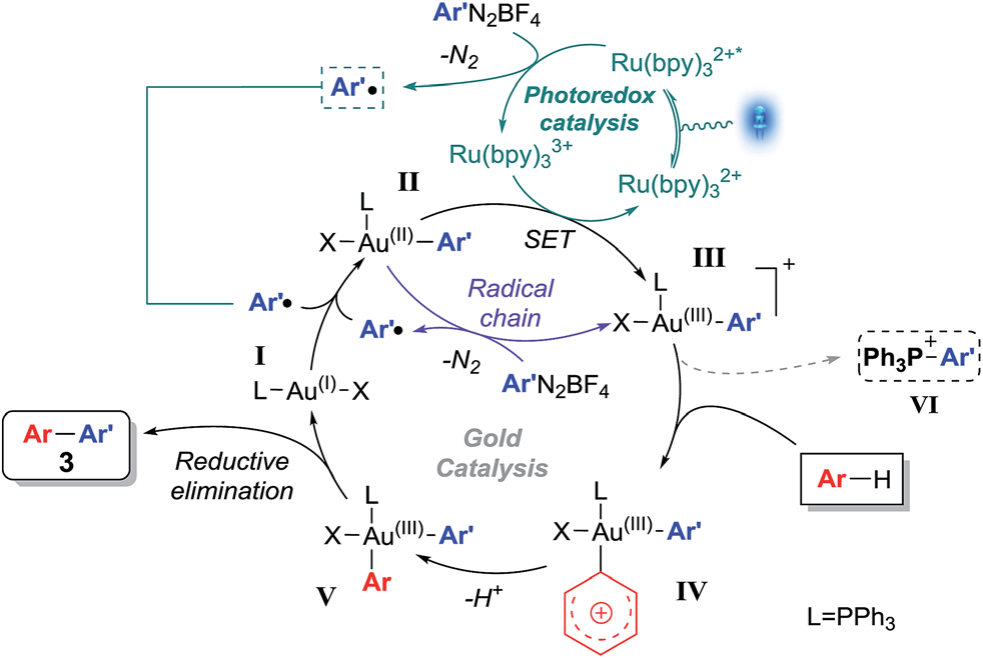
Plausible mechanism.

Based on a combination of various literature reports,^8a,9a,12b,13,29^ a plausible mechanism for the cross-coupling is shown in Scheme 4. Initial oxidation of the Au(i) catalyst **I** *via* addition of an aryl radical (**I** → **II**), is followed by a subsequent SET to form Au(iii) intermediate **III**,^30^ regenerating the photocatalyst. Alternatively, quantum yield calculations carried out on related dual triphenylphosphine gold/visible-light catalysed systems revealed that species **II** can also undergo SET with another equivalent of diazonium salt, to simultaneously yield the Au(iii) species **III** along with an aryl radical.^12*b*^ The arene partner then undergoes electrophilic auration with the Lewis acidic species **III** to give intermediate **IV**, which explains the regioselectivities observed.^3g,17–19^ The corresponding intermediate **V** then reductively eliminates to form the cross-coupled product **3**, while regenerating Au(i) catalyst **I**.

**Fig. 1:**
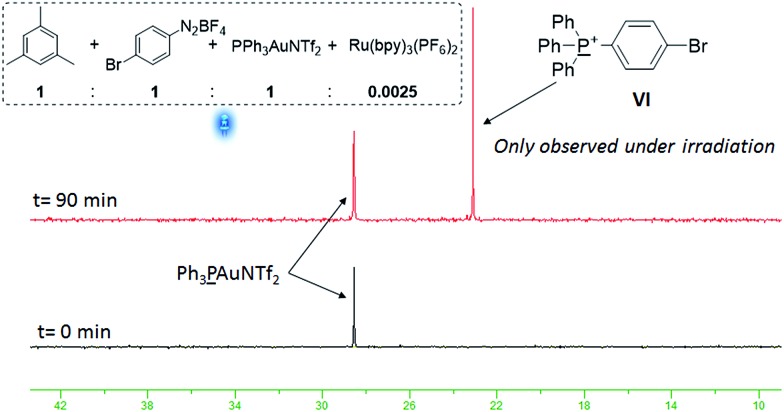
^31^P NMR studies in CD_3_CN.

In order to lend support to this mechanism, two reactions were set up using equimolar amounts of PPh_3_AuNTf_2_, **1a** and **2d**, and 2.5 mol% of Ru(bpy)_3_(PF_6_)_2_ in the presence and absence of light respectively. ^31^P NMR monitoring reveals that a new signal at *δ* 23.1 ppm appears for the irradiated reaction (Fig. 1), but is absent from the dark reaction. The transient species **III** is highly unstable^31^ and cannot be isolated, however, the signal at *δ* 23.1 ppm corresponds to species **VI** ^32^ which is formed by reductive elimination of **III**.^9*a*^ The detection of **VI** therefore implies that **III** is present in the reaction.^9a,13^

Furthermore, control experiments using various gold(i) species fail to form the coupling product **3** (see ESI†), lending further support to the hypothesis that intermediate **III** is the key species in the crucial electrophilic auration step. In addition, control experiments shown in Scheme 2 confirm that the mechanism is distinct from the formal homolytic aromatic substitutions observed in the photocatalysis-only reactions, since the regioselectivity observed supports the electrophilic auration step shown in Scheme 4 rather than the former, which is unselective. Additionally, the involvement of an aryl cation intermediate from the aryldiazonium salt **2** ^33^ can also be discounted by the fact that electron withdrawing aryldiazoniums react more readily than their electron rich counterparts (Table 2).

## Conclusions

In conclusion, we have developed the first dual gold/photoredox method for aryl–aryl cross coupling *via* direct C–H activation of arenes under mild conditions. The use of dual catalysis has allowed us to address and overcome a major limitation encountered with gold-catalysed C–H activations: the requirement for stoichiometric oxidants and its corresponding waste. As is the case with current gold-catalysed C–H activation reactions,^2^ the arene substrate scope for the intermolecular coupling still has its limitations (although the intramolecular version shows great promise) and addressing this issue remains a future challenge for the field. Nevertheless, we envisage that the development of the first fully catalytic system constitutes significant progress for the field of gold-catalysed C–H activation and functionalisation of arenes. In addition, control experiments show that exploiting *dual gold* and photoredox catalysis confers regioselectivity *via* the crucial gold-catalysed C–H activation step, which is not present in the unselective photocatalysis-only counterpart.
