# Supplementary material for: Dual gold and photoredox catalysed C–H activation of arenes for aryl–aryl cross couplings
Source: Chem Sci. 2017 Jan 27;8(4):2885–9. doi: 10.1039/c6sc05469b (PMC5427993; doi:10.1039/c6sc05469b)
Supplement: Supplementary file 1 [file SC-008-C6SC05469B-s001.pdf]

Electronic Supplementary Material (ESI) for Chemical Science.
This journal is © The Royal Society of Chemistry 2017

Dual Gold and Photoredox Catalysed C-H Activation of Arenes for Aryl-Aryl Cross Couplings

Vincent Gauchot, Daniel R. Sutherland and Ai-Lan Lee*

SUPPORTING INFORMATION

Contents

1 - General experimental section 2

2 - Optimisation of the reaction conditions 3

3 - Mechanistic experiments 7

4 - Photocatalysis-only control experiments 10

5 - Preparation of the substrates 14

6 - General procedure for the dual-catalysed C-H functionalization of electron-rich arenes 18

7 - Relevant NMR spectra 27

8 - References 61

1 - General Experimental Section

Unless otherwise stated, all reagents were obtained commercially and used without further purification. Unless otherwise stated, all dual gold/photoredox reactions were carried out under inert atmosphere using Schlenk techniques. Prior to each catalytic run, the reaction vessel was wrapped in aluminium foil and the mixture was degassed in the dark through three freeze-thaw (1 min)-pump cycles. The foil was then removed, and light irradiation was performed using blue LEDs (1.5 Watt/foot). The light source was placed *ca* 10 cm away from the reaction vessel, to prevent excess heating. TLC analysis was performed on Merck 60 F254 Silica aluminium sheets, and visualised by UV (254 nm) and/or stained by the use of aqueous acidic $KMnO_4$. Dry solvents were obtained from a solvent purification system, and solvents used for purification by chromatography were obtained from Fisher Scientific.

^{1}H, ^{13}C and ^{31}P NMR spectra were recorded at ambient temperatures on Bruker AV-300 and AV-400 MHz spectrometers, and chemical shifts (δ in ppm) were referenced to residual solvent peaks. *J* values are given in Hz and s, d, dd, ddd, dt, t, td, tt, q, qn, sext and m abbreviations correspond to singlet, broad singlet, doublet, doublet of doublet, doublet of doublet of doublets, doublet of triplets, triplet, triplet of doublets, triplet of triplets, quartet, quintet, sextet and multiplet. Mass Spectra were obtained at the EPSRC National Mass Spectrometry Facility in Swansea, and spectra were recorded on a Thermo Scientific LTQ Orbitrap XL spectrometer.

2-Optimisation of the reactions conditions

2.1 – Solvent Screen

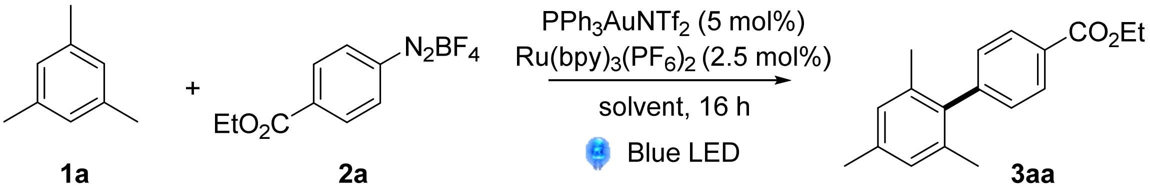

1a **2a** **3aa**

Entry	Solvent	NMR Yield[a]
**1**	CH_2Cl_2	7%
**2**	THF	no product detected
**3**	DMF	no product detected
**4**	CH_3CN	<50% isolated
**5**	Toluene	no product detected
**6**	DCE	8%

Reaction conditions: a solution of mesitylene **1a** (0.1 mmol), PPh_3AuNTf_2 (5 mol%), $Ru(bpy)_3(PF_6)_2$ (2.5 mol%) and aryldiazonium salt **2a** (0.2 mmol) in degassed solvent was stirred under light irradiation using blue LEDs for 16 h. [a]Dimethylsulfone was used as the internal standard

2.2 – Mesitylene equivalents screen

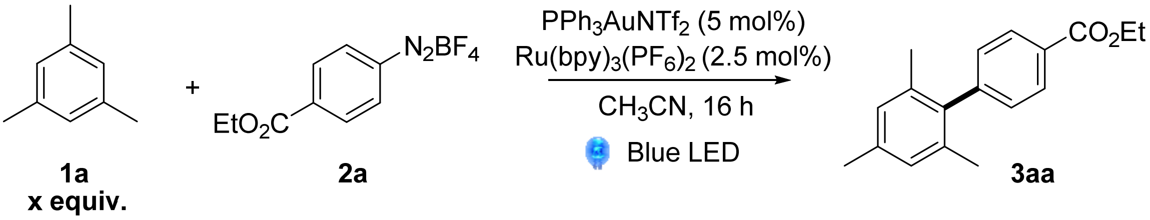

Entry	Mesitylene equivalents	NMR yield[a]
**1**	0.5 (original conditions)	31%
**2**	1	51%
**3**	2	61%
**4**	3	67%
**5**	4	68%
**6**	5	52%
**5**	10	54%

Reaction carried out on a 0.1 mmol scale (1 M in MeCN). [a]Dimethylsulfone was used as the internal standard.

2.3 – Impact of the catalysts loading

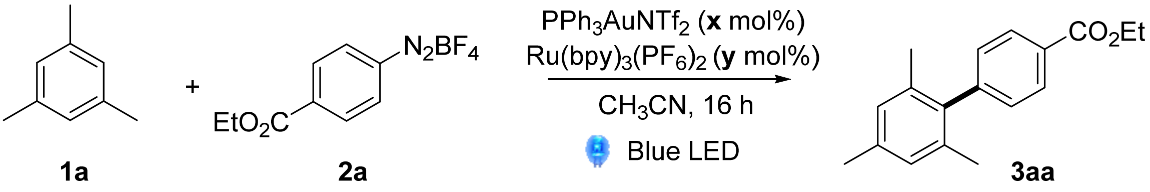

Entry	[Au] loading	[Ru] loading	NMR yield[a]
**1**	1 mol%	2.5 mol%	49%
**2**	5 mol%	2.5 mol%	67%
**3**	5 mol%	1 mol%	63%
**4**	5 mol%	5 mol%	43%
**5**	10 mol%	2.5 mol%	81%

Reaction carried out on a 0.1 mmol scale (1 M in MeCN). [a]Dimethylsulfone was used as the internal standard.

2.4 - Nature of the photocatalyst

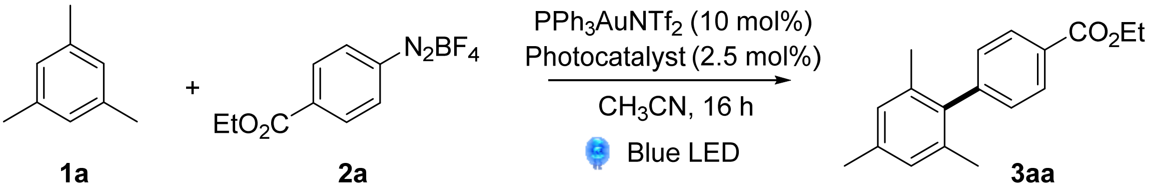

Entry	Photocatalyst	NMR yield[a]
**1**	$Ru(bpy)_3(PF_6)_2$	67%
**2**	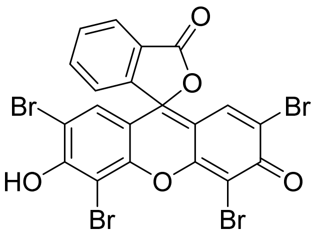 Eosin Y	62%
**3**	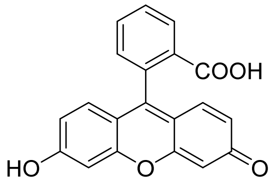 Fluorescein	58%

Reaction conditions: a solution of mesitylene **1a** (0.3 mmol), PPh_3AuNTf_2 (5 mol%), photocatalyst (2.5 mol%) and aryldiazonium salt **2a** (0.1 mmol) in degassed acetonitrile was stirred under light irradiation using blue LEDs for 16 h. [a]Dimethylsulfone was used as the internal standard.

***2.5 - Control reactions** (see also Section 4, page S-10)*

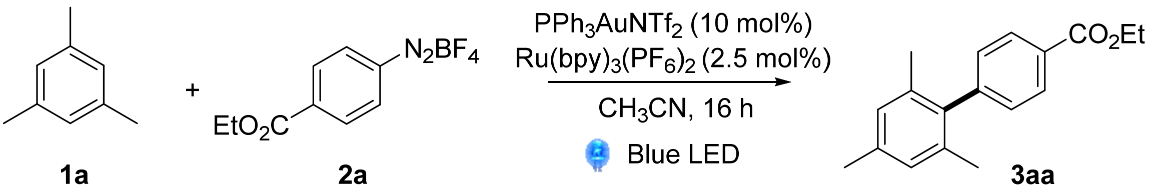

Entry	Parameter changed	NMR yield[c]
1[a]	No [Au] catalyst	<13%
2[a]	No[Ru] catalyst	no product detected
3[a]	No light	<9%
4[b]	Open flask, non-degassed solvent	51%

[a]Reaction conditions: a solution of mesitylene **1a** (0.1 mmol), PPh_3AuNTf_2 (5 mol%), $Ru(bpy)_3(PF_6)_2$ (2.5 mol%) and aryldiazonium salt **2a** (0.2 mmol) in degassed acetonitrile was stirred under light irradiation using blue LEDs for 16 h. [b]Reaction conditions: a solution of mesitylene **1a** (0.3 mmol), PPh_3AuNTf_2 (5 mol%), $Ru(bpy)_3(PF_6)_2$ (2.5 mol%) and aryldiazonium salt **2a** (0.1 mmol) in degassed acetonitrile was stirred under light irradiation using blue LEDs for 16 h. [c]Dimethylsulfone was used as the internal standard.

2.6 – Further optimisation with xylene

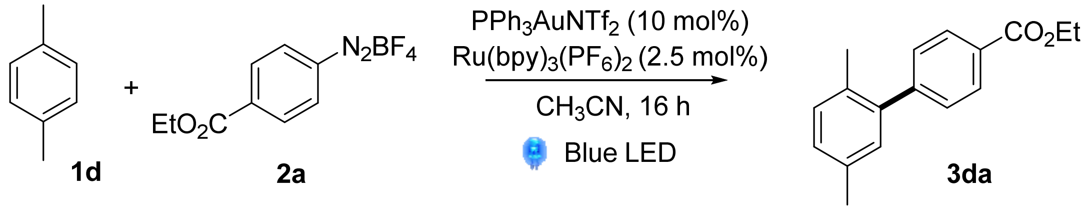

Entry	Conditions	Yield
1	r.t., 3 eq. arene	50%
2	r.t., 6 eq. arene	67%
3	r.t., 10 eq. arene	77%
**4**	**50 °C, 10 eq. arene**	**82%**
5	r.t., 10 eq arene, household light bulb	70%

3-Mechanistic experiments

For synthetic reasons, a *p*-bromosubstituted diazonium salt (**2d**) was selected as the coupling partner for these control experiments

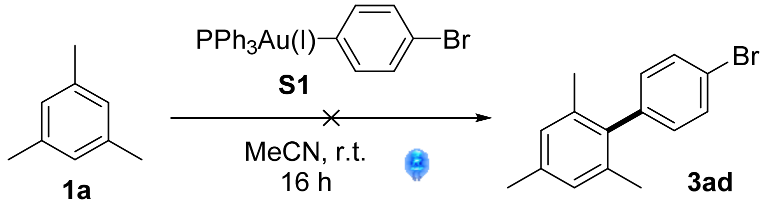

The gold complex **S1** was prepared according to literature.[1] In a Schlenk tube, the gold complex **S1** (55.4 mg, 0.09 mmol) and $Ru(bpy)_2(PF_6)_2$ (1.9 mg, 2.5 mol%) were dissolved in dry acetonitrile. The tube was wrapped in foil, and the mixture was degassed three times using the freeze-pump-thaw technique. Mesitylene **1a** (41.7 µl 3 equiv.) was added, and the mixture was stirred onvernight a room temperature under light irradiation using blue LEDs. The mixture was diluted in EtOAc (10 ml) and washed once with H_2O. The aqueous phase was re-extracted once with EtOAc, and the combined organic phases were washed with brine. The solution was dried over magnesium sulfate, filtered and the solvent was removed under vacuum. The residue was analysed by ^{1}H NMR.

No coupling product **3ad** could be observed after 16 h under these conditions, implying that **S1** is not an intermediate in the cross-coupling mechanistic cycle.

All NMR-monitored reactions were carried out in 3 ml vials on a 0.01 mmol scale, in 1 ml of deuterated acetonitrile. At an indicated time, an aliquot was taken out from the reaction vial and directly transferred into an NMR tube. ^{1}H and ^{31}P NMR experiments were recorded on a Bruker AV 400 MHz spectrometer. Upon completion of the analyses, the sample was immediately transferred back into the reaction vial.

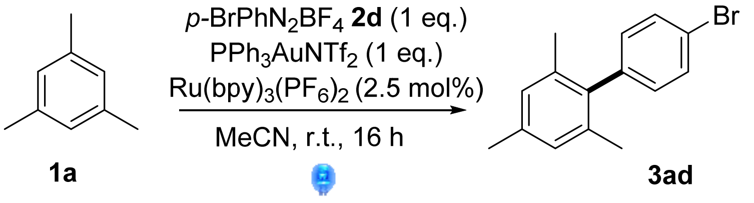

(A)

Monitoring this experiment by ^{1}H and ^{31}P NMR revealed complete consumption of the diazonium salt in favor of the formation of the phosphonium species **VI**.[2] (Figure S1)

Figure S1: ^{1}H NMR monitoring of reaction (A)

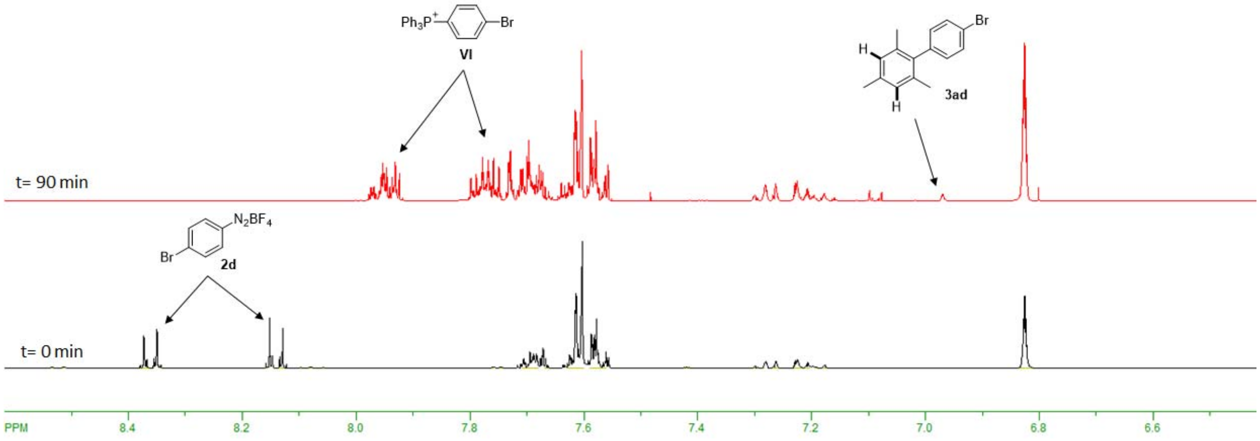

Figure S2: ^{31}P NMR monitoring of reaction (A)

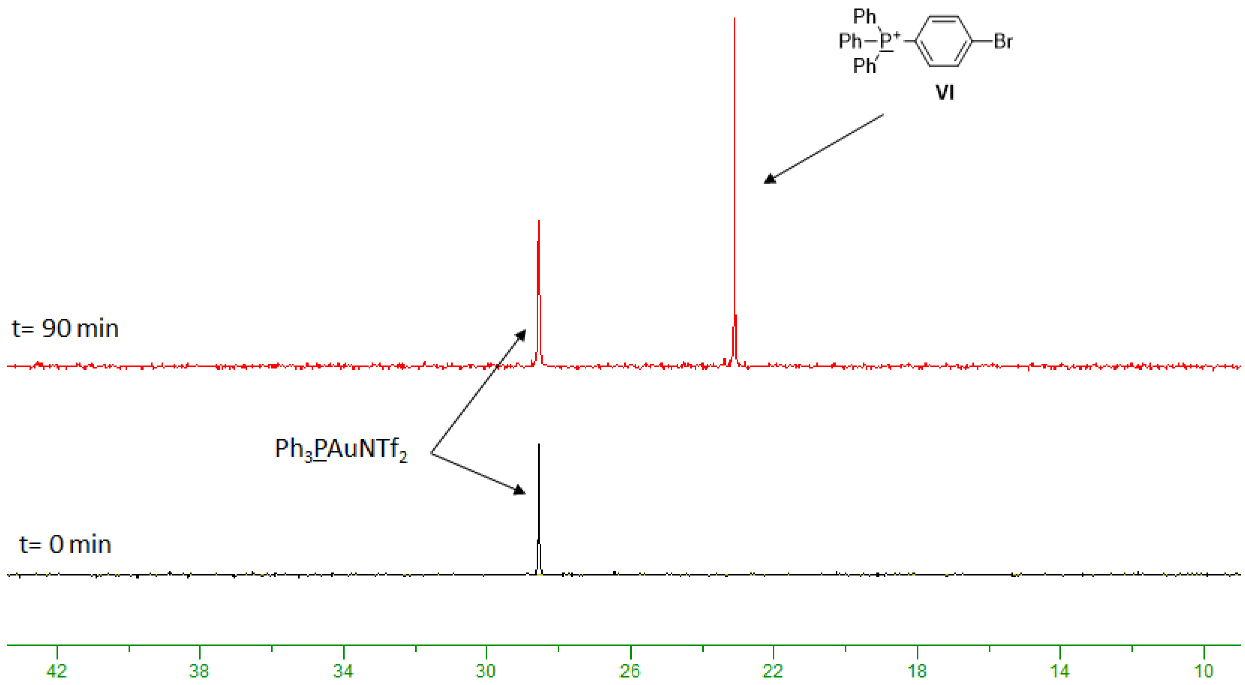

Notably, this phosphonium species **VI** was not observed when the reaction was carried out in the dark, indicating that the photoredox catalysed oxidation process of Au(I) to Au(III) does likely occur *via* a radical addition/single-electron transfer mechanism. Under stoichiometric Au conditions, catalyst deactivation appears to be much faster than the coupling reaction, but this issue can be efficiently addressed by raising the number of equivalents of mesitylene. Therefore, although the phosphonium species **VI** appears in the stoichiometric NMR experiments described above, it does not appear in appreciable amounts in the actual catalytic reaction.

To confirm that the phosphonium species **VI** is innocent and cannot serve as an aryl source, a control reaction using **VI** in place of aryldiazonium was carried out. No reaction was observed.

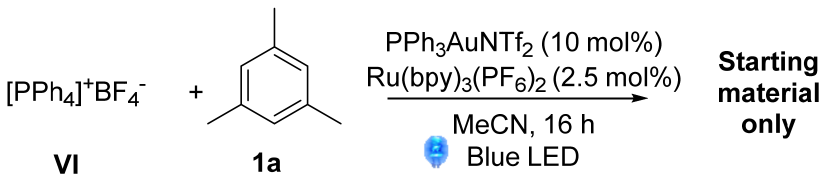

Another experiment was carried to assess the potential formation of an Au(I)-mesitylene intermediate **S2**. A stoichiometric amount of PPh_3AuNTf_2 and mesitylene **1a** were stirred under light irradiation using blue LEDs with a catalytic amount of $Ru(bpy)_3(PF_6)_2$ in deuterated acetonitrile.

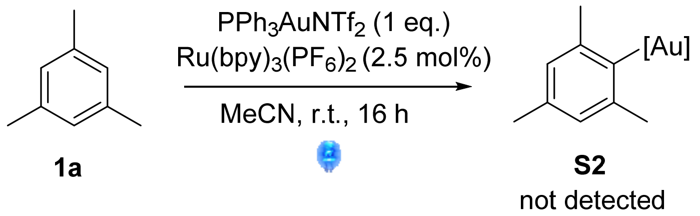

No changes were observed after 16 h, dismissing a possibility of Au(I)-initiated catalysis.

4-Photocatalysis-only control experiments

Two separate control reactions were carried out without gold, to assess the selectivity of the photocatalysis-only reaction setup. Each control reaction afforded a mixture of three inseparable regioisomers, and ratios were determined by ^{1}H NMR. This occurrence highlights the important role of the gold in terms of regioselectivity for this cross-coupling reaction.

4.1-Reported conditions for photocatalysis-only cross-coupling between arenes and diazonium salts.[3]

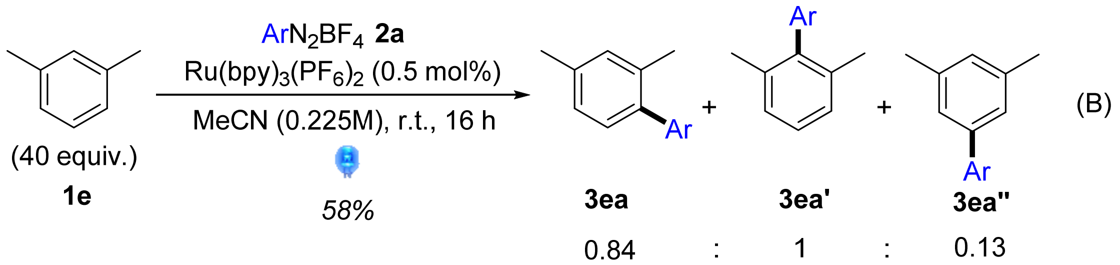

Figure S3: Full spectrum of the isolated mixture of regioisomers for reaction (B)

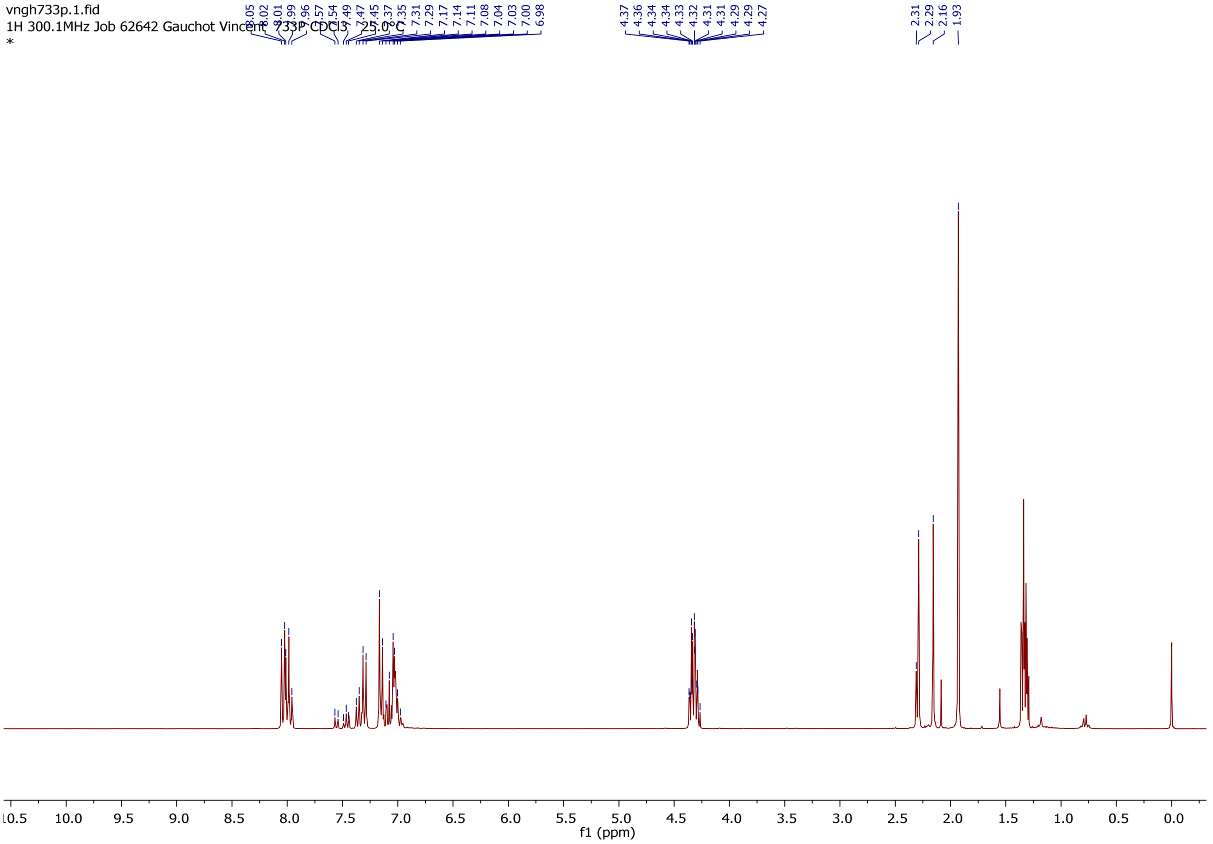

The different regioisomers were identified by looking at the chemical shifts of the methyl groups.[4]

Figure S4: Zoom-in of the methyl peaks region for the above reaction (A)

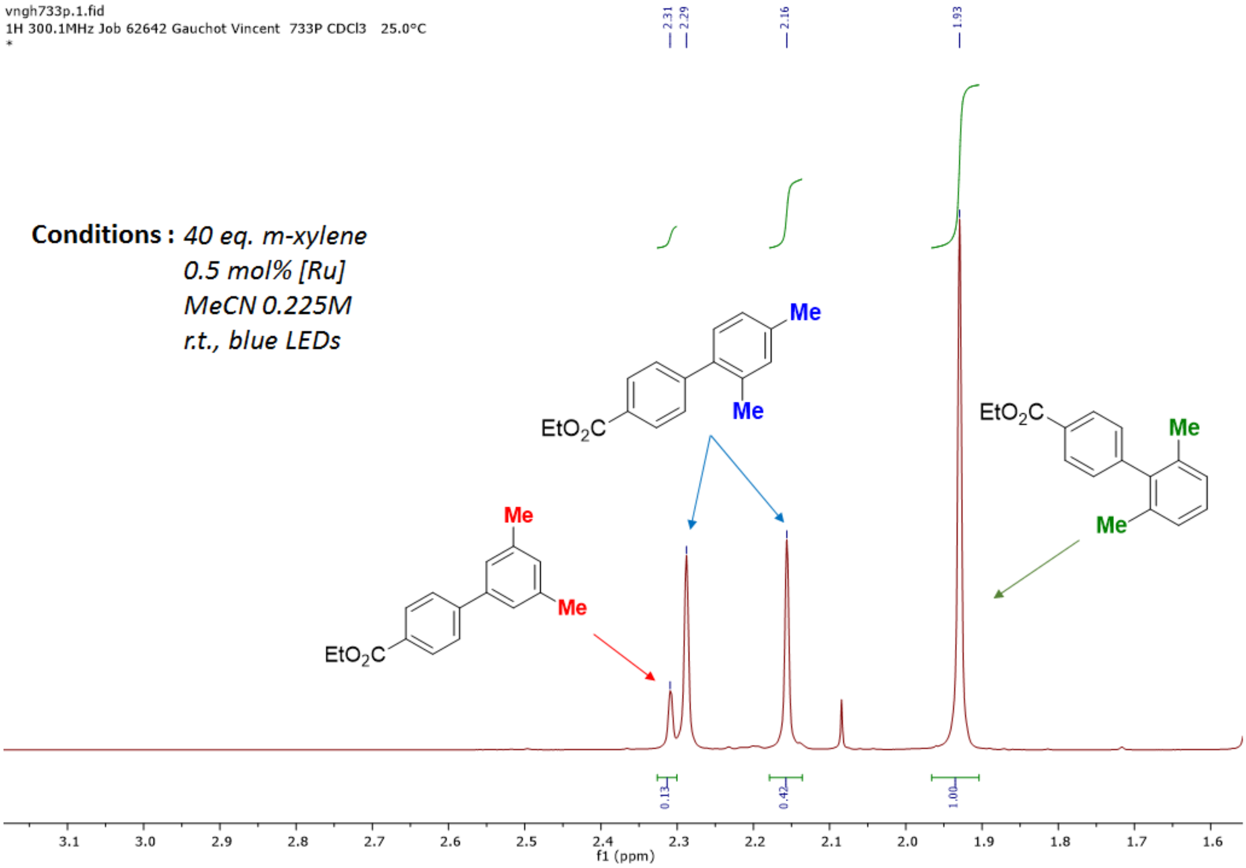

4.2-Photocatalysis-only cross-coupling between arenes and diazonium salts using this paper's optimised conditions (but without gold).

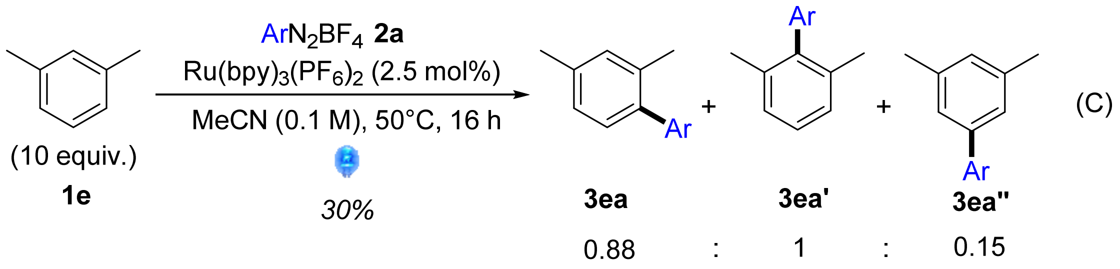

Figure S5: Full spectrum of the isolated mixture of regioisomers for reaction (C)

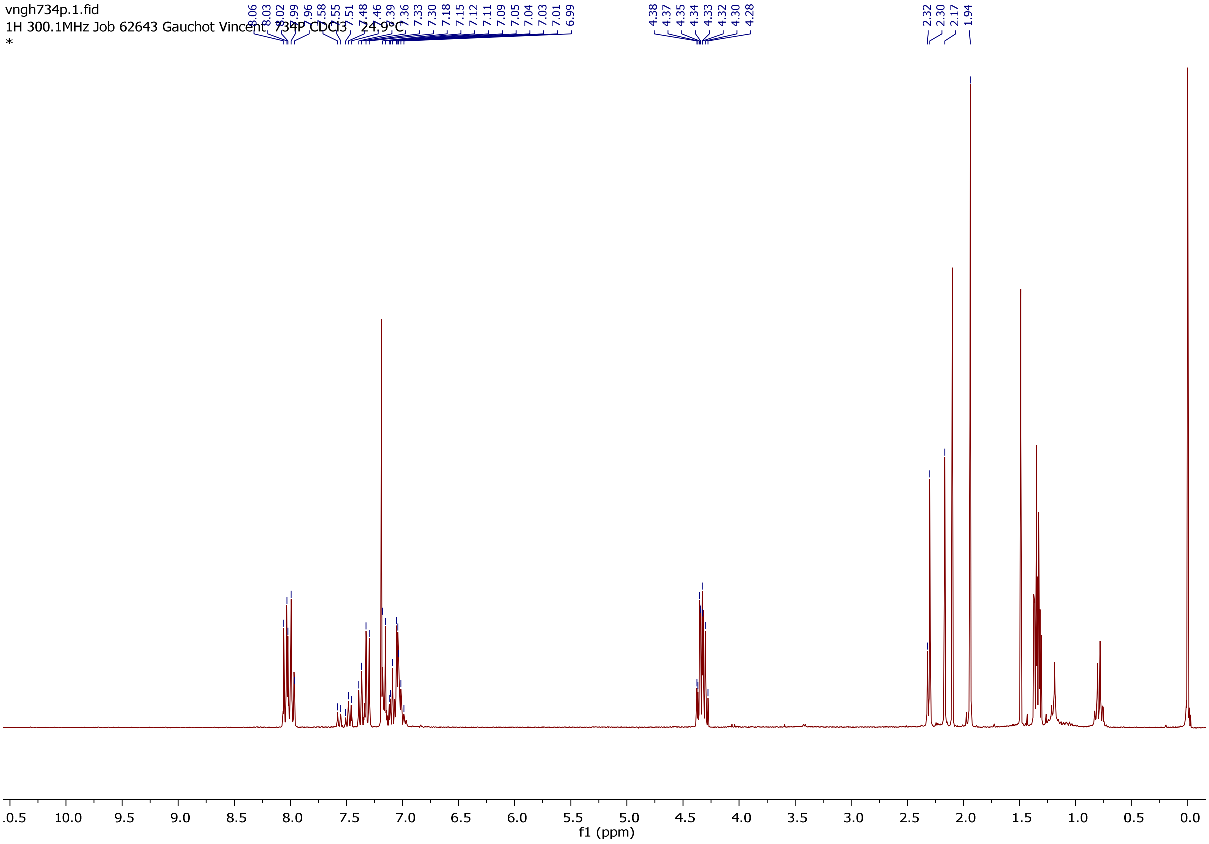

The different regioisomers were identified by looking at the chemical shifts of the methyl groups.[4]

Figure S6: Zoom-in of the methyl peaks region for the above reaction (C)

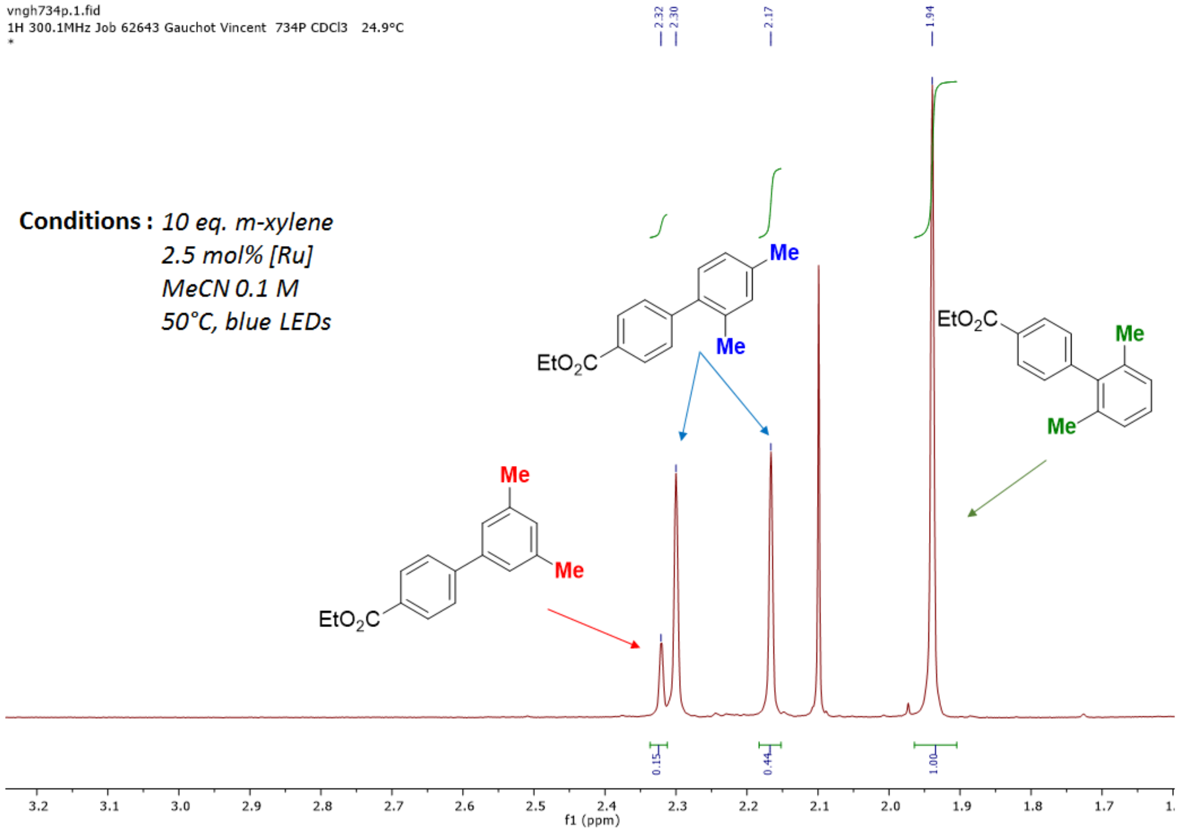

5-Preparation of the substrates

5-1-General procedure for the preparation of diazonium salts

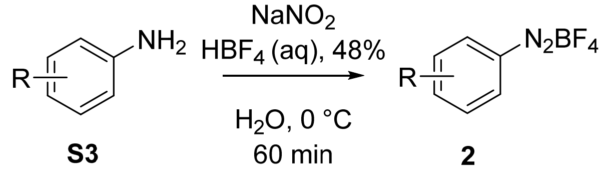

Diazonium salts **2** were prepared according to literature procedures.[5] In a round-bottom flask under air, the corresponding aniline **S3** (3 mmol) was suspended in an aqueous solution of HBF_4 (1 ml) and cooled to 0 °C. A solution of sodium nitrate (207 mg, 3 mmol, 1 equiv.) in water (1 ml) was then added dropwise and the reaction mixture was stirred for 60 min at 0 °C. Upon completion of the reaction, the mixture was filtered and the residue washed once with cold water. The residual solid was then dissolved in a minimum amount of acetone, and precipitation of the diazonium salt was induced by addition of diethyl ether. The solid was filtered and dried under vacuum to afford the corresponding diazonium salt **2**.

5-2 – Preparation of compound 4

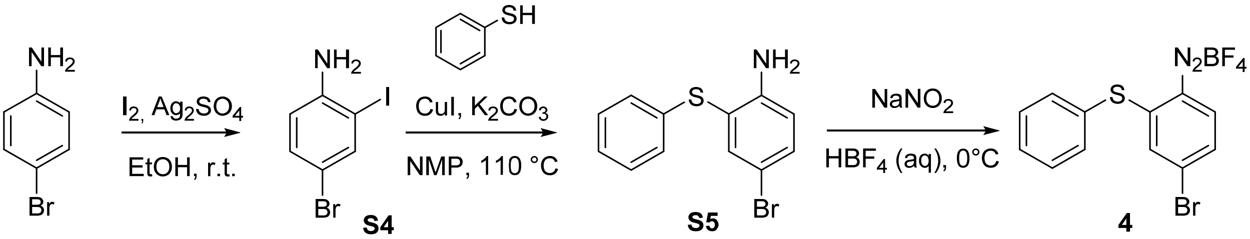

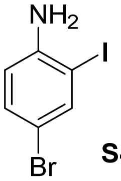

S4

S4 was prepared according to literature.[6] In a round-bottom flask, 4-bromoaniline (860.1 mg, 5 mmol) was dissolved in EtOH (15 ml). Ag_2SO_4 (1.56 g, 5 mmol) and iodine (634.5 mg, 5 mmol) were sequentially added to the solution, and the resulting mixture was stirred overnight at room temperature. Upon completion, the reaction mixture was filtered through Celite, and the volatiles were removed under vacuum. The residue was dissolved in a minimum amount of $CHCl_3$, washed with a saturated aqueous solution of $Na_2S_2O_3$, then washed with brine and dried over magnesium sulfate. Volatiles were removed under vacuum and the residue was purified by column chromatography (Petrol/AcOEt : 85/15) to afford the desired product as a red solid (582.7 mg, 1.39 mmol, 39%). Spectral data was in accordance with literature.

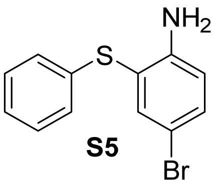

S5

S5 was prepared according to literature.[7] In a 3-ml vial, K_2CO_3 (285.8 mg, 2.07 mmol), 4-bromo-2-iodoaniline **S4** (560.3 mg, 1.88 mmol) and thiophenol (249.3 µl, 2.44 mmol) were suspended in NMP (0.4 ml). CuI (8.9 mg, 2.5 mol%) was added, and the mixture was stirred overnight at 100 °C. Upon completion, the mixture was diluted with acetonitrile and filtered

through Celite. Volatiles were removed under vacuum, and the residue was purified by column chromatography (Petrol/AcOEt : 9/1) to afford the desired compound (403.7 mg, 1.45 mmol, 77%). Spectral data was in accordance with literature.[8]

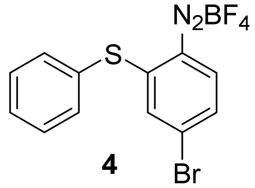

4

4-Bromo-2-(phenylthio)aniline **S5** (439.8 mg, 1.57 mmol) was suspended in HBF_4 (2 ml, 48% in water) and the suspension was cooled to 0 °C. A chilled solution of $NaNO_2$ (108.3 mg, 1.57 mmol) in water (1 ml) was added dropwise, and the resulting mixture was stirred for 1 h at 0 °C. The reaction mixture was filtered, washed with cold H_2O (5 ml) and the solid was collected and dissolved in a minimum amount of acetone. Precipitation was induced by slow addition of ether, the resulting precipitate was filtered off and dried under vacuum to afford compound **4** as a yellow powder (273.4 mg, 0.72 mmol, 46% yield). *This compound will degrade at room temperature, and needs to be stored at 2-8°C.*

^{1}H NMR : (acetone *d-6*, 300 MHz): δ = 8.71 (d, J=9.2 Hz, 1 H), 8.05 (dd, J=9.1, 3.0 Hz, 1 H), 7.78 - 7.87 (m, 2 H), 7.68 - 7.72 (m, 1 H), 7.58 - 7.68 ppm (m, 3 H); **^{13}C NMR** : (acetone *d-6*, 75 MHz): δ = 147.8, 137.5, 134.9, 134.8, 134.4, 132.8, 131.3, 130.8, 127.9, 112.8 ppm; **^{19}F NMR** (acetone *d-6*, 282 MHz): δ =: -150.9 ppm; **IR** : ν_{max}: 3089, 2284, 1556, 1109, 695 cm^{-1}.

5-3 – Preparation of compound 6

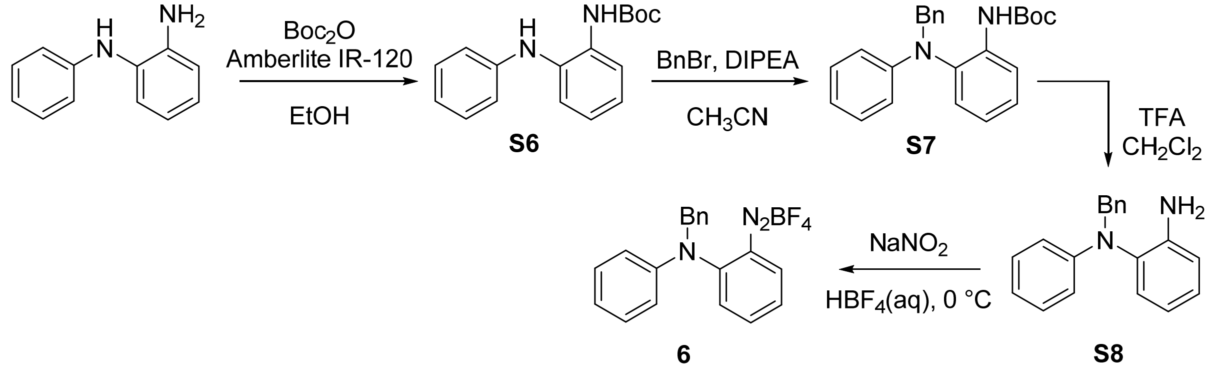

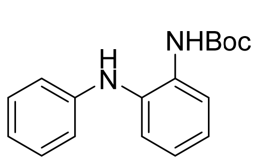

S6

S6 was prepared using a modified procedure from literature.[9] Boc_2O (622 mg, 2.85 mmol) was dissolved in EtOH (2.7 ml), and the solution was warmed up to 50 °C. N-phenyl-*o*-phenylenediamine (500 mg, 2.71 mmol) and Amberlite© IR-120 (35 mg, 7% w/w) were added sequentially, and the resulting mixture was stirred for 2.5 h at 25 °C. Upon completion, CH_2Cl_2 (25 ml) was added, the mixture was filtered, and volatiles removed under vacuum. The residue was dissolved in a minimum of CH_2Cl_2, hexane was added (*ca.* 40 ml) and the solution was sonicated to induce precipitation. Filtration afforded the desired compound as a pink powder (729.4 mg, 2.57 mmol, 90% yield).

^{1}H NMR : ($CDCl_3$, 300 MHz): δ = 7.92 (d, J=8.4 Hz, 1 H), 7.14 - 7.31 (m, 4 H), 7.03 - 7.11 (m, 1 H), 6.97 (br. s, 1 H), 6.86 - 6.94 (m, 1 H), 6.75 - 6.84 (m, 2 H), 5.44 (br. s, 1 H), 1.54 ppm (s, 9 H); **^{13}C NMR** : ($CDCl_3$, 75 MHz): δ = 153.3, 145.5, 133.5, 132.5, 129.4, 125.4, 124.6, 124.0, 120.9,

120.0, 116.0, 80.7, 28.3 ppm **HRMS** found (FTMS + p NSI) $[M + H]^+$ 285.1598, $C_{17}H_{21}N_2O_2$ requires 285.1598.

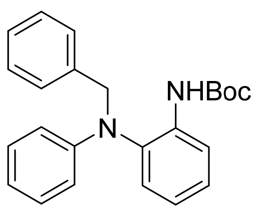

S7

S7 was prepared using a modified procedure from literature.[10] N-phenyl-*o*-phenylene-Boc-diamine **S6** (457.5 mg, 1.61 mmol) was suspended in CH_3CN (7 ml), and DIPEA (328.2 µl, 1.93 mmol)) and BnBr (248.4 µl, 2.09 mmol) were added The resulting mixture was stirred under reflux for 48 h. Volatiles were removed under vacuum, the residual solid was triturated with hexanes, and the hexane layer was removed. Purification by recrystallisation in EtOH afforded the desired compound as a white powder (349.7 mg, 0.93 mmol, 58% yield).

^{1}H NMR : ($CDCl_3$, 300 MHz): δ = 8.15 (d, J=7.7 Hz, 1 H), 7.15 - 7.42 (m, 9 H), 7.07 (dd, J=7.3, 1.5 Hz, 2 H), 6.87 (t, J=7.3 Hz, 1 H), 6.69 - 6.76 (m, 2 H), 4.80 (s, 2 H), 1.46 ppm (s, 9 H) **^{13}C NMR** : ($CDCl_3$, 75 MHz): δ = 152.7, 148.7, 138.1, 136.6, 136.0, 129.2, 128.6, 128.5, 127.6, 127.4, 127.2, 123.6, 119, 2, 118.9, 114.8, 80.4, 56.3, 28.3 ppm; **HRMS** found (FTMS + p NSI) $[M + H]^+$ 375.2065, $C_{24}H_{27}N_2O_2$ requires 375.2067.

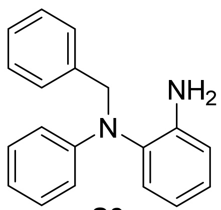

S8

Compound **S7** (250 mg, 0.8 mmol) was dissolved in CH_2Cl_2 (3 ml) and trifluoroacetic acid (1.5 ml, *ca* 25 equiv.) was added. The solution was stirred at room temperature for 7 h. Upon completion, a saturated solution of $NaHCO_3$ was added (10 ml), and the mixture was stirred for 10 min. The layers were separated, the organic phase was washed once with brine, dried over magnesium sulfate, filtered and evaporated. The residue was purified by column chromatography (Petrol/AcOEt: 85/15) to afford the desired compound as an off-white powder (160 mg, 0.58 mmol, 73% yield).

^{1}H NMR : ($CDCl_3$, 300 MHz): δ = 7.15 - 7.56 (m, 9 H), 6.83 - 6.95 (m, 3 H), 6.73 - 6.82 (m, 2 H), 4.97 (s, 2 H), 3.82 ppm (s, 2 H) **^{13}C NMR** : ($CDCl_3$, 75 MHz): δ = 148.0, 144.0, 139.2, 132.9, 129.4, 129.3, 128.7, 127.7, 127.1, 127.0, 119.4, 117.9, 116.5, 113.8, 55.9 ppm; **HRMS** found (FTMS + p NSI) $[M + H]^+$ 275.1545, $C_{19}H_{19}N_2$ requires 275.1543.

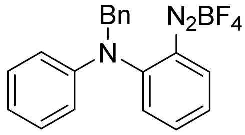

6

Compound **S8** (380 mg, 1.38 mmol) was suspended in HBF_4 (1.3 ml, 48% in water) and the suspension was cooled to 0 °C. A chilled solution of $NaNO_2$ 95.6 mg, 1.38 mmol) in water (1.3 ml) was then added dropwise, and the resulting mixture was stirred for 1 h at 0 °C. The reaction mixture was filtered, washed with cold H_2O (5 ml) and the solid was collected and dissolved in a minimum amount of acetone. Ether was carefully layered on top of the solution, and the flask was placed in the freezer for 16 h. The resulting precipitate was filtered off and dried under vacuum to afford compound **6** as a brown powder (182 mg, 0.48 mmol, 35 % yield).

^{1}**H NMR** : (acetone *d-6*, 300 MHz): δ = 8.10 (dd, J=8.8, 1.5 Hz, 1 H), 7.93 - 8.02 (m, 1 H), 7.76 - 7.83 (m, 2 H), 7.55 - 7.68 (m, 4 H), 7.46 - 7.53 (m, 2 H), 7.29 - 7.44 (m, 3 H), 7.10 - 7.18 (m, 1 H), 5.45 ppm (s, 2 H); ^{13}**C NMR** : (acetone *d-6*, 75 MHz): δ = 153.9, 144.5, 143.0, 136.1, 133.8, 131.6, 131.0, 129.8, 129.2, 128.8, 128.1, 121.8, 119.7 (+1 overlapping peak), 59.2; ^{19}**F NMR** (acetone *d-6*, 282 MHz): δ = -151.4 ppm; **HRMS** found (FTMS + p NSI) $[M]^+$ 286.1341, $C_{19}H_{16}N_3$ requires 286.1339.

6-General procedure for the dual-catalysed C-H functionalisation of electron-rich arenes

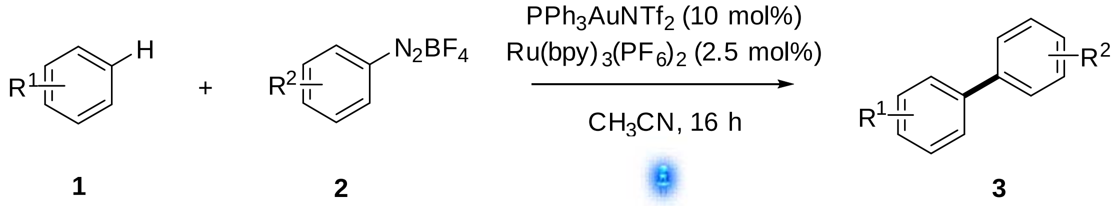

Method A: A Schlenk tube was loaded with the diazonium salt **2** (0.1 mmol), PPh_3AuNTf_2 (7.8 mg, 0.01 mmol, 10 mol%) and $Ru(bpy)_3(PF_6)_2$ (2.2 mg, $2.5x10^{-3}$ mmol). The reaction vessel was wrapped in aluminium foil before CH_3CN (1 ml) was added, and the mixture was degassed using 3 freeze-pump-thaw cycles. The mixture was allowed to warm up to room temperature and the arene **1** (3 equiv.) was added to the reaction vessel. The foil was removed and the reaction mixture was stirred for 16 h at room temperature, under light irradiation using blue LEDs. The mixture was then diluted with EtOAc (10 ml) and the organic phase was washed with distilled water. The aqueous phase was re-extracted once; the combined organic phases were washed with brine, dried over magnesium sulfate and evaporated *in vacuo*. The residue was subsequently purified by chromatography on silica gel using petroleum ether and ethyl acetate to yield the coupled product **3**. Spectral data for all known compounds matched the literature. Spectra for new compounds can be found in Section **6**.

Method B: As above, but reaction was heated to 50 °C instead of RT.

Method C: As above, but 10 equiv. of arene **1** was used and the reaction was heated to 50 °C instead of RT.

Figure S3 : *Representative example of the reaction set-up (Method A). A clean oil bath was used for Methods B and C*

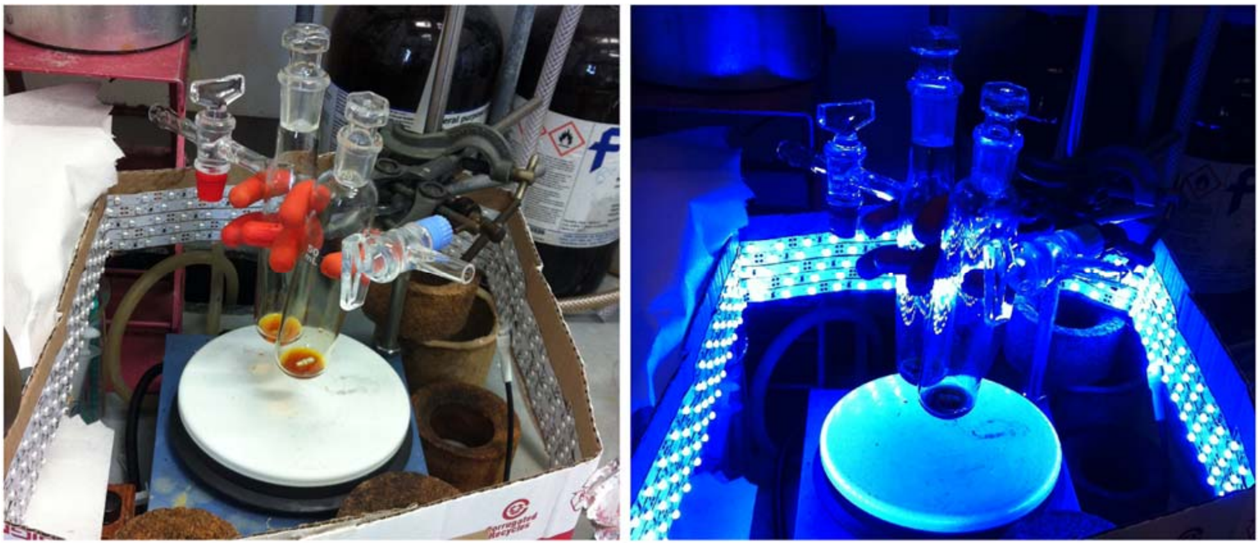

Compound 3aa[11]

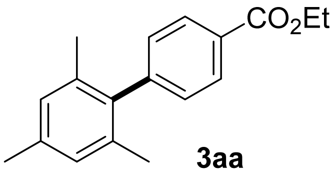
3aa

General procedure (Method A). **Yield** : 19.6 mg, 0.073 mmol, 73%; **^{1}H NMR** : ($CDCl_3$,300 MHz): δ = 8.09 - 8.17 (m, 2 H), 7.23 - 7.29 (m, 2 H), 6.98 (s, 2 H), 4.42 (q, J=7.3 Hz, 1 H), 2.37 (s, 3 H), 2.02 (s, 6 H), 1.45 ppm (t, J=7.3 Hz, 3 H) ; **^{13}C NMR** : ($CDCl_3$,75 MHz): δ = 166.7, 146.2, 138.1, .137.0, 135.5, 129.7, 129.5, 128.9, 128.2, 61.0, 21.0, 20.6, 14.4 ppm; **HRMS :** found (FTMS + p NSI) $[M + H]^+$ 269.1537, $C_{18}H_{21}O_2$ requires 269.1536.

Compound 3ab[12]

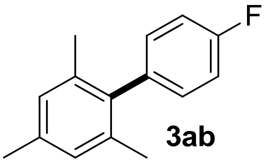
3ab

General procedure (Method A). **Yield** : 17.2 mg, 0.080 mmol, 80%; **^{1}H NMR**: ($CDCl_3$, 300 MHz); δ 7.11 (app. d, *J* = 7.2 Hz, 4H), 6.98 (s, 2 H), 2.37 (s, 3H), 2.04 ppm (s, 6 H); **^{13}C NMR** : ($CDCl_3$, 75 MHz): δ 161.8 (d, *J* = 244.8 Hz), 138.1, 137.0 (d, *J* = 3.5 Hz), 136.9, 136.3, 131.0 (d, *J* = 7.8 Hz), 128.3, 115.4 (d, *J* = 21.1 Hz), 21.2, 20.9 ppm; **HRMS** : found (TOF MS ASAP+) $[M]^+$ 214.1158, $C_{15}H_{15}NO_2$ requires 214.1158.

Compound 3ac[13]

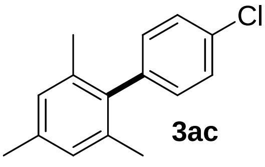
3ac

General procedure (Method A). **Yield** : 14.6 mg, 0.063 mmol, 63%; **^{1}H NMR**: ($CDCl_3$, 300 MHz); δ 7.40 (d, *J* = 8.5 Hz, 2 H), 7.09 (d, *J* = 8.5 Hz, 2 H), 6.95 (s, 2 H), 2.34 (s, 3 H), 2.00 ppm (s, 6 H); **^{13}C NMR** : ($CDCl_3$, 75 MHz): δ 139.5, 137.7, 136.9, 135.9, 132.5, 130.7, 128.7, 128.2, 21.0, 20.7 ppm; **HRMS** : found (TOF MS EI+) $[M]^+$ 230.0862, $C_{15}H_{15}Cl$ requires 230.0869.

Compound 3ad[14]

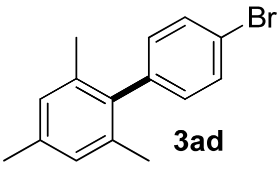
3ad

General procedure (Method A). **Yield** : 20.1 mg, 0.073 mmol, 73%; **^{1}H NMR**: ($CDCl_3$, 300 MHz); δ 7.59 (d, *J* = 8.4 Hz, 2 H), 7.08 (d, *J* = 8.4 Hz, 2 H), 6.99 (s, 2 H), 2.37 (s, 3 H), 2.04 ppm (s, 6 H); **^{13}C NMR** : ($CDCl_3$, 75 MHz): δ 140.0, 137.9, 137.1, 135.9, 131.7, 131.3, 128.3, 120.8, 21.2, 20.9 ppm; **HRMS** : found (TOF MS EI+) $[M]^+$ 274.0356, $C_{15}H_{15}Br$ requires 274.0357.

Compound 3ae

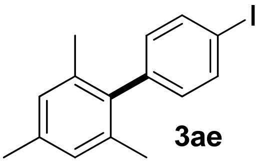
3ae

General procedure (Method A). **Yield** : 16.0 mg, 0.050 mmol, 50%; **^{1}H NMR**: ($CDCl_3$, 300 MHz); δ 7.75 (d, *J* = 8.4 Hz, 2 H), 6.96 (s, 2 H), 6.89 (d, *J* = 8.4 Hz, 2 H), 2.33 (s, 3 H), 2.00 ppm (s, 6 H); **^{13}C NMR** : ($CDCl_3$, 75 MHz): δ 140.8, 137.9, 137.7, 137.1, 135.9, 131.6, 128.3, 92.3, 21.2, 20.9

ppm; **HRMS** : found (TOF MS EI+) $[M]^+$ 322.0225, $C_{15}H_{15}I$ requires 322.0219.

Compound 3af[13]

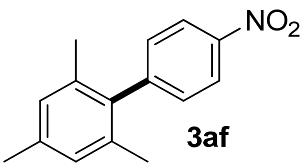

3af

General procedure (Method B). **Yield** : 15.0 mg, 0.062 mmol, 62%; **^{1}H NMR**: ($CDCl_3$, 300 MHz); δ 8.30 (d, *J* = 8.8 Hz, 2 H), 7.35 (d, *J* = 8.8 Hz, 2 H), 6.97 (s, 2 H), 2.35 (s, 3 H), 1.99 ppm (s, 6 H); **^{13}C NMR** : ($CDCl_3$, 75 MHz): δ 148.7, 147.0, 137.9, 136.9, 135.4, 130.6, 128.5, 123.9, 21.2, 20.8 ppm; Found (TOF MS ASAP+) $[M + H]^+$ 242.1181, $C_{15}H_{15}NO_2$ requires 242.1181.

Compound 3ag[15]

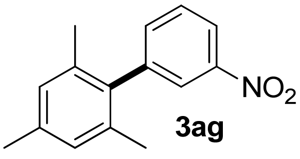

3ag

General procedure (Method B). **Yield** : 14.4 mg, 0.059 mmol, 59%; **^{1}H NMR**: ($CDCl_3$, 300 MHz); δ 8.24 (ddd, *J* = 7.9, 1.8, 1.2 Hz, 1 H), 8.08 (app. t, *J* = 1.8 Hz, 1 H), 7.64 (t, *J* = 7.9 Hz, 2 H), 7.53 (app. dt, *J* = 7.9, 1.4 Hz, 1 H), 6.97 (s, 2 H), 2.35 (s, 3 H), 1.99 ppm (s, 6 H); **^{13}C NMR** : ($CDCl_3$, 75 MHz): δ 148.6, 143.0, 137.8, 136.6, 136.0, 135.7, 129.6, 128.6, 124.6, 121.9, 21.2, 20.8 ppm; **HRMS** : found (TOF MS ASAP+) $[M + H]^+$ 242.1179, $C_{15}H_{15}NO_2$ requires 242.1181.

Compound 3ah[16]

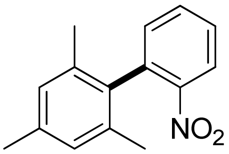

3ah

General procedure (Method A). **Yield** : 8.8 mg, 0.037 mmol, 37%; **^{1}H NMR**: ($CDCl_3$, 300 MHz); δ 8.00 (dd, *J* = 8.1, 1.3 Hz, 1 H), 7.65 (td, *J* = 7.5, 1.3 Hz, 1 H), 7.39 – 7.59 (m, 1 H), 7.17 – 7.30 (m, 1 H), 6.91 (s, 2 H), 2.31 (s, 3 H), 1.95 ppm (s, 6 H); **^{13}C NMR** : ($CDCl_3$, 75 MHz): δ 149.5, 137.6, 136.0, 135.6, 134.3, 133.0, 132.2, 128.3, 128.3, 124.3, 21.2, 20.5 ppm; **HRMS** : found (TOF MS ASAP+) $[M + H]^+$ 242.1179, $C_{15}H_{15}NO_2$ requires 242.1181.

Compound 3ai

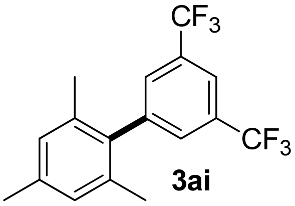

3ai

General procedure (Method C). **Yield** : 19.8 mg, 0.060 mmol, 60%; **^{1}H NMR**: ($CDCl_3$, 300 MHz); δ 7.87 (s, 1 H), 7.63 (s, 2 H), 6.97 (s, 2 H), 2.34 (s, 3 H), 1.98 ppm (s, 6 H); **^{13}C NMR** : ($CDCl_3$, 75 MHz): δ 143.5, 138.2, 136.0, 135.7, 132.0 (q, *J* = 33.2 Hz), 129.9 (m), 128.7, 123.5 (q, *J* = 272.8 Hz), 120.9 (m), 21.0, 20.7 ppm; **HRMS** : found (TOF MS EI+) $[M]^+$ 332.1001, $C_{17}H_{14}F_6$ requires 332.1000.

Compound 3aj

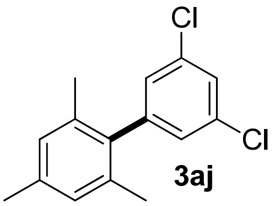
3aj

General procedure (Method C). Yield : 11.6 mg, 0.044 mmol, 44%; **^{1}H NMR**: ($CDCl_3$, 300 MHz); δ 7.34 (t, *J* = 1.9 Hz, 1 H), 7.04 (d, *J* = 1.9 Hz, 2 H), 6.93 (s, 2 H), 2.32 (s, 3 H), 2.01 ppm (s, 6 H); **^{13}C NMR** : ($CDCl_3$, 75 MHz): δ 144.4, 137.6, 136.5, 135.7, 135.1, 128.4, 128.1, 127.0, 21.2, 20.8 ppm; **HRMS** : found (TOF MS EI+) $[M]^+$ 264.0463, $C_{15}H_{14}Cl_2$ requires 264.0472.

Compound 3ak

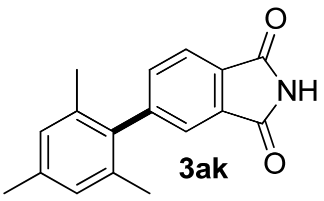
3ak

General procedure (Method A). **Yield** : 17.8 mg, 0.067 mmol, 67%; **^{1}H NMR**: ($CDCl_3$, 300 MHz); δ 8.14 (broad s, 1 H), 7.92 (dd, *J* = 7.6, 0.5 Hz, 1 H), 7.67 (dd, *J* = 1.4, 0.5 Hz, 1H), 7.53 (dd, *J* = 7.6, 1.4 Hz, 1 H), 6.97 (s, 2 H), 2.34 (s, 3H), 1.98 ppm (s, 6H); **^{13}C NMR** : ($CDCl_3$, 75 MHz): δ 168.3, 168.2, 148.6, 137.9, 136.9, 135.7, 135.4, 133.2, 131.1, 128.6, 124.9, 123.9, 21.2, 20.8 ppm; **HRMS** : found (FTMS p NSI+) $[M + H]^+$ 266.1177, $C_{17}H_{16}NO_2$ requires 266.1176.

Compound 3al[13]

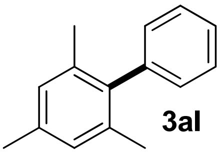
3al

General procedure (Method A). **Yield** : 12.3 mg, 0.062 mmol, 62%; **^{1}H NMR**: ($CDCl_3$, 300 MHz); δ 7.38 – 7.47 (m, 2 H), 7.29 – 7.37 (m, 1 H), 7.11 – 7.18 (m, 2 H), 6.96 (s, 2 H), 2.35 (s, 3 H), 2.02 ppm (s, 6 H); **^{13}C NMR** : ($CDCl_3$, 75 MHz): δ 141.2, 139.2, 136.7, 136.1, 129.4, 128.5, 128.2, 126.6, 21.2, 20.9 ppm; **HRMS** : found (TOF MS EI+) $[M]^+$ 196.1252, $C_{15}H_{16}$ requires 196.1252.

Compound 3am[17]

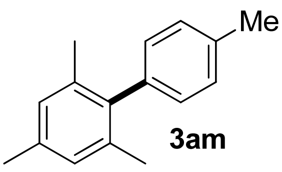
3am

General procedure (Method A). **Yield** : 12.8 mg, 0.048 mmol, 48%; **^{1}H NMR**: ($CDCl_3$, 300 MHz); δ 7.22 (d, *J* = 8.0 Hz, 2 H), 7.04(d, *J* = 8.0 Hz, 2 H), 6.94 (s, 2 H), 2.41 (s, 3 H), 2.34 (s, 3 H), 2.02 ppm (s, 6 H); **^{13}C NMR** : ($CDCl_3$, 75 MHz): δ 139.2, 138.1, 136.5, 136.3, 136.1, 129.3, 129.2, 128.2, 21.4, 21.2, 20.9 ppm; Found (TOF MS EI+) $[M]^+$ 210.1411, $C_{16}H_{18}$ requires 210.1409.

Compound 3an[18]

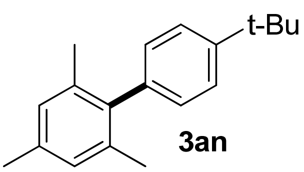

3an

General procedure (Method A). **Yield** : 15.6 mg, 0.048 mmol, 48%; **^{1}H NMR**: ($CDCl_3$, 300 MHz); δ 7.41 (d, *J* = 8.3 Hz, 2 H), 7.07(d, *J* = 8.3 Hz, 2 H), 6.95 (s, 2 H), 2.34 (s, 3 H), 2.03 (s, 6 H), 1.38 ppm (s, 9 H); **^{13}C NMR** : ($CDCl_3$, 75 MHz): δ 149.3, 139.2, 138.0, 136.5, 136.4, 129.0, 128.1, 125.3, 34.7, 31.6, 21.2, 20.9 ppm; **HRMS** : found (TOF MS EI+) $[M]^+$ 252.1878, $C_{19}H_{24}$ requires 252.1882.

Compound 3ao[17]

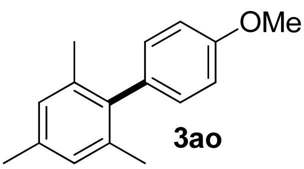

3ao

General procedure (Method C). **Yield** : 6.9 mg (approximately 85% purity), 0.026 mmol, <26%; **^{1}H NMR**: ($CDCl_3$, 300 MHz); δ 7.05 (d, *J* = 8.8 Hz, 2 H), 6.97 (d, *J* = 8.8 Hz, 2 H), 6.93 (s, 2 H), 3.86 (s, 3 H), 2.33 (s, 3 H), 2.01 ppm (s, 6 H); **^{13}C NMR** : ($CDCl_3$, 75 MHz): δ 139.2, 138.1, 136.5, 136.3, 136.1, 129.3, 129.2, 128.2, 21.4, 21.2, 20.9 ppm.

Compound 3ba

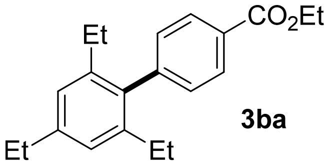

3ba

General procedure (Method A). **Yield** : 10.6 mg, 0.034 mmol, 34%; **^{1}H NMR** : ($CDCl_3$,300 MHz): δ = 8.09 (d, J=8.4 Hz, 2 H), 7.27 (d, J=8.4 Hz, 2 H), 6.99 (s, 2H), 4.41 (q, J=7.0 Hz, 2 H), 2.68 (q, J=7.3 Hz, 2 H), 2.28 (q, J=7.3 Hz, 4 H), 1.42 (t, J=7.0 Hz, 3 H), 1.29 (t, J=7.3 Hz, 3 H), 1.00 ppm (t, J=7.3 Hz, 6 H); **^{13}C NMR** : ($CDCl_3$,75 MHz): δ = 166.7, 145.7, 143.8, 141.8, 137.2, 129.9, 129.3, 128.9, 125.3, 61.0, 28.7, 26.8, 15.6, 15.5, 14.4 ppm; **HRMS :** found (ASAP) $[M + H]^+$ 311.2005, $C_{21}H_{27}O_2$ requires 311.2011.

Compound 3da

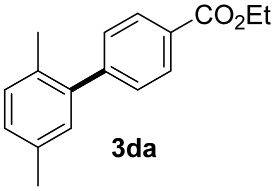

3da

General procedure (Method C).**Yield**: 20.9 mg, 0.082 mmol, 82%; **^{1}H NMR** : ($CDCl_3$,300 MHz): δ = 8.08 (d, J=8.6 Hz, 2 H), 7.39 (d, J=8.6 Hz, 2 H), 7.17 (d, J=7.7 Hz, 1 H), 7.11 (dd, J=7.7, 1.6 Hz, 1 H), 7.05 (d, J=1.6 Hz, 1 H), 4.41 (q, J=7.3 Hz, 2 H), 2.36 (s, 3 H), 2.22 (s, 3 H), 1.42 ppm (t, J=7.3 Hz, 3 H); **^{13}C NMR** : ($CDCl_3$,75 MHz): δ = 166,.6, 146.9, 140.8, 135.4, 132.0, 130.4, 130.2, 129.3, 129.2, 128.9, 128.5, 61.0, 21.0, 19.9, 14.4 ppm; **HRMS :** found (ASAP) $[M + H]^+$ 255.1385, $C_{17}H_{19}O_2$ requires 255.1586.

Compound 3ea

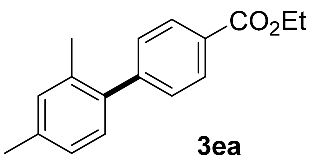

3ea

General procedure (Method C).**Yield** : 20.9 mg, 0.082 mmol, 82%; ^{1}**H NMR** : ($CDCl_3$,300 MHz): δ = 8.08 (d, J=8.5 Hz, 2 H), 7.39 (d, J=8.5 Hz, 2 H), 7.05 - 7.14 (m, 3 H), 4.41 (q, J=7.0 Hz, 2 H), 2.37 (s, 3 H), 2.24 (s, 3 H), 1.42 ppm (t, J=7.0 Hz, 3 H); ^{13}**C NMR** : ($CDCl_3$,75 MHz): δ = 166.6, 146.7, 138.1, 137.6, 135.0, 131.3, 129.5, 129.4, 129.3, 128.8, 126.6, 60.9, 21.1, 20.3, 14.4 ppm; **HRMS :** found (ASAP) $[M + H]^+$ 255.1385, $C_{17}H_{19}O_2$ requires 255.1385.

Compound 3fa[19]

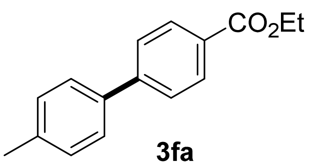

3fa

General procedure (Method C). **Yield** : 13.5 mg, 0.056 mmol, 56%; ^{1}**H NMR** : ($CDCl_3$,300 MHz): δ = 8.13 (d, J=8.4 Hz, 2 H), 7.68 (d, J=8.4 Hz, 2 H), 7.56 (d, J=8.1 Hz, 2 H), 7.31 (d, J=8.8 Hz, 2 H), (q, J=7.3 Hz, 2 H), 2.44 (s, 3 H), 1.45 ppm (t, J=7.3 Hz, 3 H); ^{13}**C NMR** : ($CDCl_3$,75 MHz): δ = 166.6, 145.5, 138.1, 137.2, 130.1, 129.7, 129.0, 127.1, 126.8, 60.9, 20.1, 14.4 ppm; **HRMS :** found (ASAP) $[M + H]^+$ 241.1228, $C_{16}H_{17}O_2$ requires 241.1227.

Compound 3ga

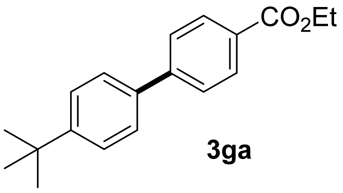

3ga

General procedure (Method C).**Yield** : 20.1 mg, 0.71 mmol, 71% (mixture of inseparable *para/ortho* isomers 5.7:1); ^{1}**H NMR** (major isomer only): ($CDCl_3$,300 MHz): δ = 8.11(d, J=8.6 Hz, 2 H), 7.66 (d, J=8.6 Hz, 2 H), 7.58 (d, J=8.7 Hz, 2 H), 7.50 (d, J=8.7 Hz, 2 H), 4.41 (q, J=7.0 Hz, 2 H), 1.42 (t, J=7.0 Hz, 3 H), 1.37 ppm (s, 9 H); ^{13}**C NMR** : ($CDCl_3$,75 MHz): δ = 166.6, 151.3, 145.4, 137.1, 130.1, 129.0, 126.9, 126.8, 125.9, 60.9, 34.7, 31.3, 14.4 ppm; **HRMS :** found (ASAP) $[M + H]^+$ 283.1696, $C_{19}H_{23}O_2$ requires 283.1698.

Compound 3ha[20]

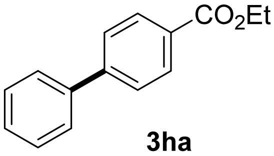

3ha

General procedure (Method C). **Yield** : 13.1 mg, 0.058 mmol, 58%; ^{1}**H NMR** : ($CDCl_3$,300 MHz): δ = 8.15 (d, J=8.8 Hz, 2 H), 7.70 (d, J=8.4 Hz, 2 H), 7-62 – 7.67 (m, 2H), 7.36 - 7.58 (m, 3 H), 4.44 (q, J=7.3 Hz, 2 H), 1.45 ppm (t, J=7.3 Hz, 3 H); ^{13}**C NMR** : ($CDCl_3$,75 MHz): δ = 166.5, 145.6, 140.1, 130.1, 129.3, 128.9, 128.1, 128.3, 127.0, 61.0, 14.4 ppm; **HRMS :** found (ASAP) $[M + H]^+$ 227.1072, $C_{16}H_{14}O_2$ requires 227.1074.

Compound 3ia

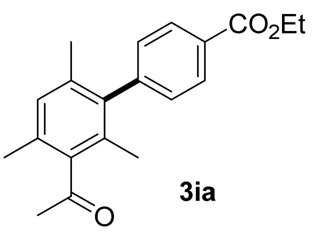

General procedure (Method C). **Yield** : 16.2 mg, 0.052 mmol, 52%; **^{1}H NMR**: ($CDCl_3$,300 MHz): δ = 8.11 (d, J=8.4 Hz, 2 H), 7.20 (d, J=8.4 Hz, 2 H), 6.97 (s, 1 H), 4.41 (q, J=7.3 Hz, 2 H), 2.49 (s, 3 H), 2.26 (s, 3 H), 1.96 (s, 3 H), 1.88 (s, 3 H), 1.42 ppm (t, J=7.3 Hz, 3 H); **^{13}C NMR**: ($CDCl_3$,75 MHz): δ = 208.7, 166.5, 145.4, 140.7, 139.1, 136.2, 131.4, 129.9, 129.4, 129.3, 129.2, 127.2, 61.0, 32.4, 20.7, 18.9, 17.5, 14.4 ppm; **HRMS :** found (FTMS + p NSI) $[M + H]^+$ 311.1642, $C_{20}H_{23}O_3$ requires 311.1642.

Compound 3ja

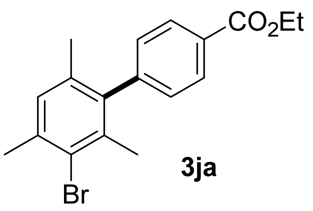

General procedure (Method C). **Yield** : 19.8 mg, 0.057 mmol, 57%; **^{1}H NMR** : ($CDCl_3$,300 MHz): δ 8.11 (d, J=8.5 Hz, 2 H), 7.19 (d, J=8.5 Hz, 2 H), 7.02 (s, 1 H), 4.41 (q, J=7.3 Hz, 2 H), 2.44 (s, 3 H), 2.10 (s, 3 H), 1.92 (s, 3 H), 1.42 ppm (t, J=7.3 Hz, 3 H); **^{13}C NMR** : ($CDCl_3$,75 MHz): δ = 166.5, 146.1, 139.9, 137.4, 135.6, 134.3, 129.9, 129.6, 129.3, 125.5, 61.0, 24.0, 21.9, 20.5, 14.4 ppm; **HRMS :** found (FTMS + p NSI) $[M + H]^+$ 347.0644, $C_{18}H_{20}BrO_2$ requires 347.0641.

Compound 3ka

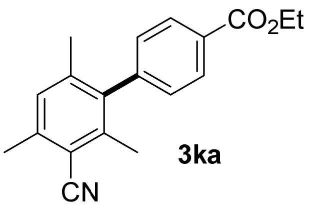

General procedure (Method C).**Yield** : 10.8 mg, 0.037 mmol, 37%; **^{1}H NMR** : ($CDCl_3$,300 MHz): δ = 8.13 (d, J=8.6 Hz, 2 H), 7.17 (d, J=8.6 Hz, 2 H), 7.07 (s, 1 H), 4.42 (q, J=7.3 Hz, 2 H), 2.54 (s, 3 H), 2.19 (s, 3 H), 2.01 (s, 3 H), 1.42 ppm (t, J=7.3 Hz, 3 H); **^{13}C NMR** : ($CDCl_3$,75 MHz): δ = 166.4, 144.2, 141.3, 140.9, 139.7, 139.3, 130.1, 129.7, 129.2, 129.1, 117.6, 111.5, 61.1, 21.2, 20.7, 19,5, 14.4 ppm; **HRMS :** found (ASAP) $[M + H]^+$ 294.1490, $C_{19}H_{20}NO_2$ requires 294.1489.

Compound 3la

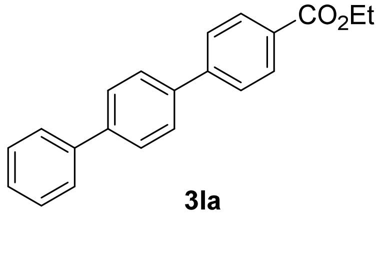

General procedure (Method C). **Yield** : 17.1 mg, 0.56 mmol, 56%; **^{1}H NMR**: ($CDCl_3$,300 MHz): δ = 8.17 (d, J=8.8 Hz, 2 H), 7.72 - 7.79 (m, 6 H), 7.68 (d, J=7.3 Hz, 2 H), 7.50 (t, J=7.0 Hz, 2 H), 7.38 - 7.45 (m, 1 H), 4.45 (q, J=7.0 Hz, 2 H), 1.46 ppm (t, J=7.0 Hz, 3 H); **^{13}C NMR** : ($CDCl_3$,75 MHz): δ = 166.5, 145.0, 141.0, 140.5, 138.9, 130.1, 129.3, 128.9, 127.7, 127.6, 127.1 (+1 overlapping peak), 126.9, 61.0, 14.4 ppm; **HRMS :** found (FTMS + p NSI) $[M + H]^+$ 303.1380, $C_{21}H_{19}O_2$ requires 303.1380.

Compound 5

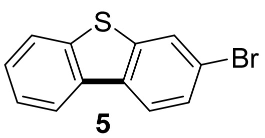
5

Reaction carried out a room temperature. **Yield** : 21.8 mg, 0.083 mmol, 83%; **^{1}H NMR** : ($CDCl_3$,300 MHz): δ = 8.11 - 8.20 (m, 1 H), 8.04 (d, J=5.5 Hz, 1 H), 8.02 (s, 1H), 7.83 - 7.92 (m, 1 H), 7.57 - 7.63 (m, 1 H), 7.46 - 7.56 ppm (m, 2 H); **^{13}C NMR** : ($CDCl_3$, 75 MHz): δ = 141.0, 139.3, 134.8, 134.5, 127.8, 127.1, 125.4, 124.7, 122.8, 122.6, 121.6, 120.3 ppm; **HRMS :** found (EI) $[M]^+$ 261.9448, $C_{12}H_7BrS$ requires 261.9440.

Compound 7[21]

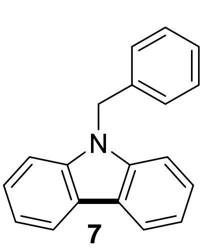
7

Reaction carried out a room temperature. **Yield** : 22.1 mg, 0.082 mmol, 82%; **^{1}H NMR** : ($CDCl_3$,300 MHz): δ = 8.18 (d, J=7.7 Hz, 2 H), 7.44 - 7.52 (m, 2 H), 7.41 (d, J=8.1 Hz, 2 H), 7.25 - 7.35 (m, 5 H), 7.16 - 7.22 (m, 2 H), 5.57 ppm (s, 2 H); **^{13}C NMR** : ($CDCl_3$, 75 MHz): δ = 139.6, 136.2, 127.7, 126.4, 125.4, 124.8, 122.0, 119.4, 118.2, 107.9, 45.5 ppm; **HRMS :** found (FTMS + p NSI) $[M+H]^+$ 258.1280, $C_{19}H_{16}N$ requires 258.1377.

*Additional Data for compounds **3ma'** and **3oa'***

Attempted formation of 3ma

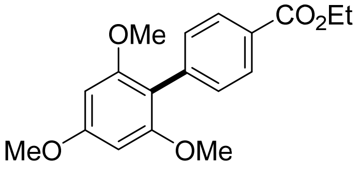

3ma

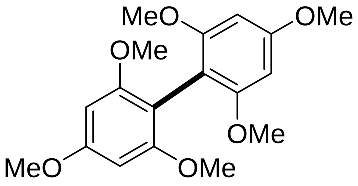

3ma'

Compound **3ma** was only observed in trace amounts when subjected to reaction **conditions A**. Interestingly, the major product **3ma'** formed under these conditions was the oxidative homocoupling of 1,3,5-trimethoxybenzene.

Yield: 7.8 mg, 0.023 mmol, 54%.**^{1}H NMR** : ($CDCl_3$,300 MHz): δ = 6.16 (s, 4H), 3.77 (s, 6H), 3.64 (s, 12H); **HRMS :** found (ASAP) $[M+H]^+$ 335.1485, $C_{18}H_{23}O_6$ requires 335.1488. Data was in accordance with literature.[22]

When submitted to reaction **conditions C**, the azo coupling[23] product **3 ma''** is the major product.

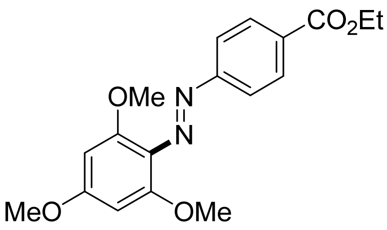

3ma''

Yield: <50% (impure).**^{1}H NMR** : ($CDCl_3$,300 MHz): δ = 8.18 (d, J=8.8 Hz, 2 H), 7.88 (d, J=8.4 Hz, 2 H), 6.27 (s, 2 H), 4.43 (q, J=6.6 Hz, 2 H), 3.90 - 3.97 (m, 9 H), 1.44 ppm (d, J=7.3 Hz, 3 H); **HRMS :** found (FTMS + p NSI) $[M + H]^+$: 345.1446, $C_{18}H_{22}N_2O_5$ requires 345.1445.

Attempted formation of 3oa:

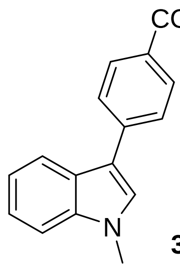

3oa *not observed*

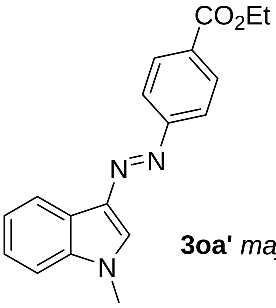

3oa' *major compound*

As shown in Table 3, compound **3oa** was not observed, and the azo compound[24] 3**oa'** was always observed as the major compound (NMR yield: up to 92%).**^{1}H NMR** : ($CDCl_3$,300 MHz): δ = 8.55 - 8.64 (m, 1 H), 8.20 (d, J=8.8 Hz, 2 H), 7.89 - 7.96 (m, 3 H), 7.34 - 7.45 (m, 3 H), 4.44 (q, J=7.3 Hz, 2 H), 1.46 ppm (t, J=8.1 Hz, 3 H); **HRMS :** found (FTMS + p NSI) $[M + H]^+$: 308.1395, $C_{18}H_{18}N_3O_2$ requires 308.1394.

7 – RELEVANT NMR SPECTRA

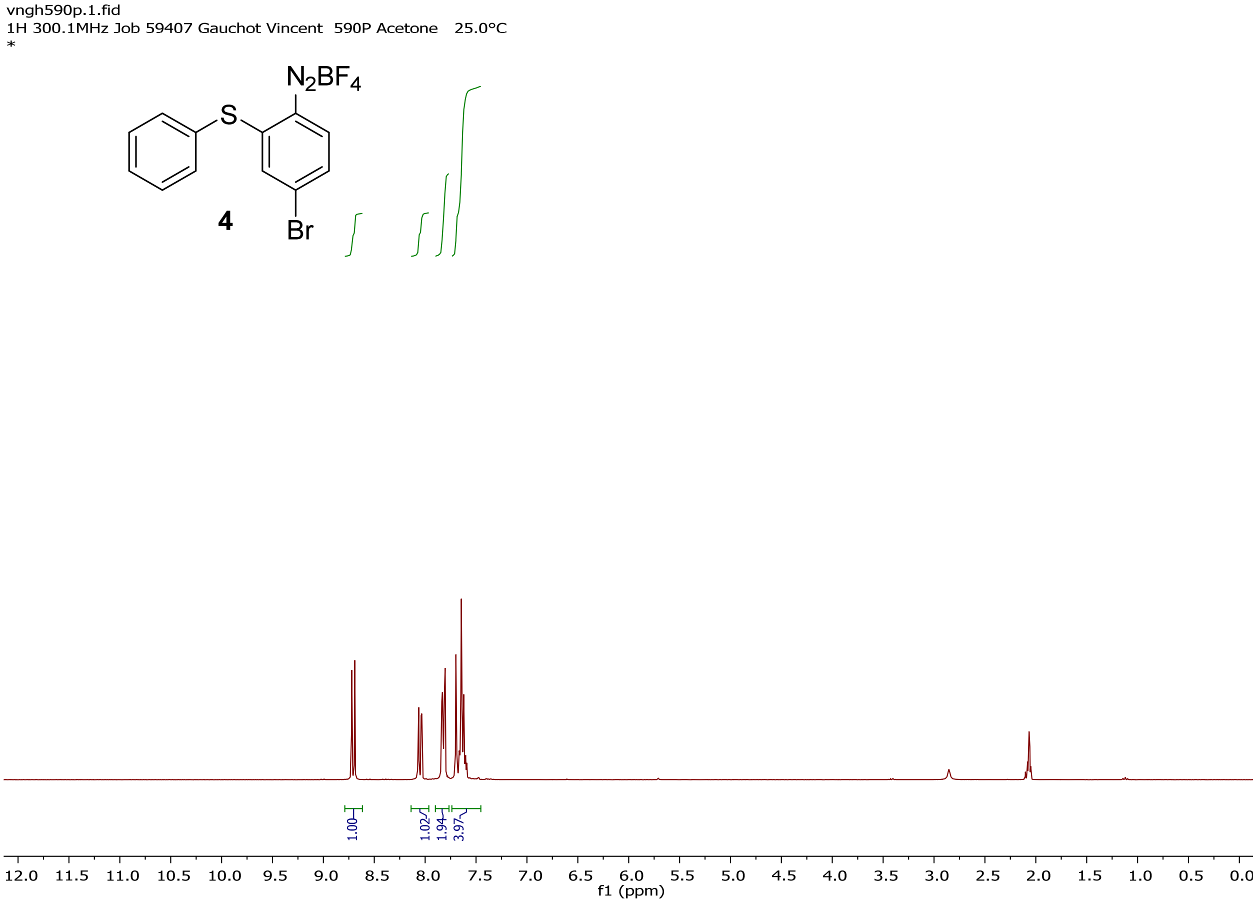

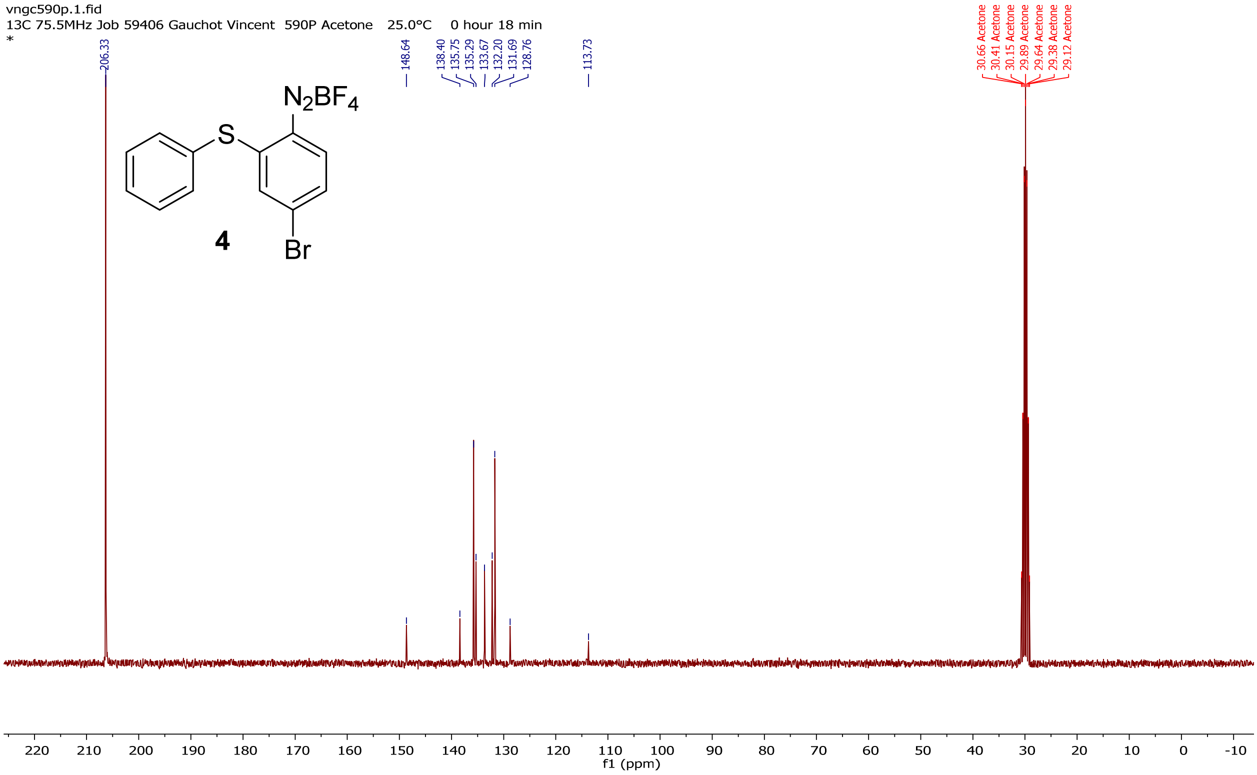

vngf590p.1.fid
19F 282.4MHz Job 59408 Gauchot Vincent 590P Acetone 25.0°C 0hours 1min
*

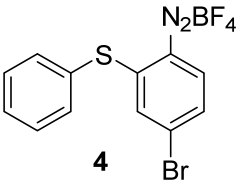

4

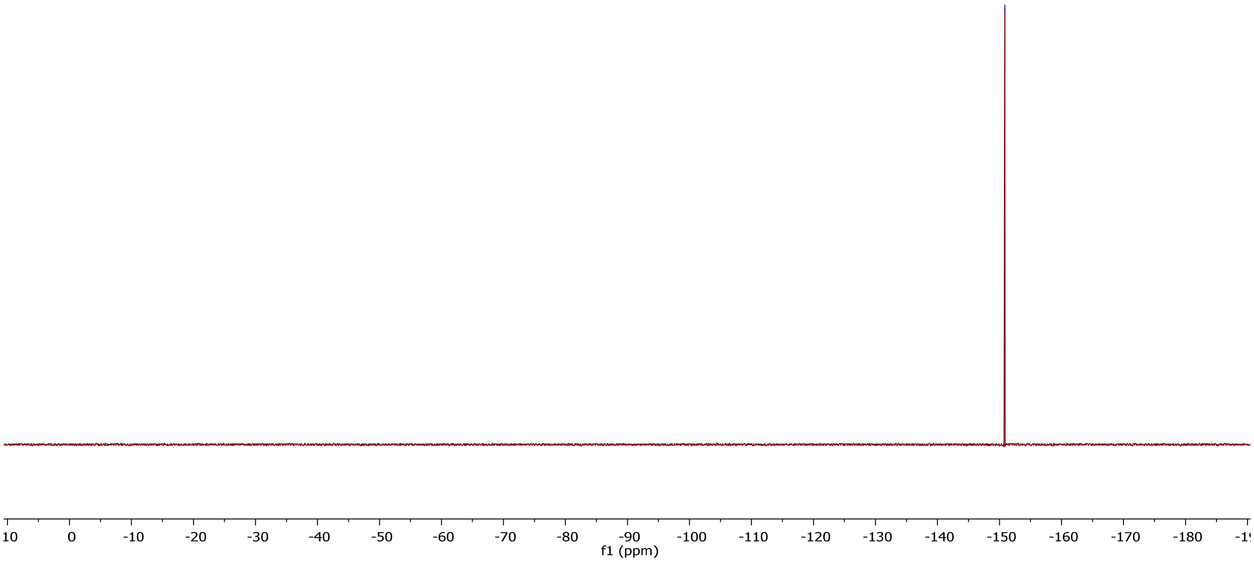

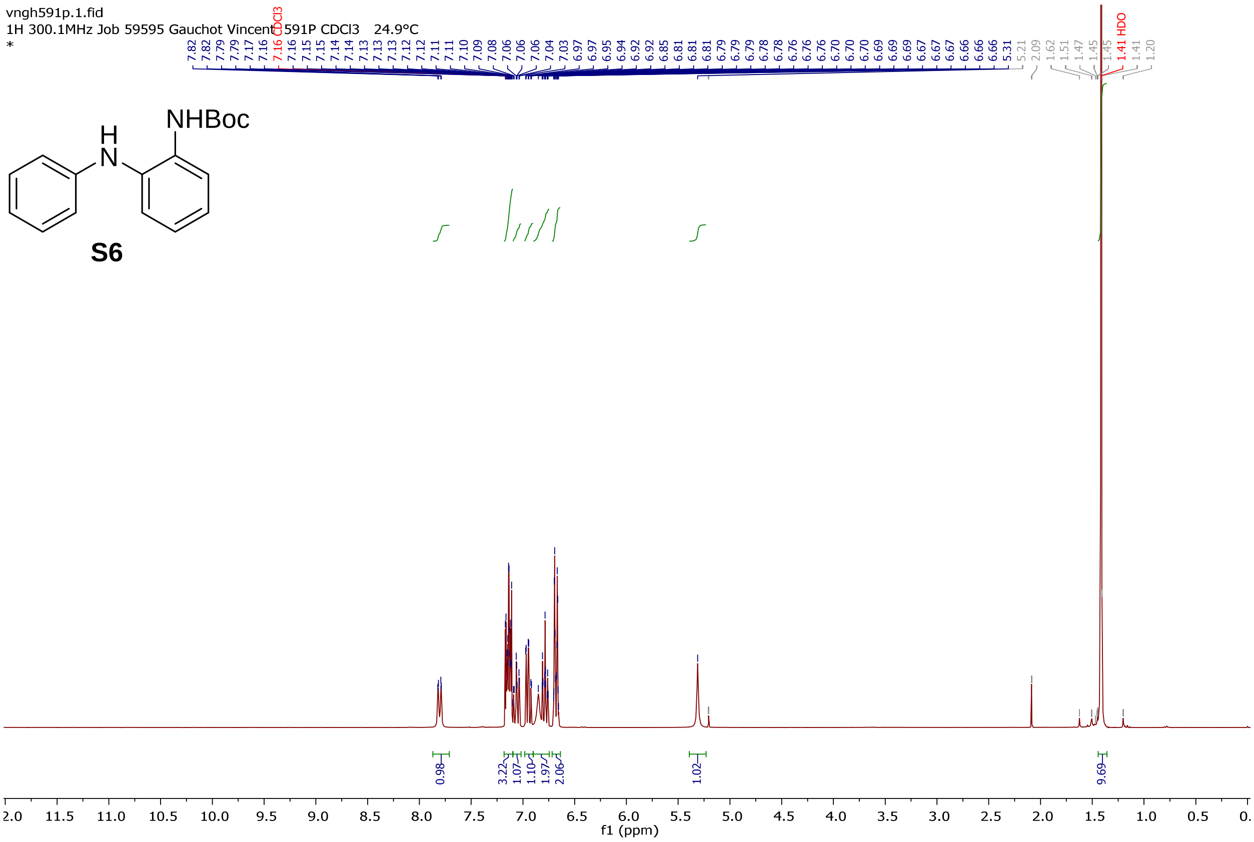
vngh591p.1.fid
1H 300.1MHz Job 59595 Gauchot Vincent 591P CDCl3 24.9°C
*
7.16 CDCl3
1.41 HDO
NHBoc
H
N
S6
0.98
3.22
1.07
1.10
1.97
2.06
1.02
9.69
f1 (ppm)

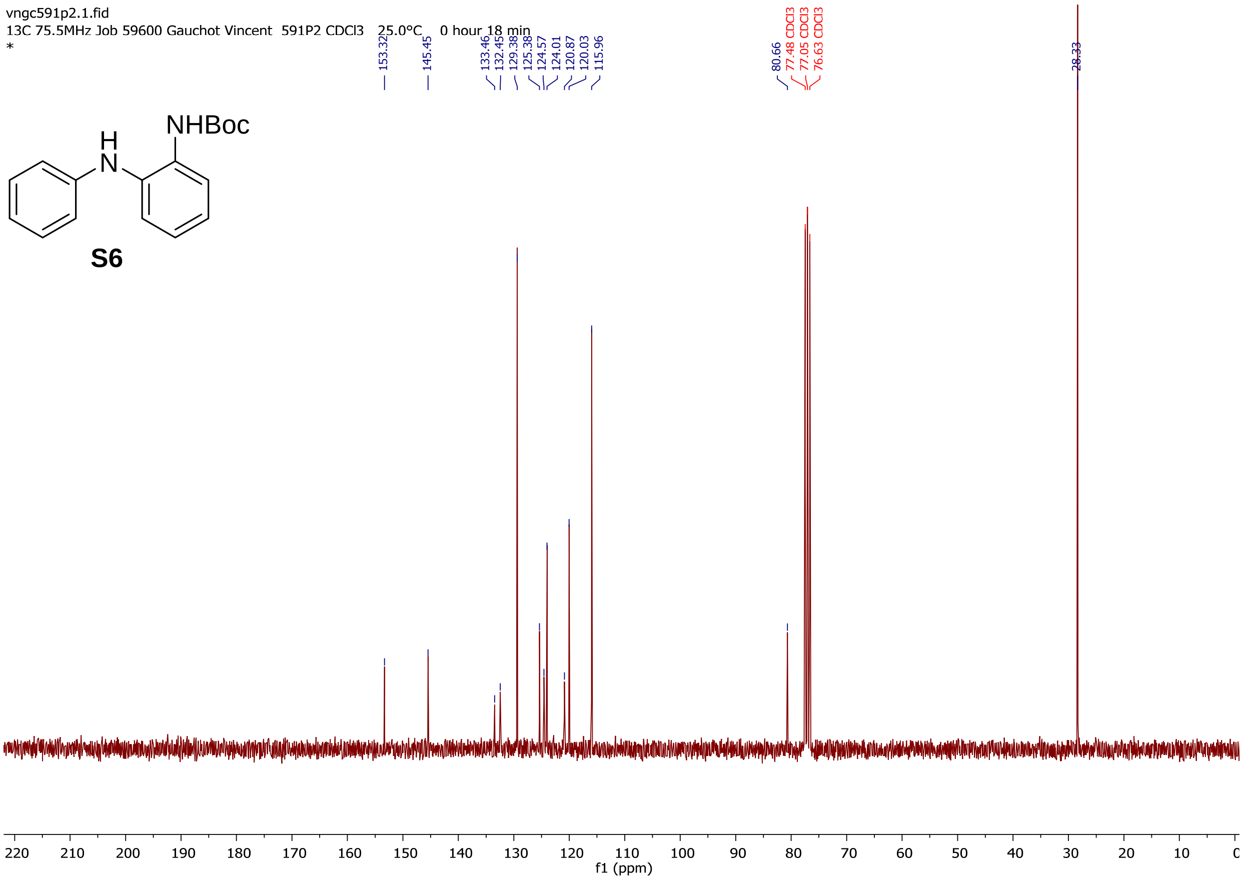
vngc591p2.1.fid
13C 75.5MHz Job 59600 Gauchot Vincent 591P2 CDCl3 25.0°C 0 hour 18 min
*
153.32
145.45
133.46
132.45
129.38
125.38
124.57
124.01
120.87
120.03
115.96
80.66
77.48 CDCl3
77.05 CDCl3
76.63 CDCl3
28.33
NHBoc
H
N
S6
f1 (ppm)

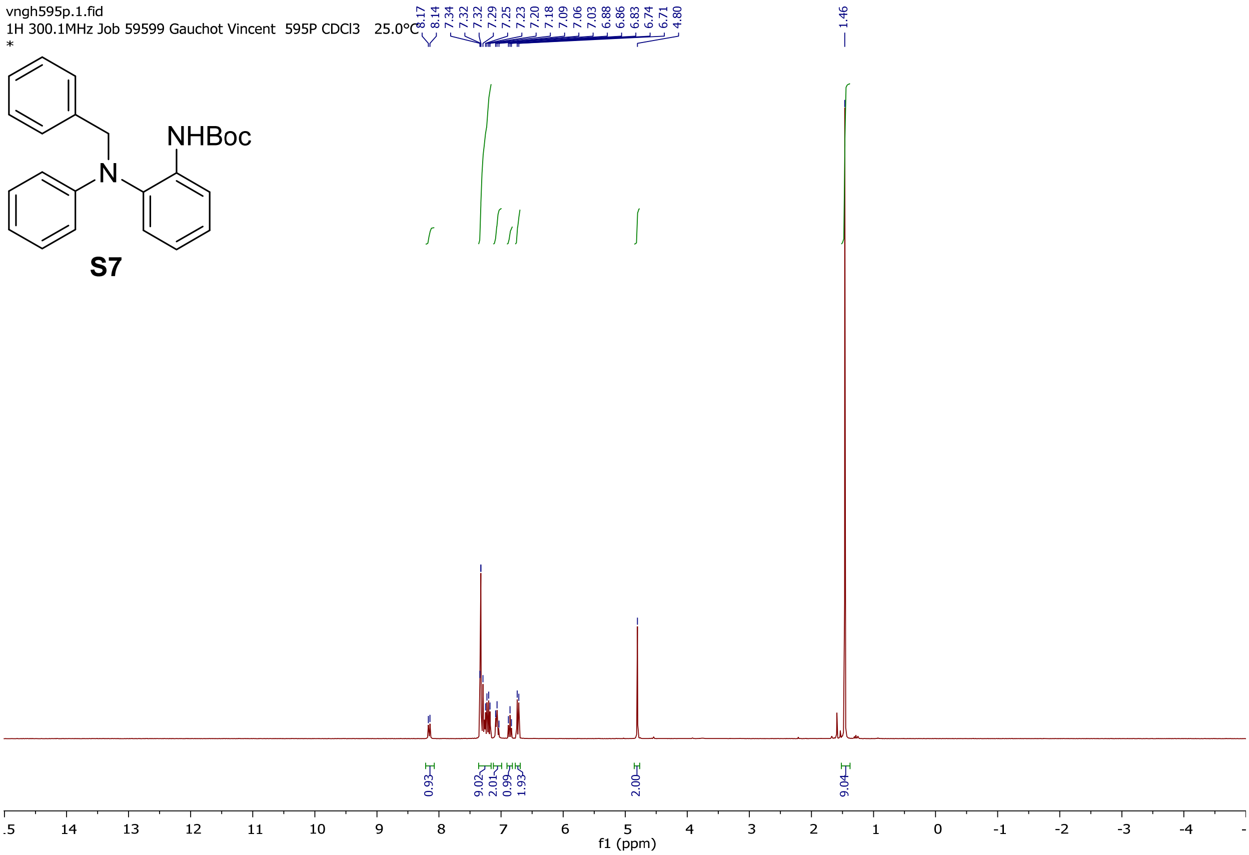
vngh595p.1.fid
1H 300.1MHz Job 59599 Gauchot Vincent 595P CDCl3 25.0ºC
*
NHBoc
N
S7
8.17
8.14
7.34
7.32
7.32
7.29
7.25
7.23
7.20
7.18
7.09
7.06
7.03
6.88
6.86
6.83
6.74
6.71
4.80
1.46
0.93
9.02
2.01
0.99
1.93
2.00
9.04
f1 (ppm)

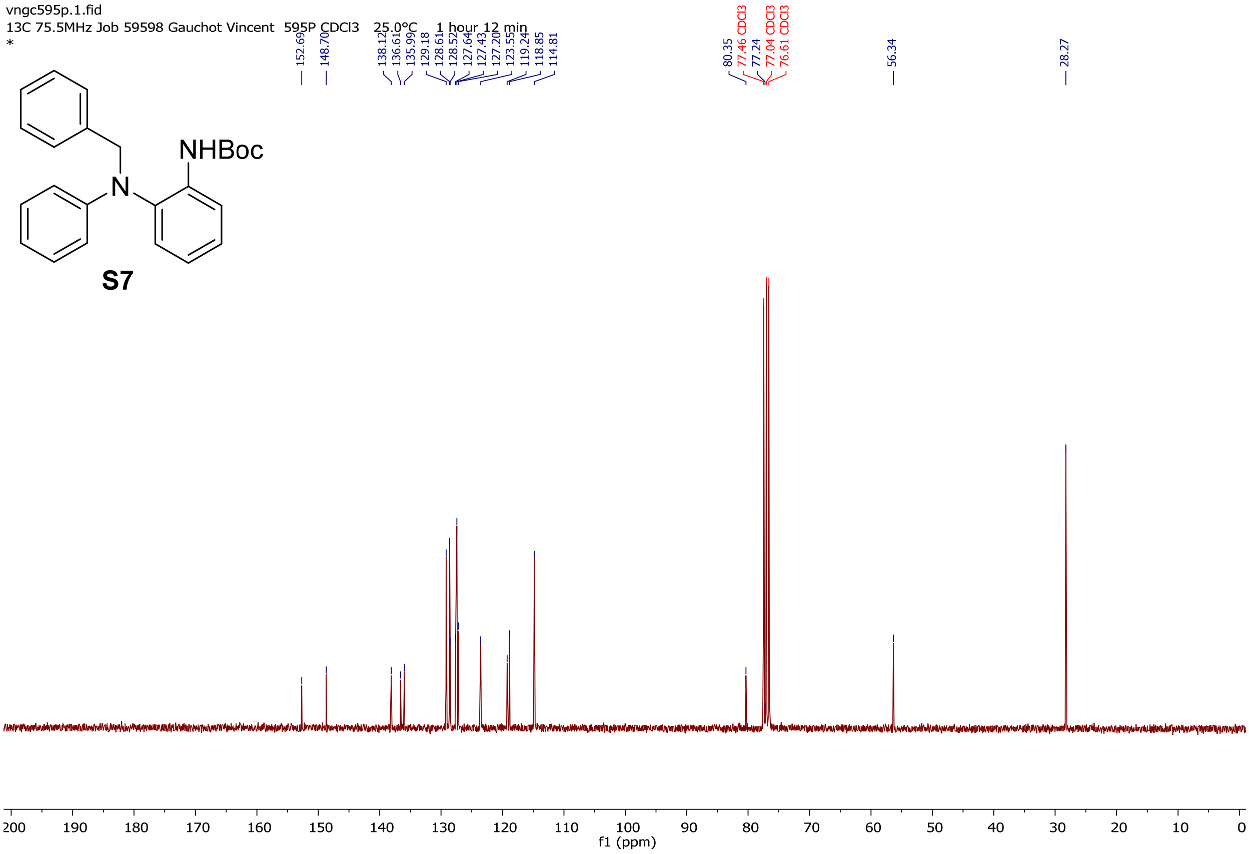
vngc595p.1.fid
13C 75.5MHz Job 59598 Gauchot Vincent 595P CDCl3 25.0ºC 1 hour 12 min
*
NHBoc
N
S7
152.69
148.70
138.12
136.61
135.99
129.18
128.61
128.52
127.64
127.43
127.20
123.55
119.24
118.85
114.81
80.35
77.46 CDCl3
77.24
77.04 CDCl3
76.61 CDCl3
56.34
28.27
f1 (ppm)

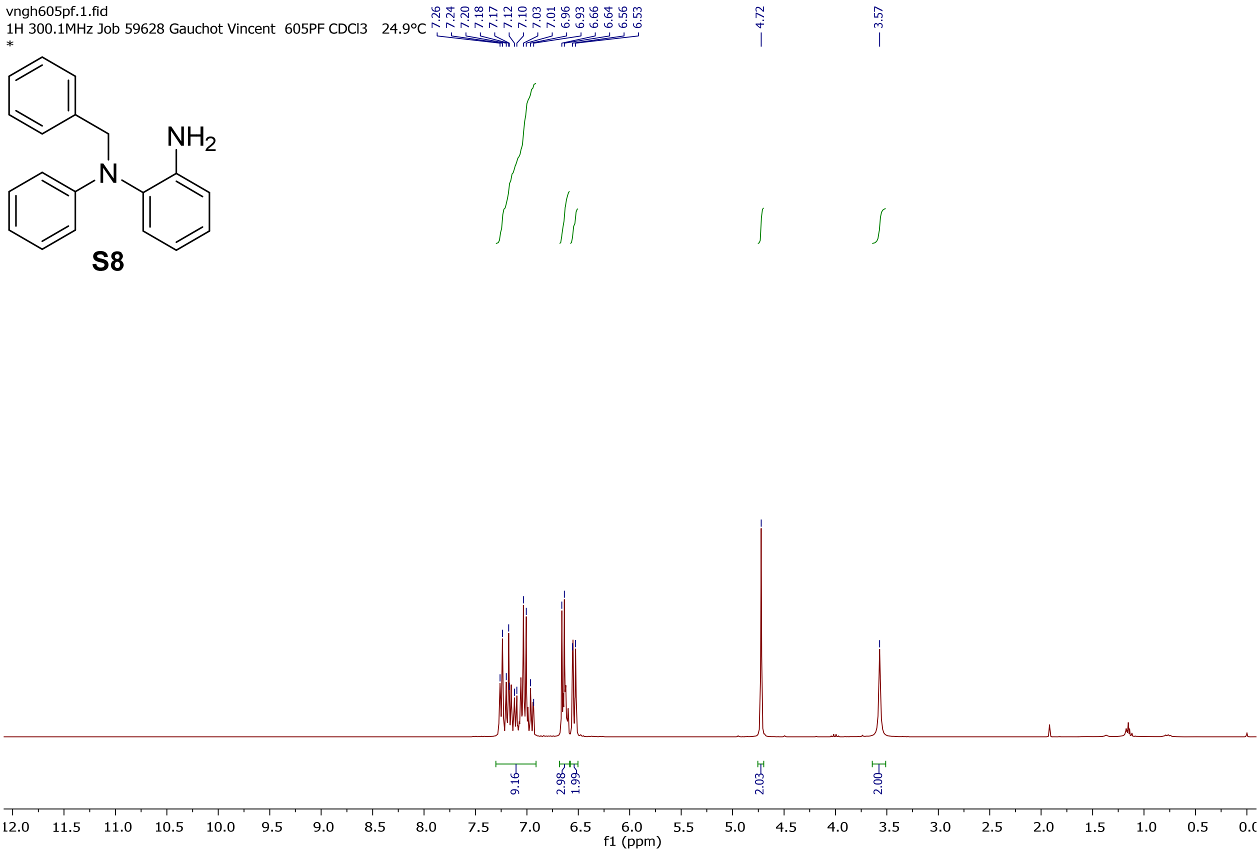
vngh605pf.1.fid
1H 300.1MHz Job 59628 Gauchot Vincent 605PF CDCl3 24.9°C
*
7.26 7.24 7.20 7.18 7.17 7.12 7.10 7.03 7.01 6.96 6.93 6.66 6.64 6.56 6.53
4.72
3.57
NH2
N
S8
9.16
2.98
1.99
2.03
2.00
12.0 11.5 11.0 10.5 10.0 9.5 9.0 8.5 8.0 7.5 7.0 6.5 6.0 5.5 5.0 4.5 4.0 3.5 3.0 2.5 2.0 1.5 1.0 0.5 0.0
f1 (ppm)

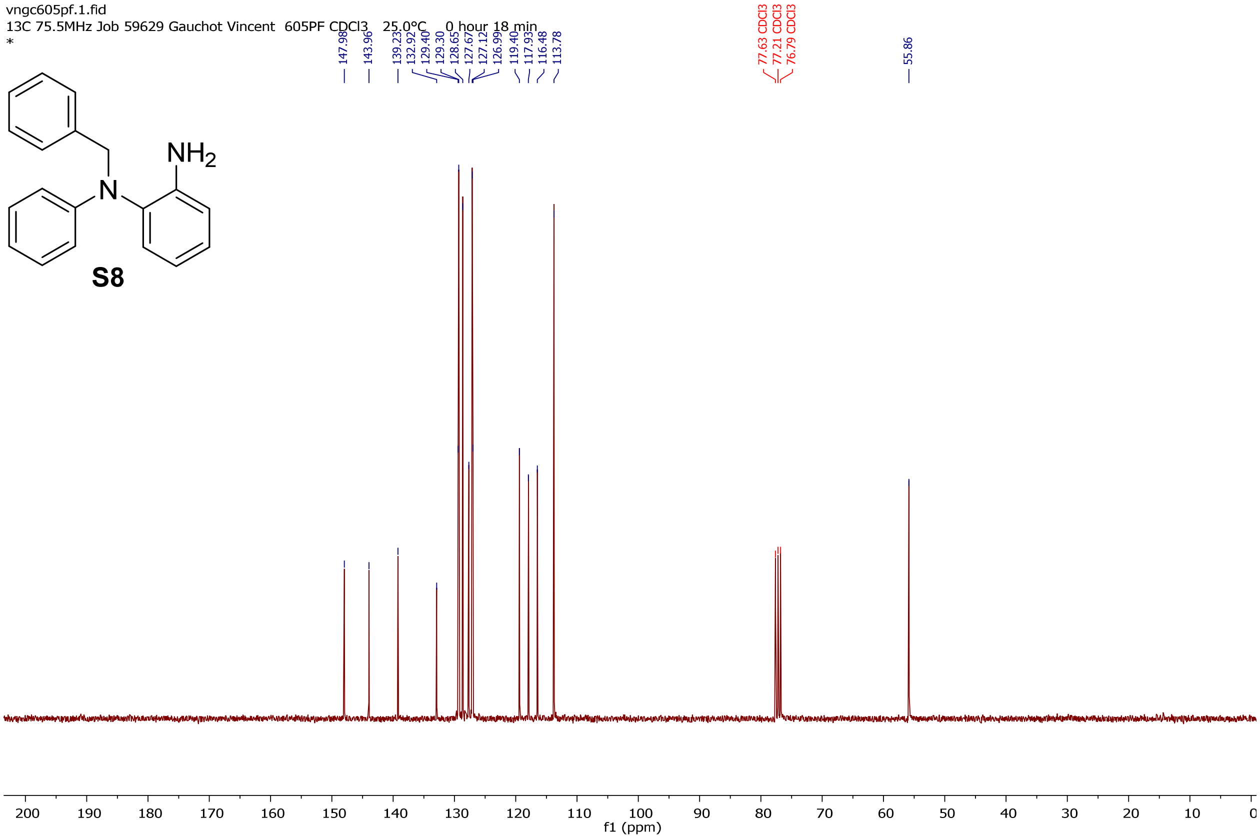
vngc605pf.1.fid
13C 75.5MHz Job 59629 Gauchot Vincent 605PF CDCl3 25.0°C 0 hour 18 min
*
147.98
143.96
139.23
132.92
129.40
129.30
128.65
127.67
127.12
126.99
119.40
117.93
116.48
113.78
77.63 CDCl3
77.21 CDCl3
76.79 CDCl3
55.86
NH2
N
S8
200 190 180 170 160 150 140 130 120 110 100 90 80 70 60 50 40 30 20 10 0
f1 (ppm)

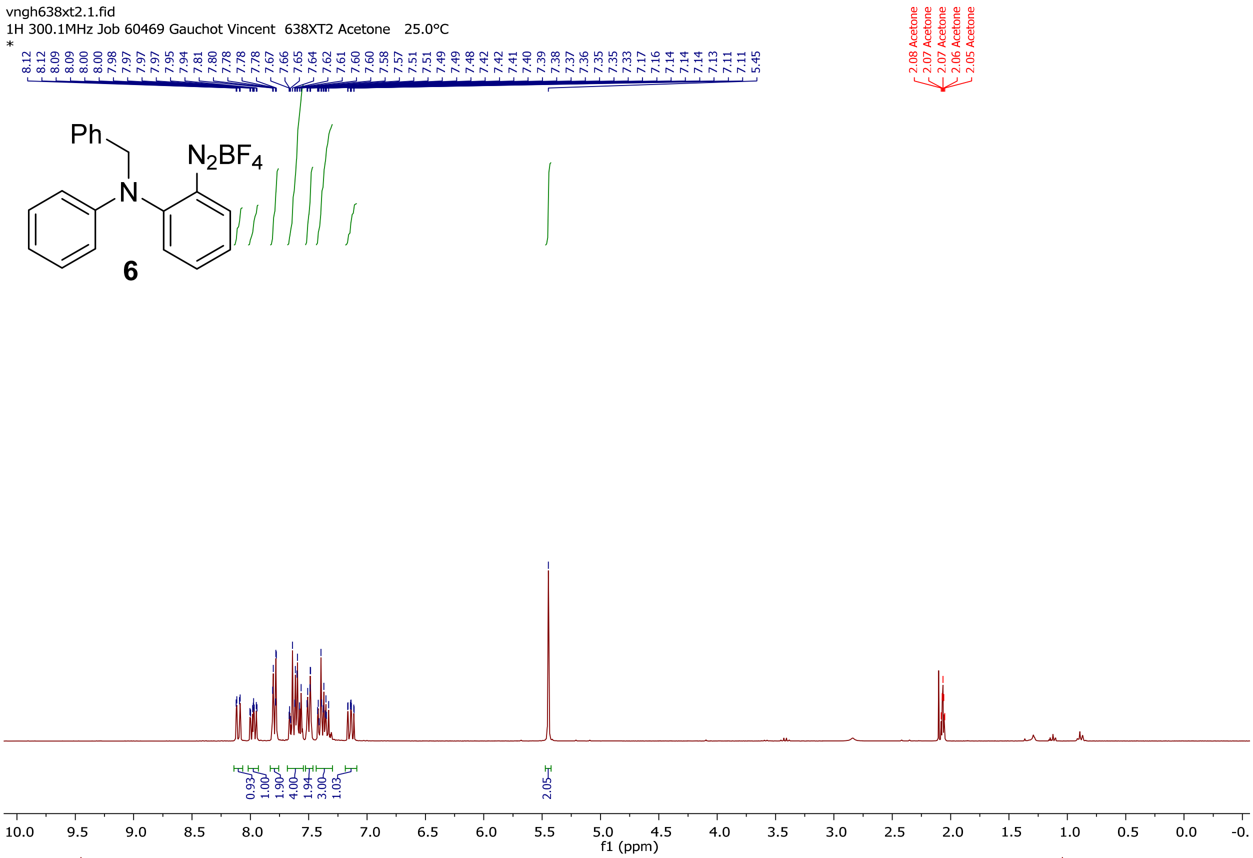
vngh638xt2.1.fid
1H 300.1MHz Job 60469 Gauchot Vincent 638XT2 Acetone 25.0°C
8.12 8.12 8.09 8.09 8.00 8.00 7.98 7.97 7.97 7.95 7.94 7.81 7.80 7.78 7.78 7.78 7.67 7.66 7.65 7.64 7.62 7.61 7.60 7.60 7.58 7.57 7.51 7.51 7.49 7.48 7.42 7.42 7.41 7.40 7.39 7.38 7.37 7.36 7.35 7.35 7.33 7.17 7.16 7.14 7.14 7.14 7.13 7.11 7.11 5.45
2.08 Acetone
2.07 Acetone
2.07 Acetone
2.06 Acetone
2.05 Acetone
Ph
N
N2BF4
6
0.93
1.00
1.90
4.00
1.94
3.00
1.03
2.05
f1 (ppm)

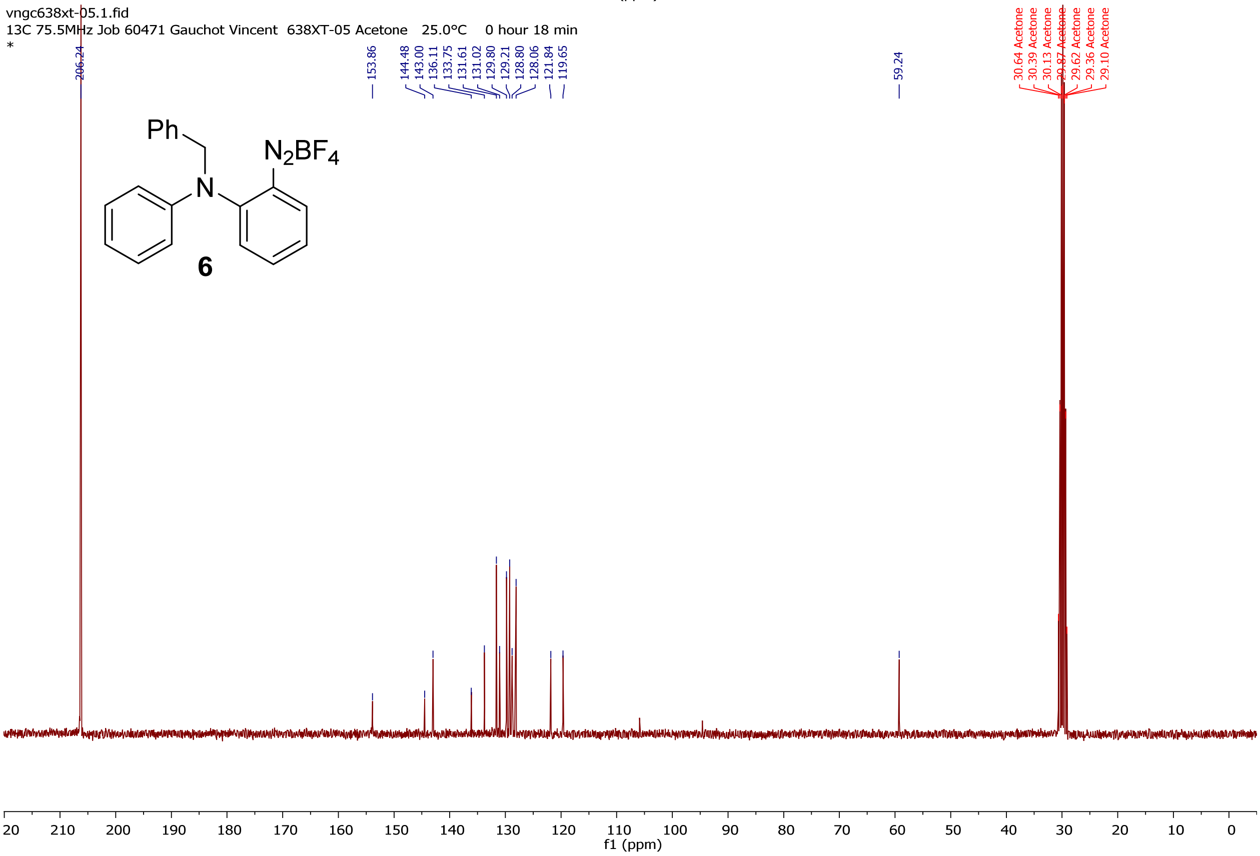
vngc638xt-05.1.fid
13C 75.5MHz Job 60471 Gauchot Vincent 638XT-05 Acetone 25.0°C 0 hour 18 min
206.24
153.86
144.48 143.00 136.11 133.75 131.61 131.02 129.80 129.21 128.80 128.06 121.84 119.65
59.24
30.64 Acetone
30.39 Acetone
30.13 Acetone
29.87 Acetone
29.62 Acetone
29.36 Acetone
29.10 Acetone
Ph
N
N2BF4
6
f1 (ppm)

vngf638xt2.1.fid
19F 282.4MHz Job 60470 Gauchot Vincent 638XT2 Acetone 25.0°C 0hours 1min
*

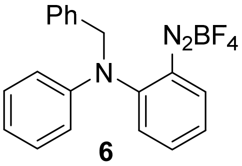

6

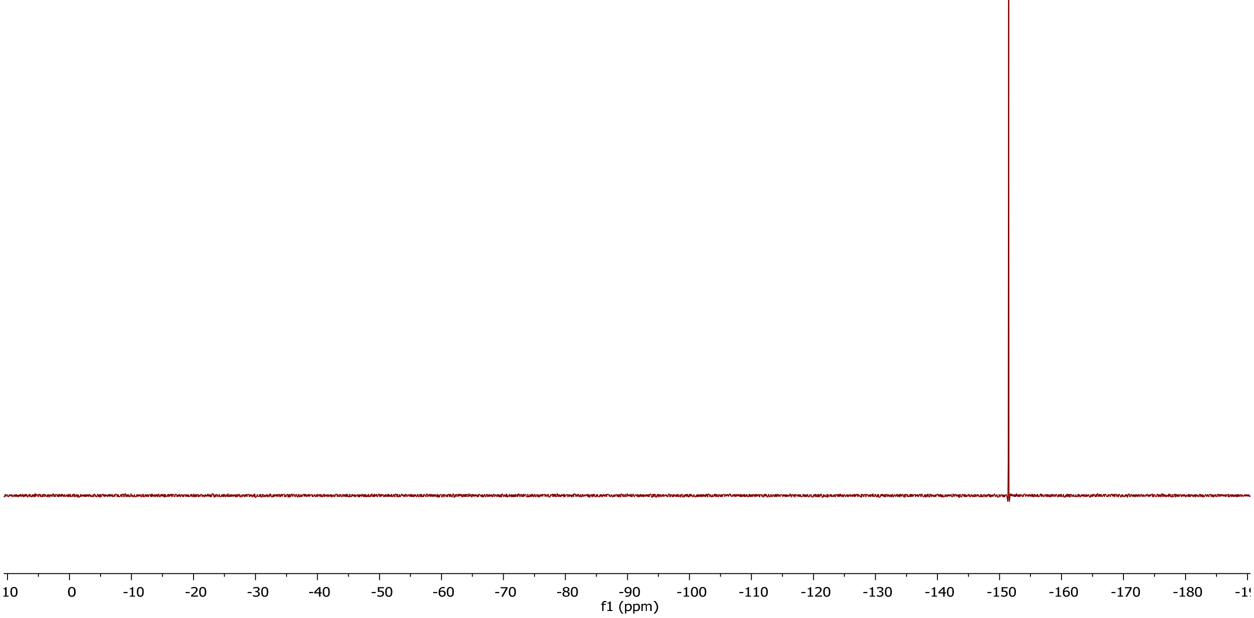

vngh479p.1.fid
1H 300.1MHz Job 58366 Gauchot Vincent 479P CDCl3 25.0°C
*

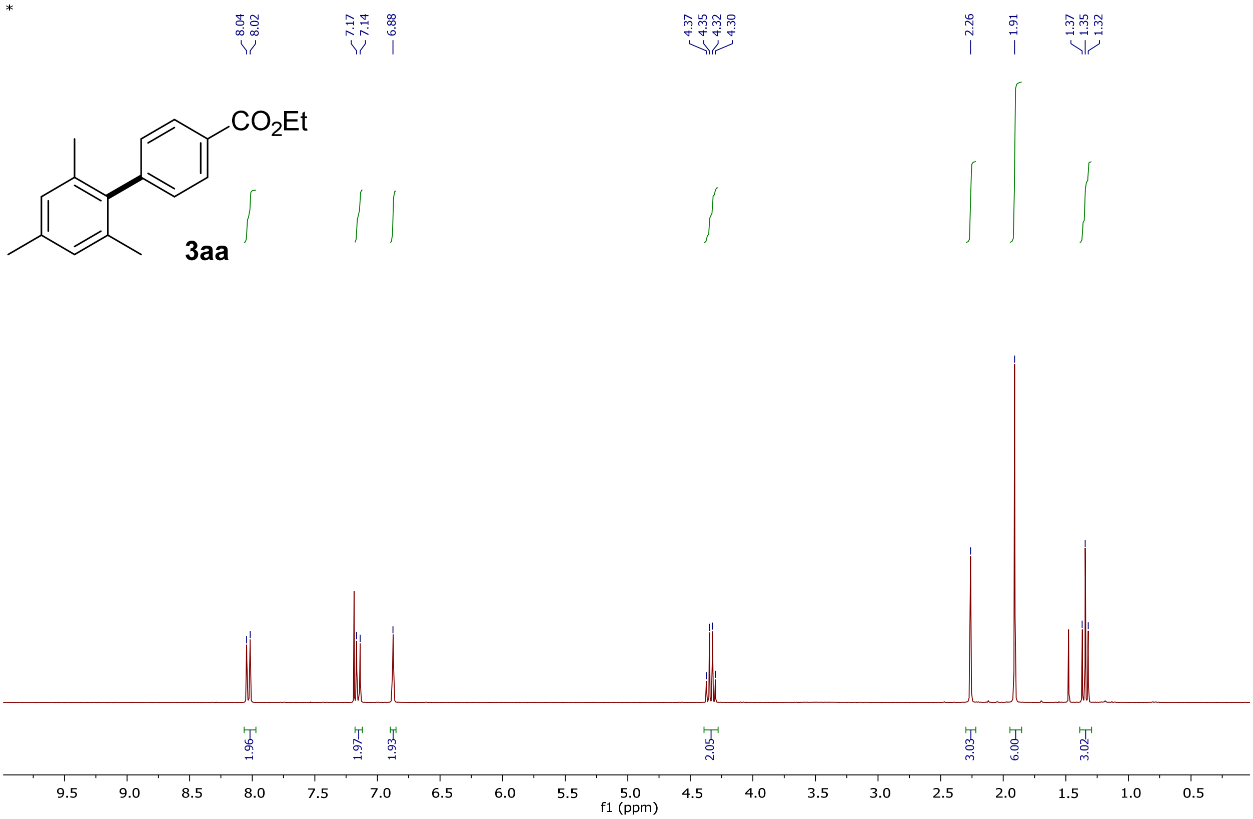

vngc479p.1.fid
13C 75.5MHz Job 58408 Gauchot Vincent 479P CDCl3 24.9°C 3 hours 1 min
*

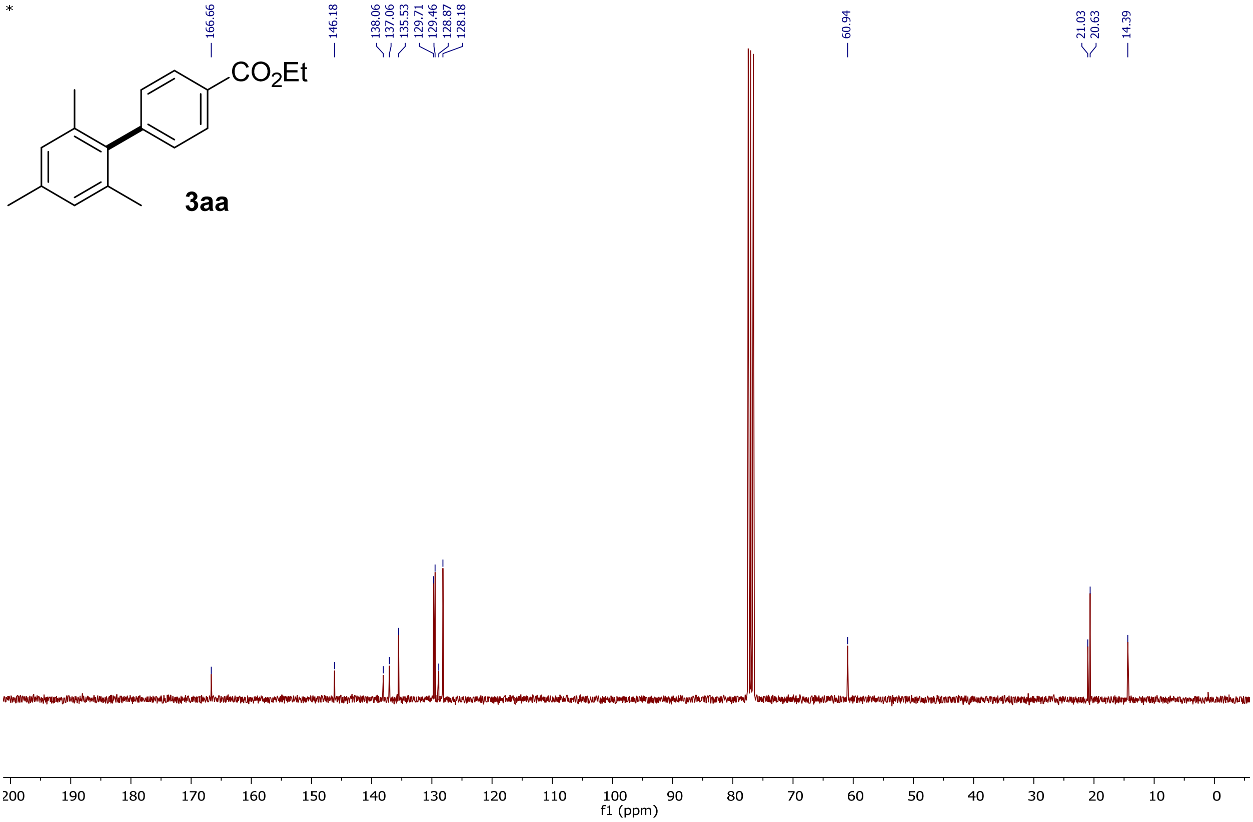

drsh57a4l04.1.fid
1H 300.1MHz Job 60605 Sutherland Daniel R 57A4L04 CDCl3 25.0°C
*

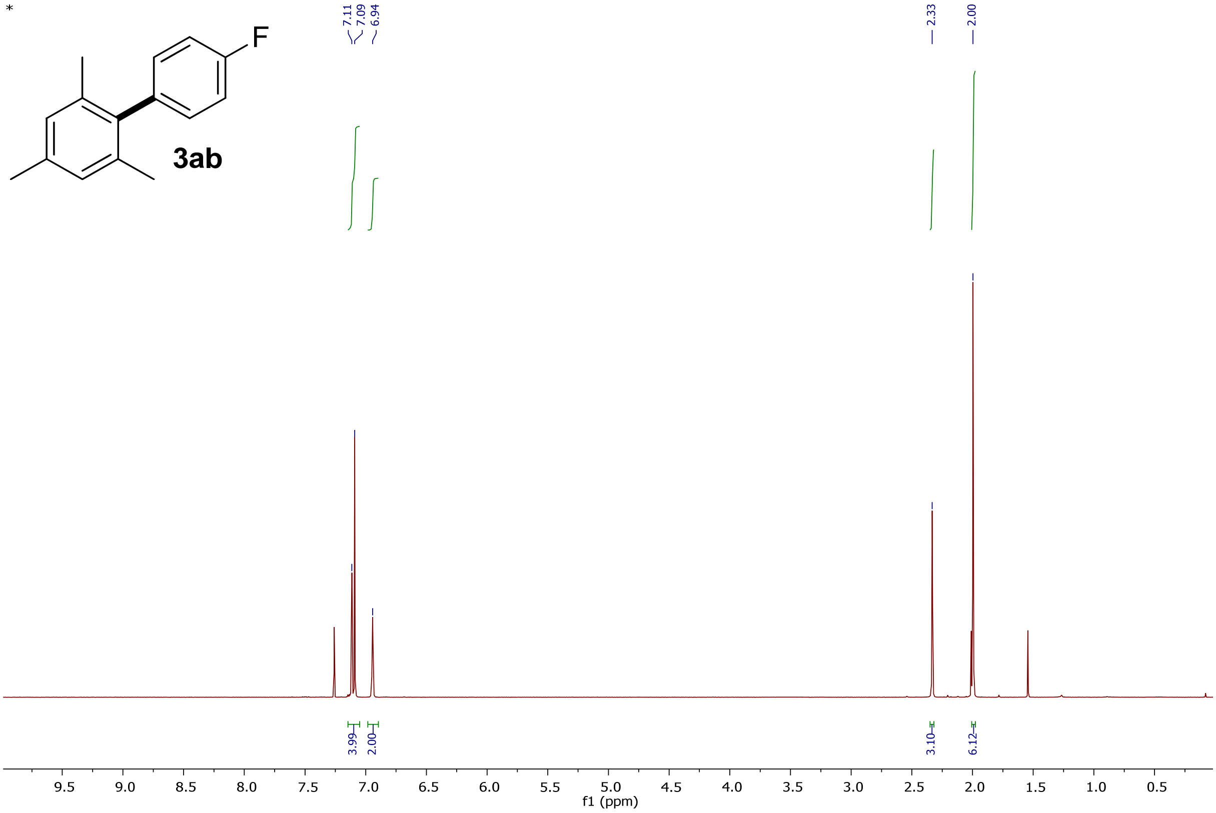

drsc57a4l02.1.fid
13C 75.5MHz Job 58822 Sutherland Daniel R 57A4L02 CDCl3 25.0°C 1 hour 48 min
*

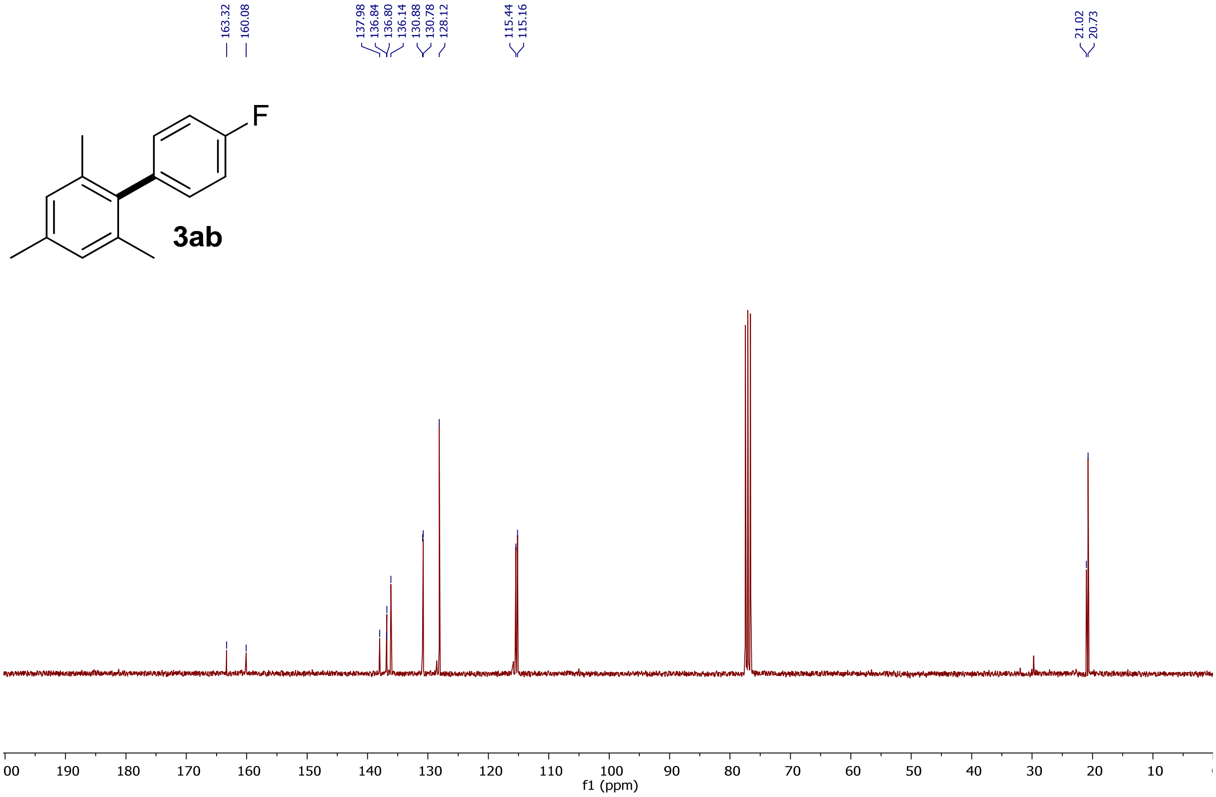

drsh57a4b02.1.fid
1H 300.1MHz Job 58407 Sutherland Daniel R 57A4B02 CDCl3 25.1°C
*

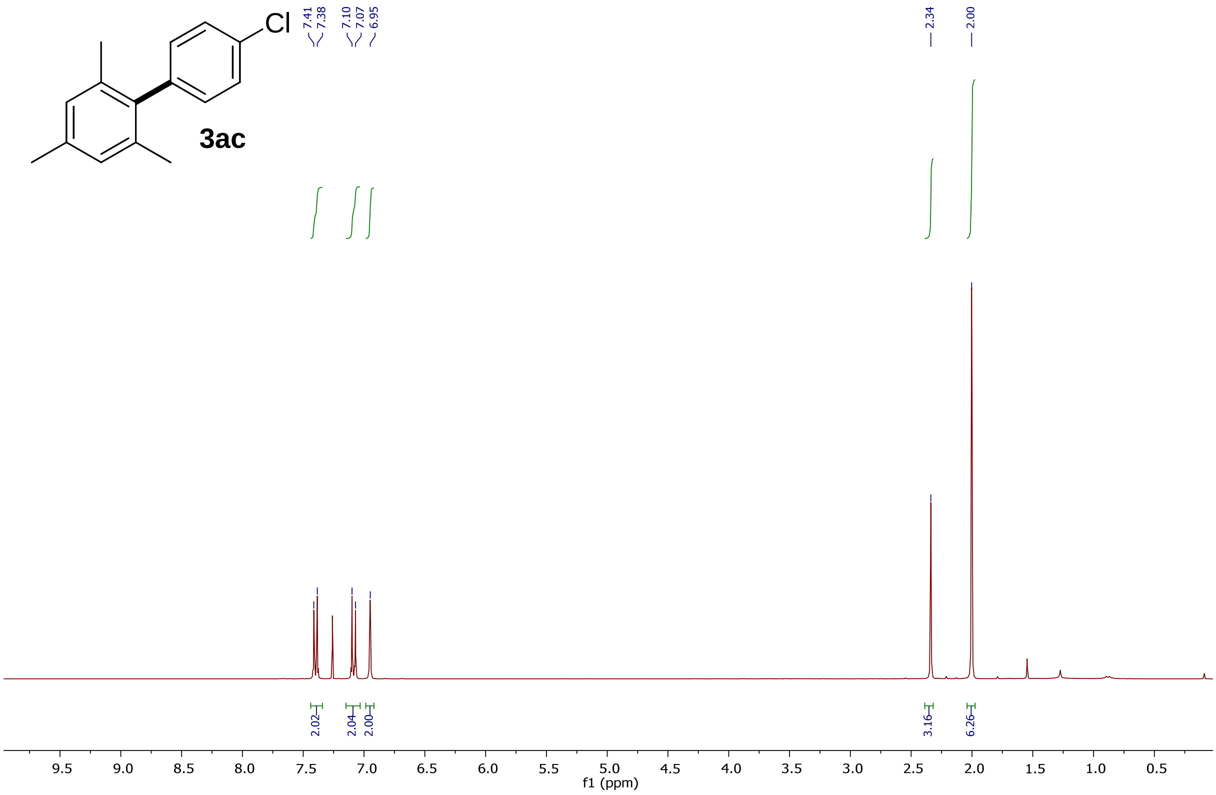

drsc57a4b02.1.fid
13C 75.5MHz Job 58410 Sutherland Daniel R 57A4B02 CDCl3 25.1°C 1 hour 48 min
*

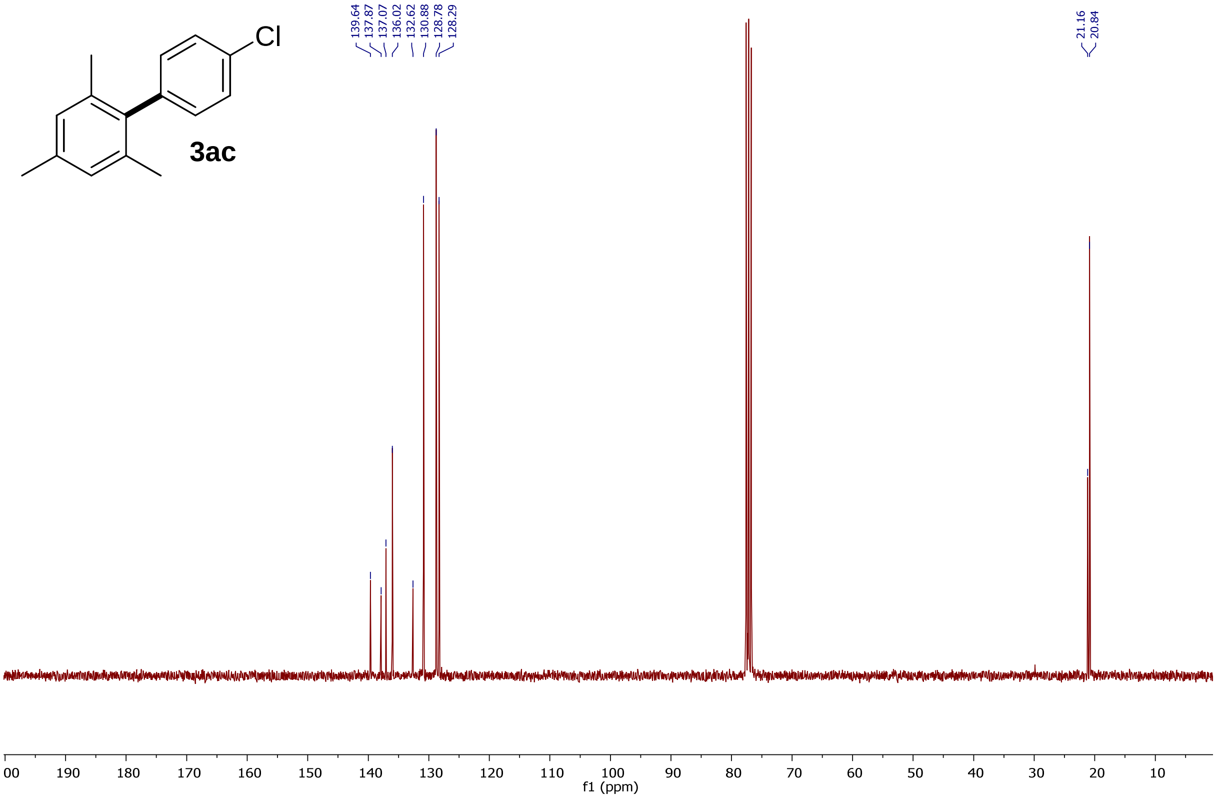

drsh57a4e02.1.fid
1H 300.1MHz Job 58422 Sutherland Daniel R 57A4E02 CDCl3 24.9°C
*

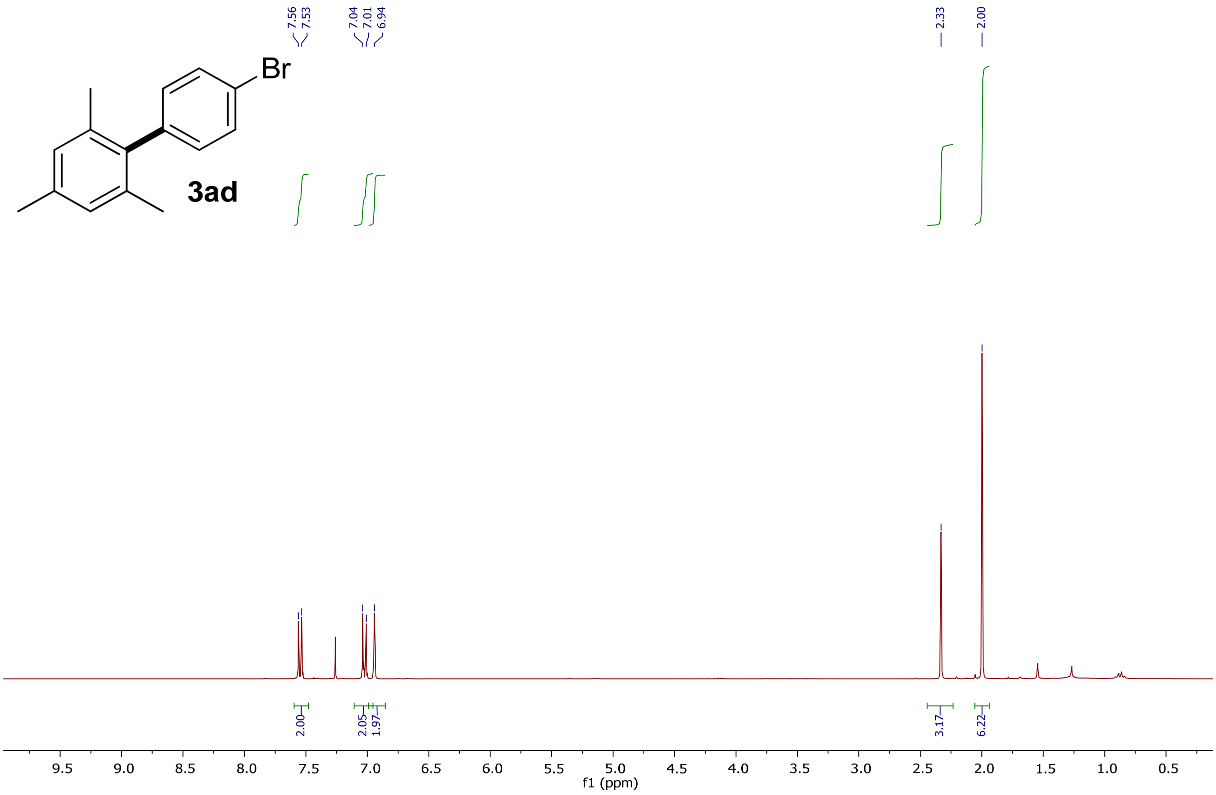

drsc57a4e02.1.fid
13C 75.5MHz Job 58442 Sutherland Daniel R 57A4E02 CDCl3 25.0°C 2 hours 25 min
*

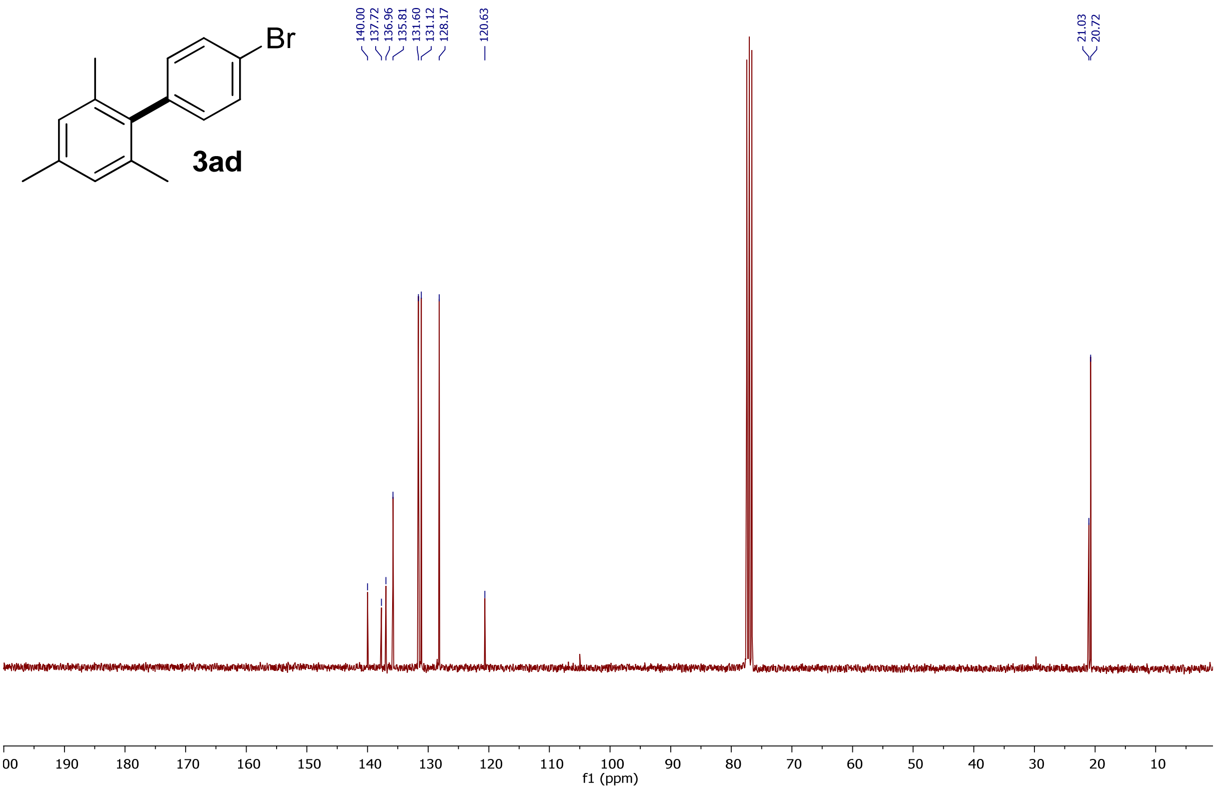

drsh57a4f02.1.fid
1H 300.1MHz Job 58440 Sutherland Daniel R 57A4F02 CDCl3   25.0°C
*

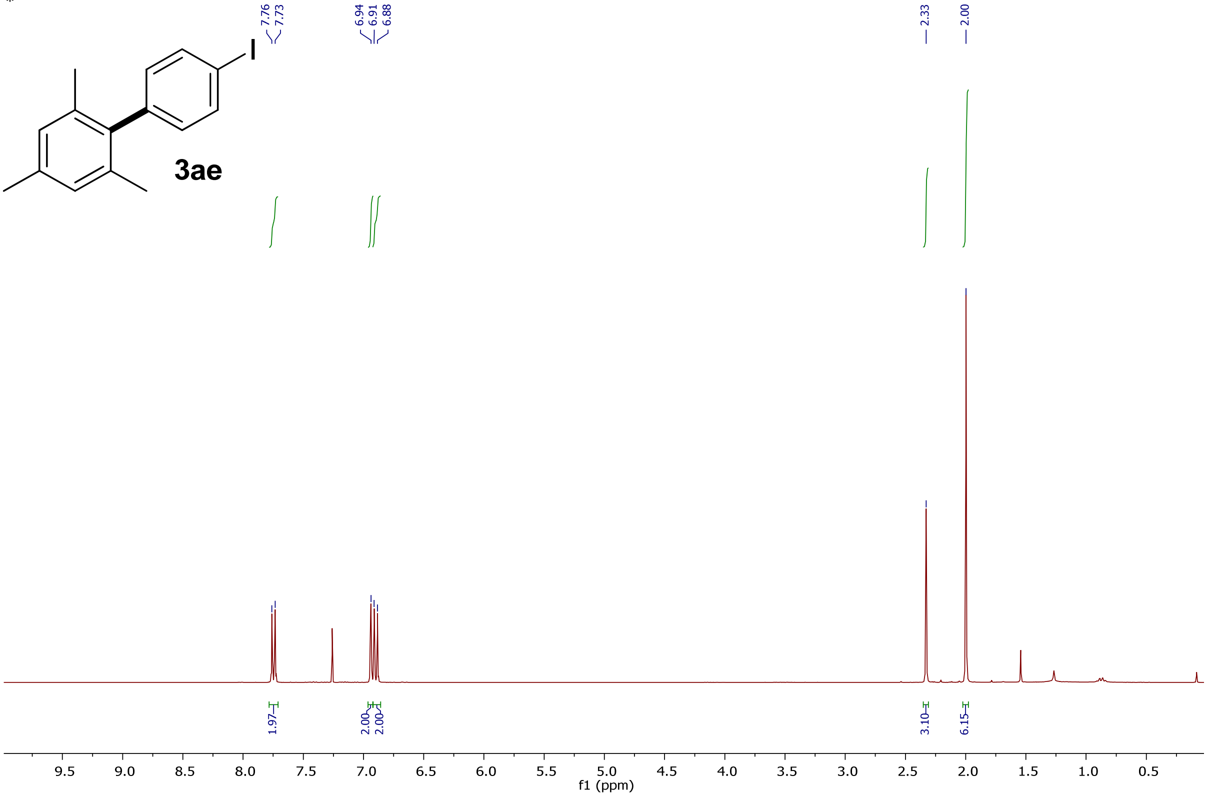

drsc57a4f02.1.fid
13C 75.5MHz Job 58443 Sutherland Daniel R 57A4F02 CDCl3   25.0°C   3 hours 1 min
*

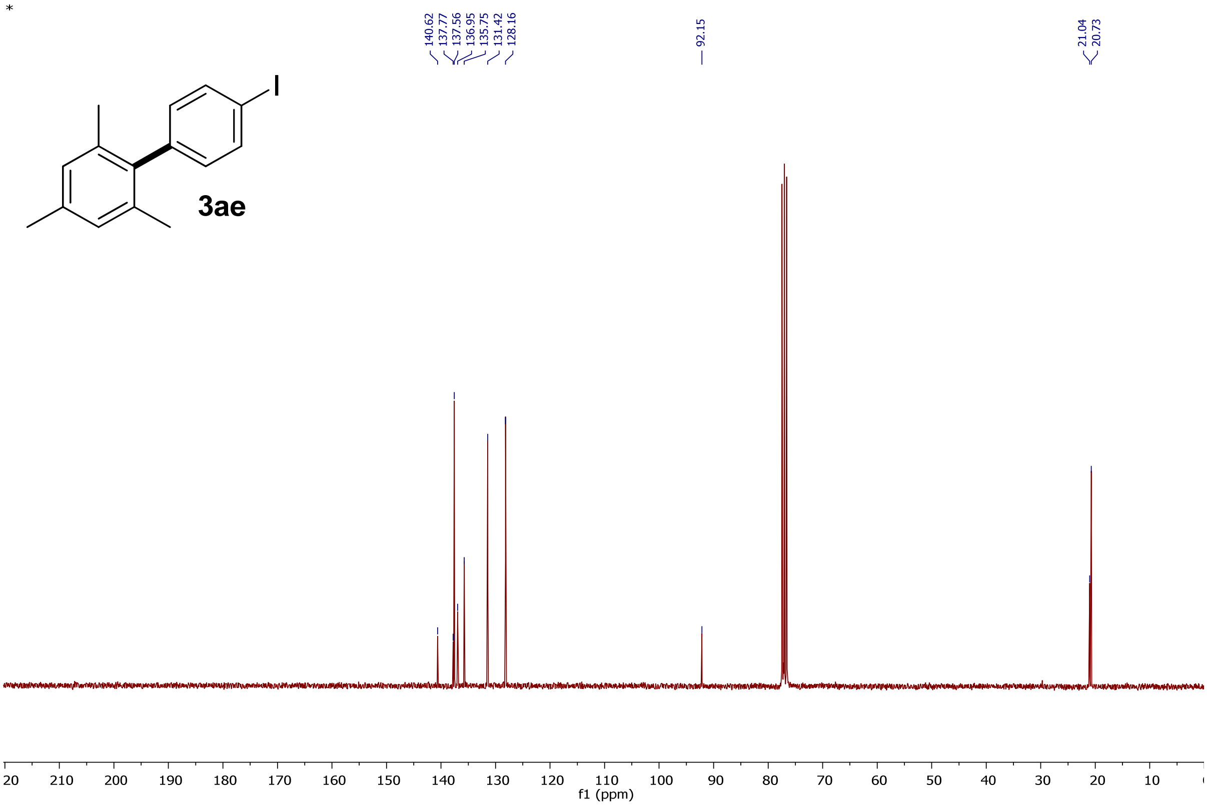

drsh57a7d02.1.fid
1H 300.1MHz Job 58774 Sutherland Daniel R 57A7D02 CDCl3 24.9°C
*

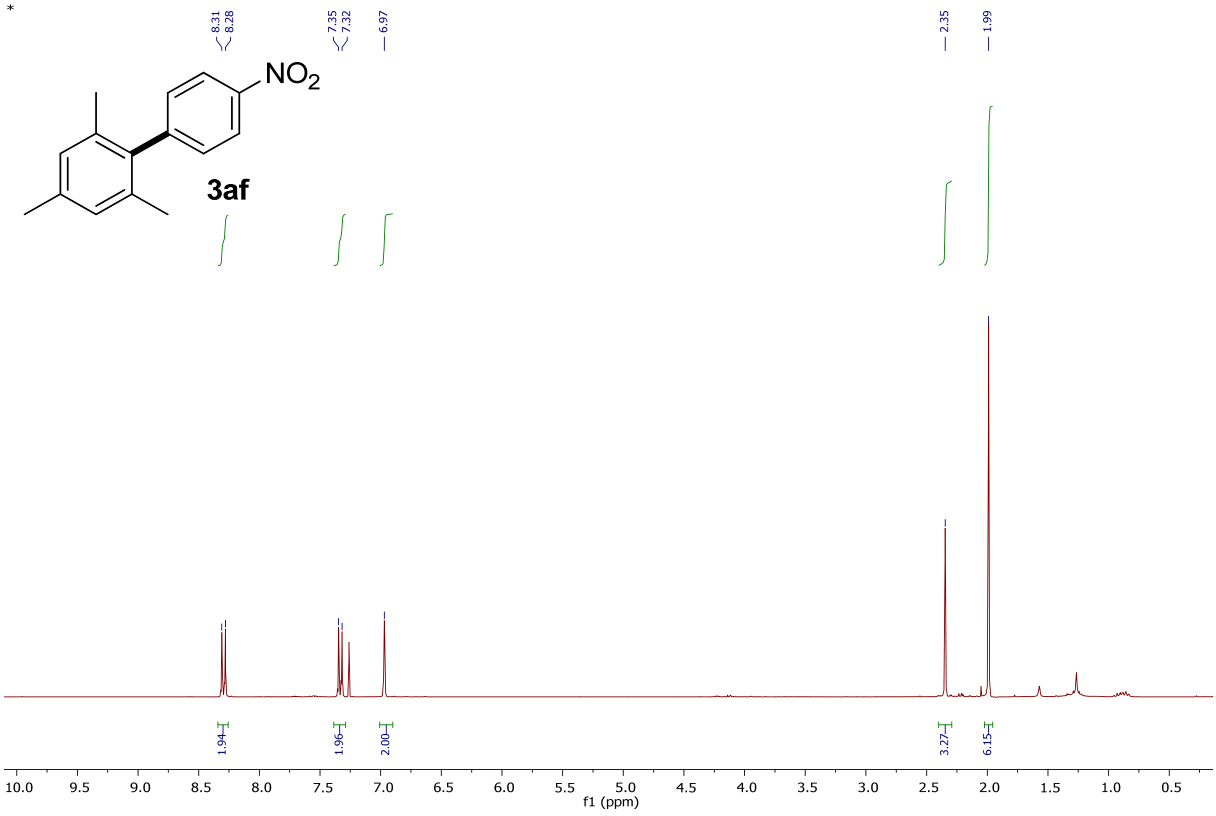

drsc57a4d02.1.fid
13C 75.5MHz Job 58547 Sutherland Daniel R 57A4D02 CDCl3 25.0°C 3 hours 1 min
*

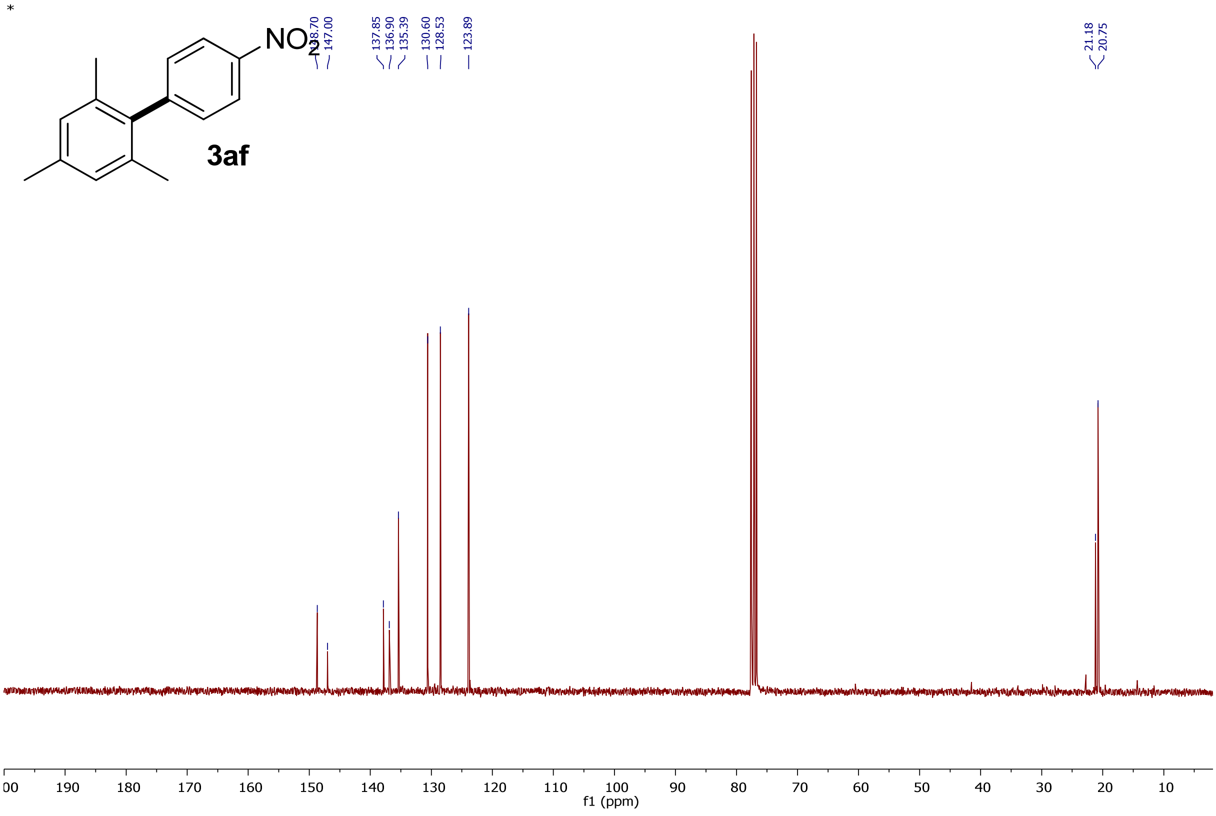

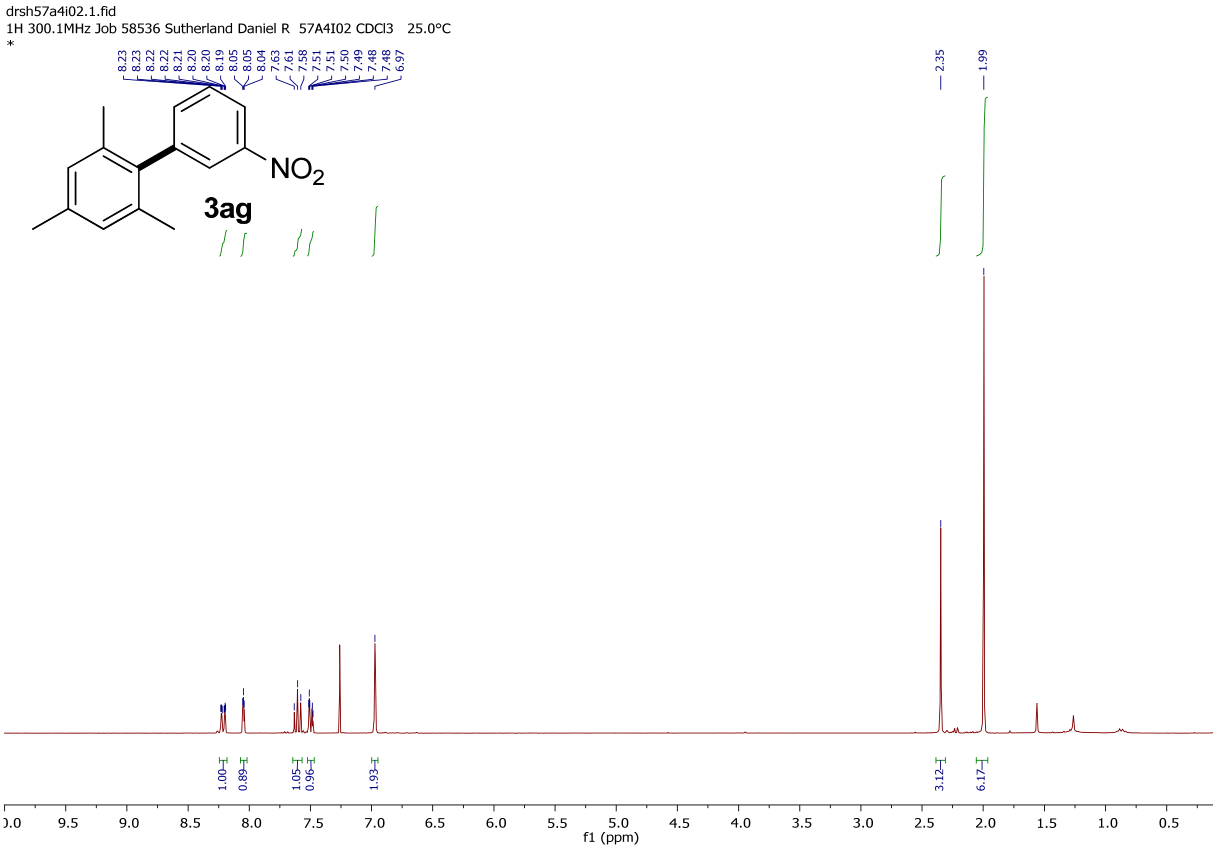
drsh57a4i02.1.fid
1H 300.1MHz Job 58536 Sutherland Daniel R 57A4I02 CDCl3 25.0°C
*
8.23 8.23 8.22 8.22 8.21 8.20 8.20 8.19 8.05 8.05 8.04 7.63 7.61 7.58 7.51 7.51 7.50 7.49 7.48 7.48 6.97
2.35
1.99
NO_2
3ag
1.00 0.89 1.05 0.96 1.93 3.12 6.17
f1 (ppm)

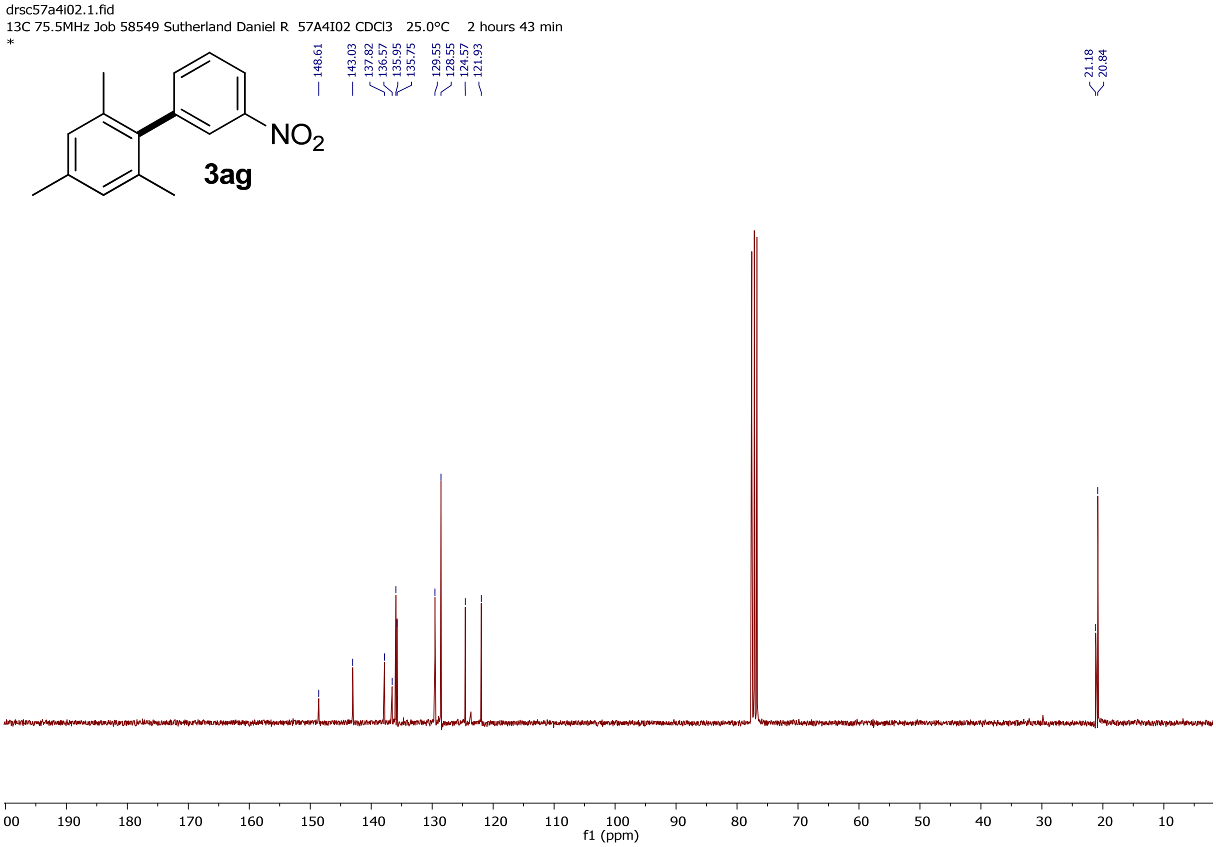
drsc57a4i02.1.fid
13C 75.5MHz Job 58549 Sutherland Daniel R 57A4I02 CDCl3 25.0°C 2 hours 43 min
*
148.61
143.03
137.82 136.57 135.95 135.75
129.55 128.55 124.57 121.93
21.18 20.84
NO_2
3ag
f1 (ppm)

drsh57a4j02.1.fid
1H 300.1MHz Job 58504 Sutherland Daniel R 57A4J02 CDCl3 25.0°C
*

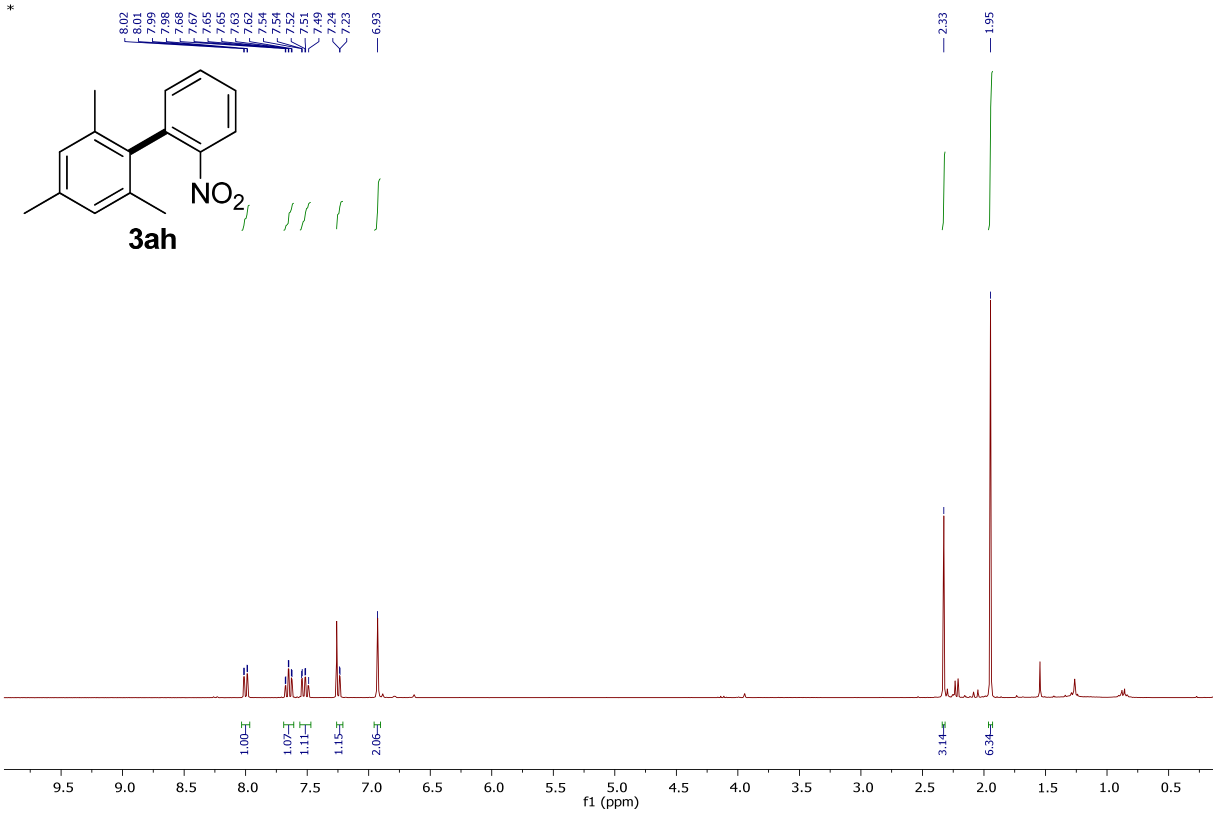

drsc57a4j02.1.fid
13C 75.5MHz Job 58709 Sutherland Daniel R 57A4J02 CDCl3 25.0°C 3 hours 1 min
*

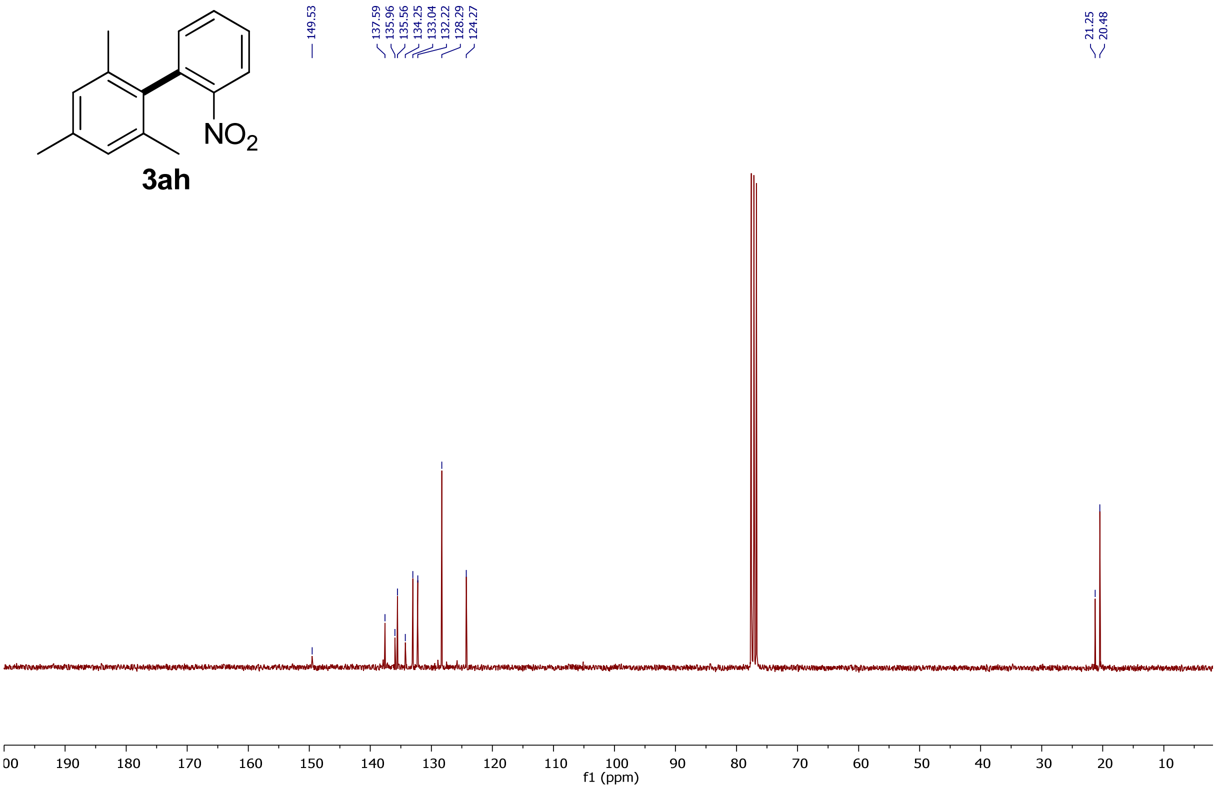

drsh57a4n02.1.fid
1H 300.1MHz Job 58621 Sutherland Daniel R 57A4N02 CDCl3 25.0°C
*

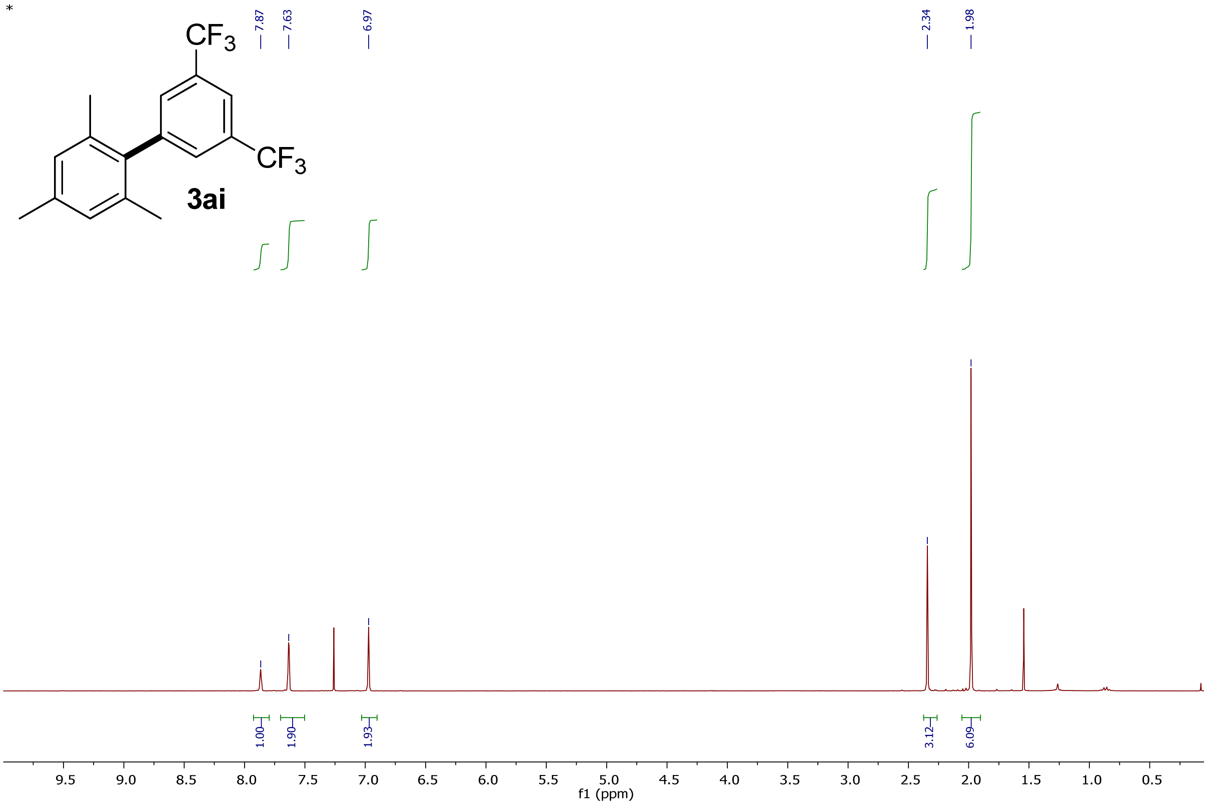

drsc57a4n02.1.fid
13C 75.5MHz Job 58707 Sutherland Daniel R 57A4N02 CDCl3 25.0°C 3 hours 1 min
*

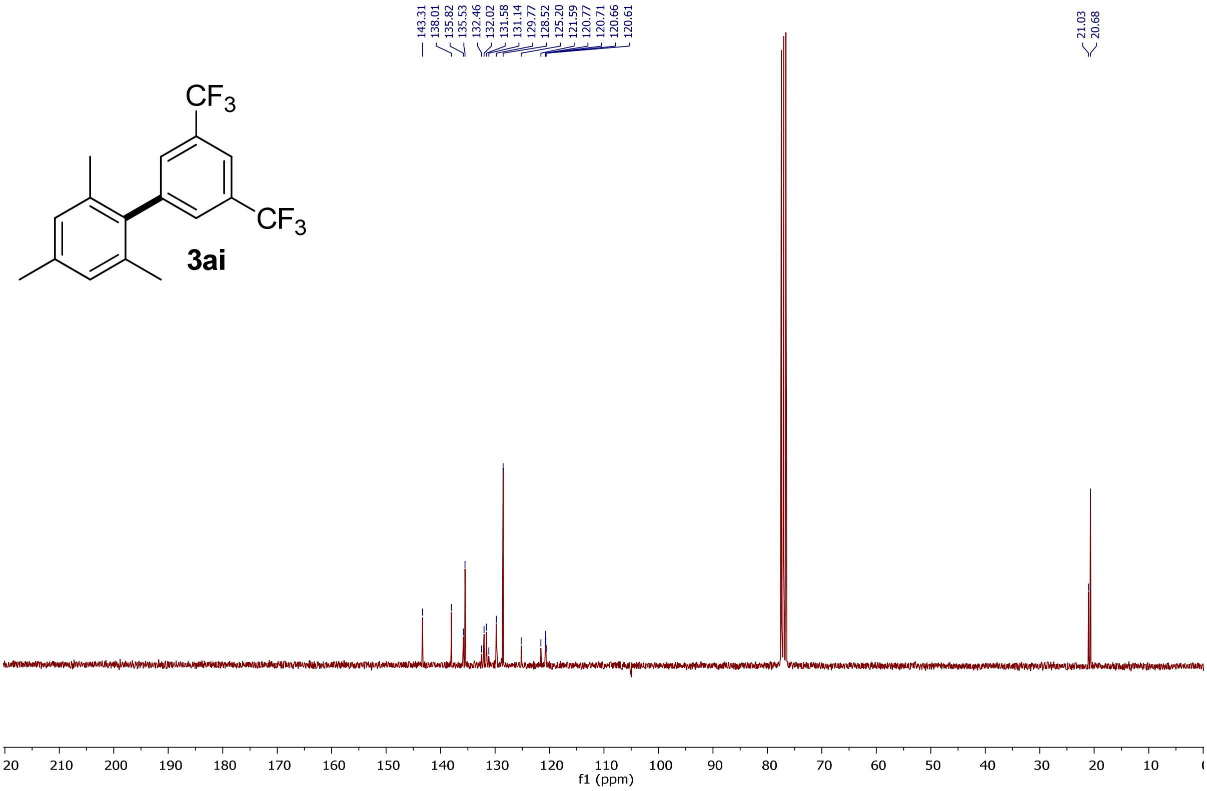

drsh57a4m02.1.fid
1H 300.1MHz Job 58730 Sutherland Daniel R 57A4M02 CDCl3 25.1°C

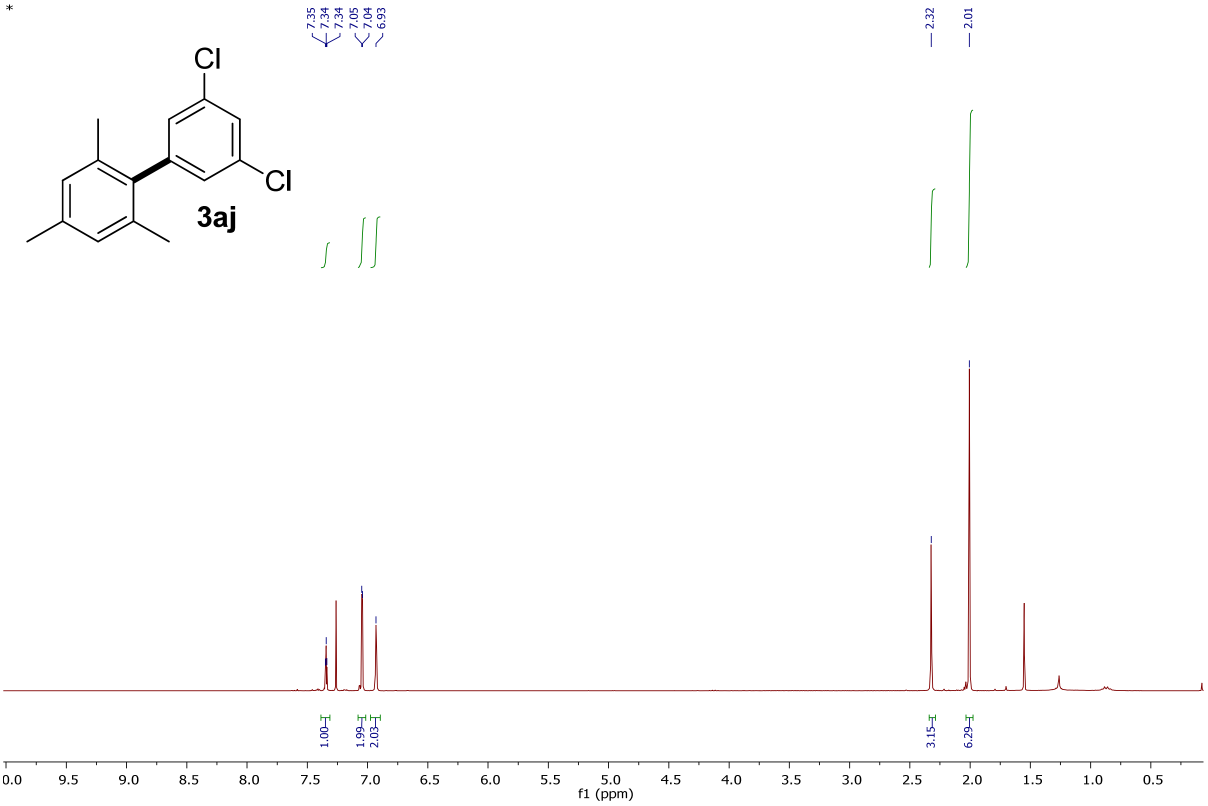

drsc57a4m02.1.fid
13C 75.5MHz Job 58788 Sutherland Daniel R 57A4M02 CDCl3 25.0°C 3 hours 1 min

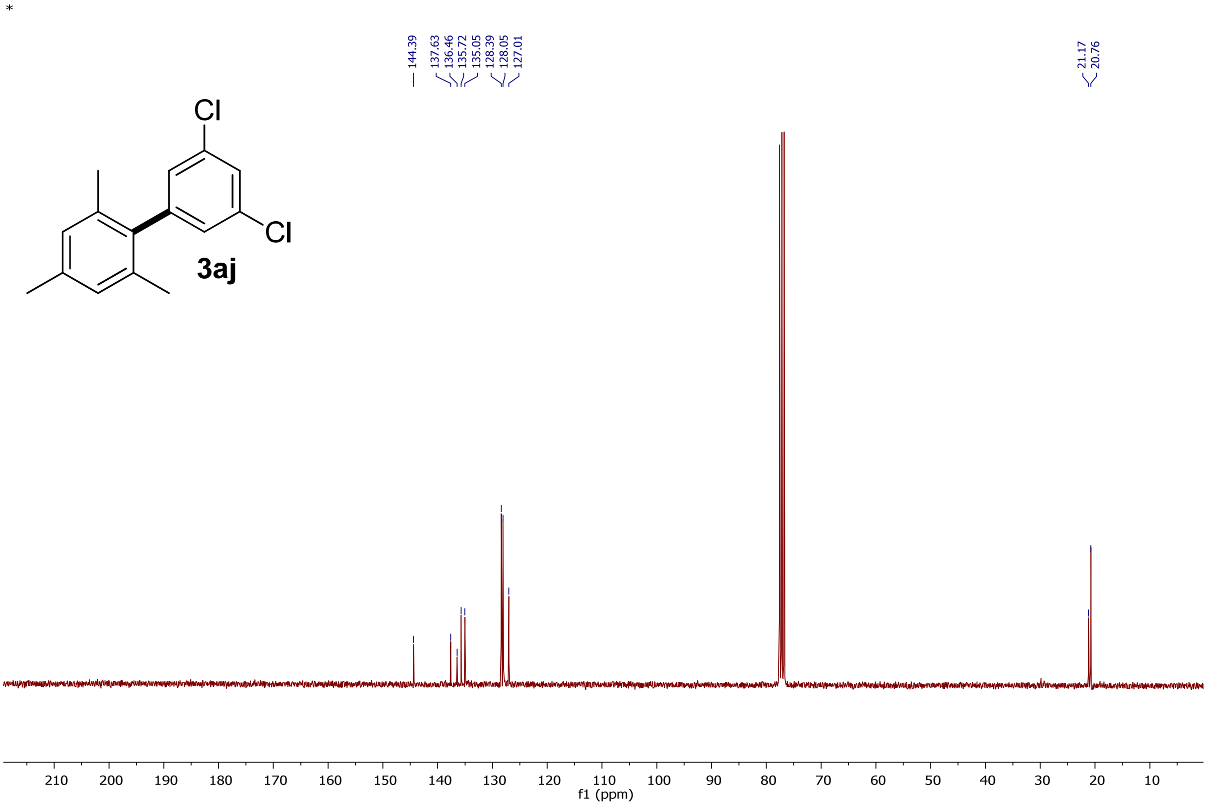

drsh57a4k04.1.fid
1H 300.1MHz Job 58692 Sutherland Daniel R 57A4K04 CDCl3 25.0°C
*

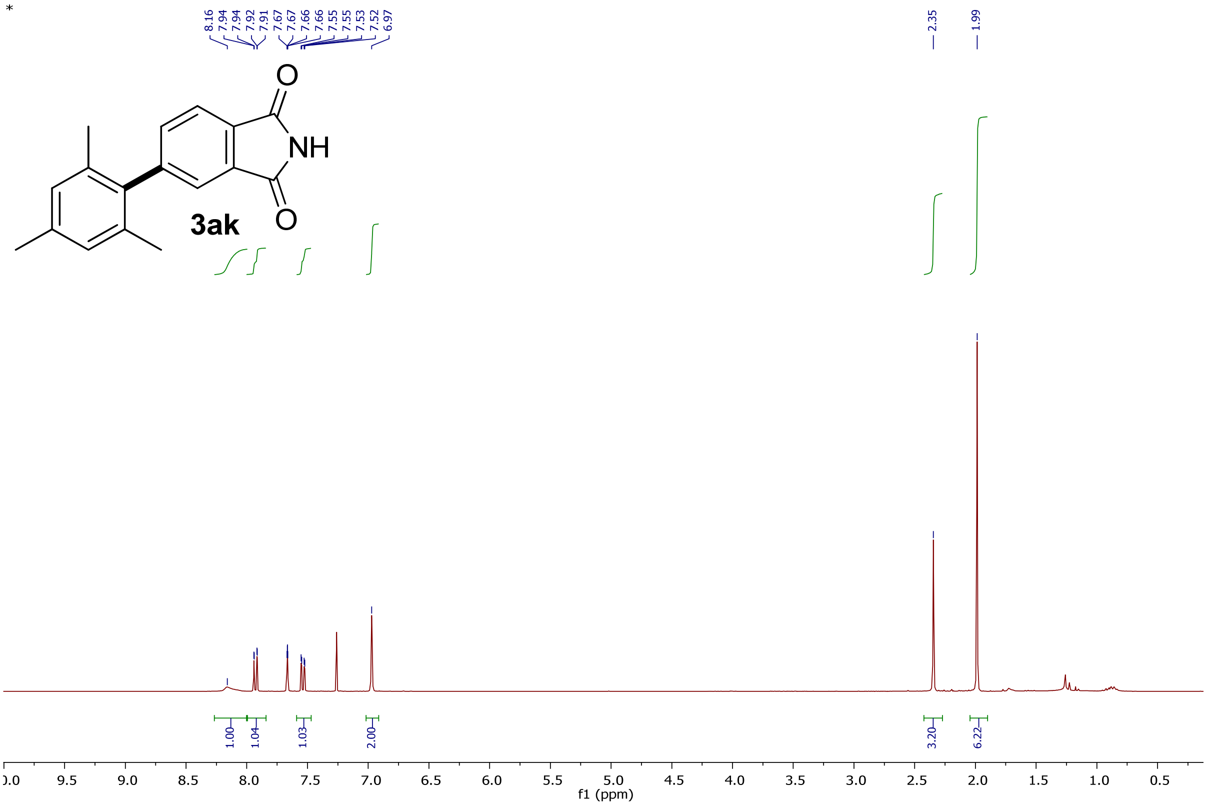

drsc57a4k04.1.fid
13C 75.5MHz Job 58710 Sutherland Daniel R 57A4K04 CDCl3 25.0°C 1 hour 12 min
*

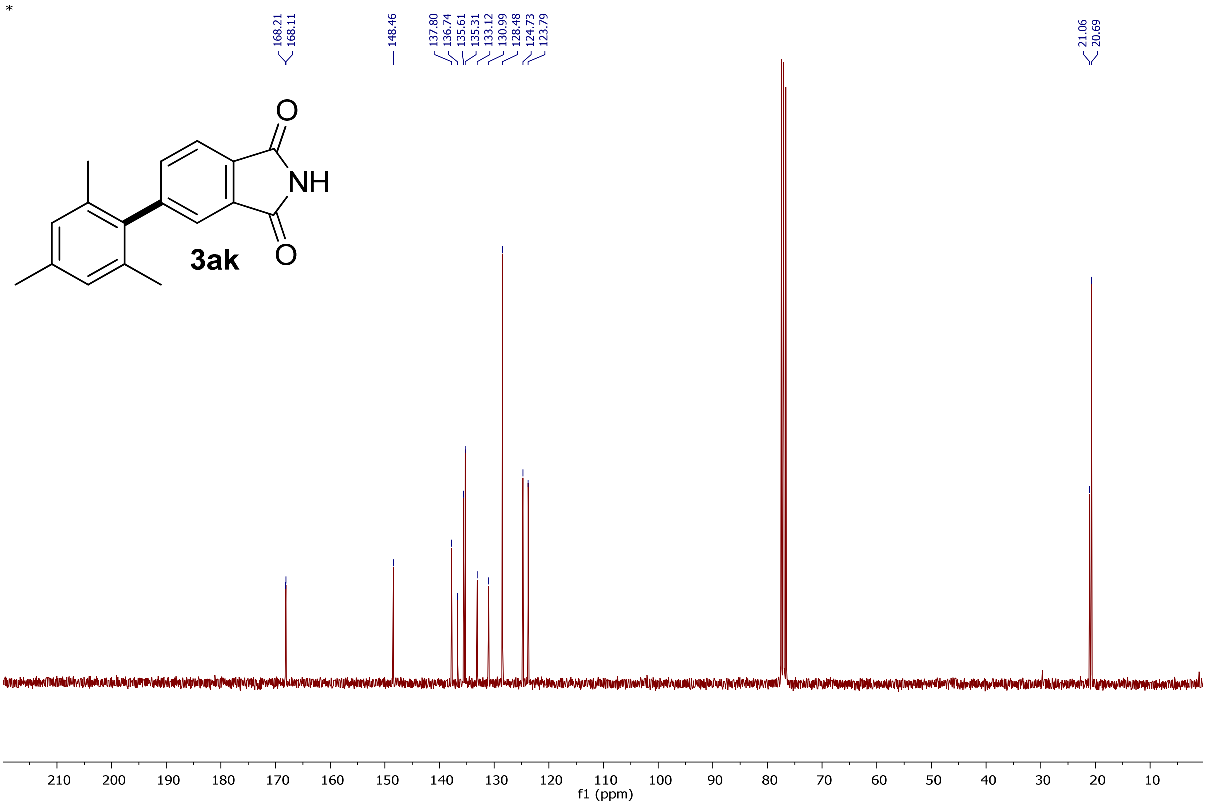

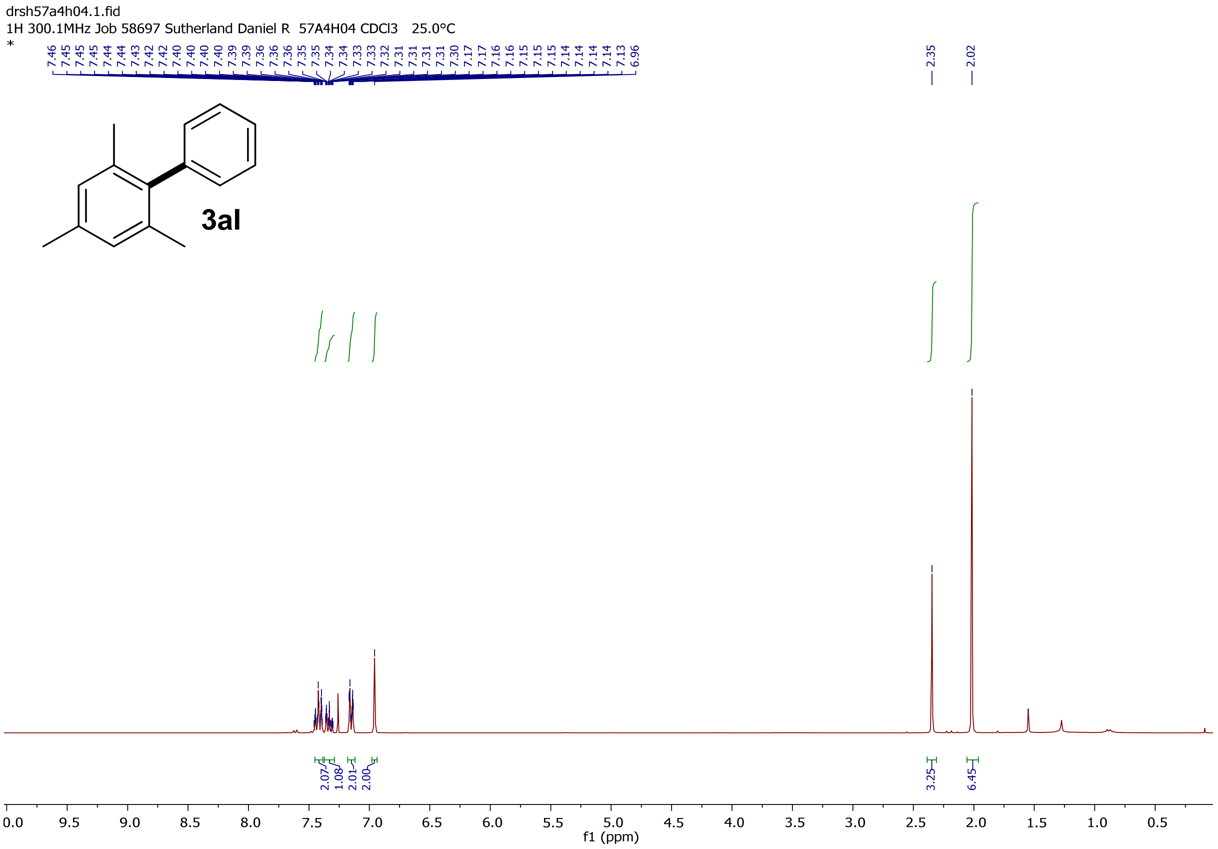
drsh57a4h04.1.fid
1H 300.1MHz Job 58697 Sutherland Daniel R 57A4H04 CDCl3 25.0°C
7.46 7.45 7.45 7.44 7.44 7.43 7.42 7.42 7.40 7.40 7.40 7.39 7.39 7.36 7.36 7.35 7.35 7.34 7.34 7.33 7.33 7.32 7.31 7.31 7.31 7.30 7.17 7.16 7.15 7.15 7.14 7.14 7.14 7.13 6.96
2.35
2.02
3al
2.07
1.08
2.01
2.00
3.25
6.45
f1 (ppm)

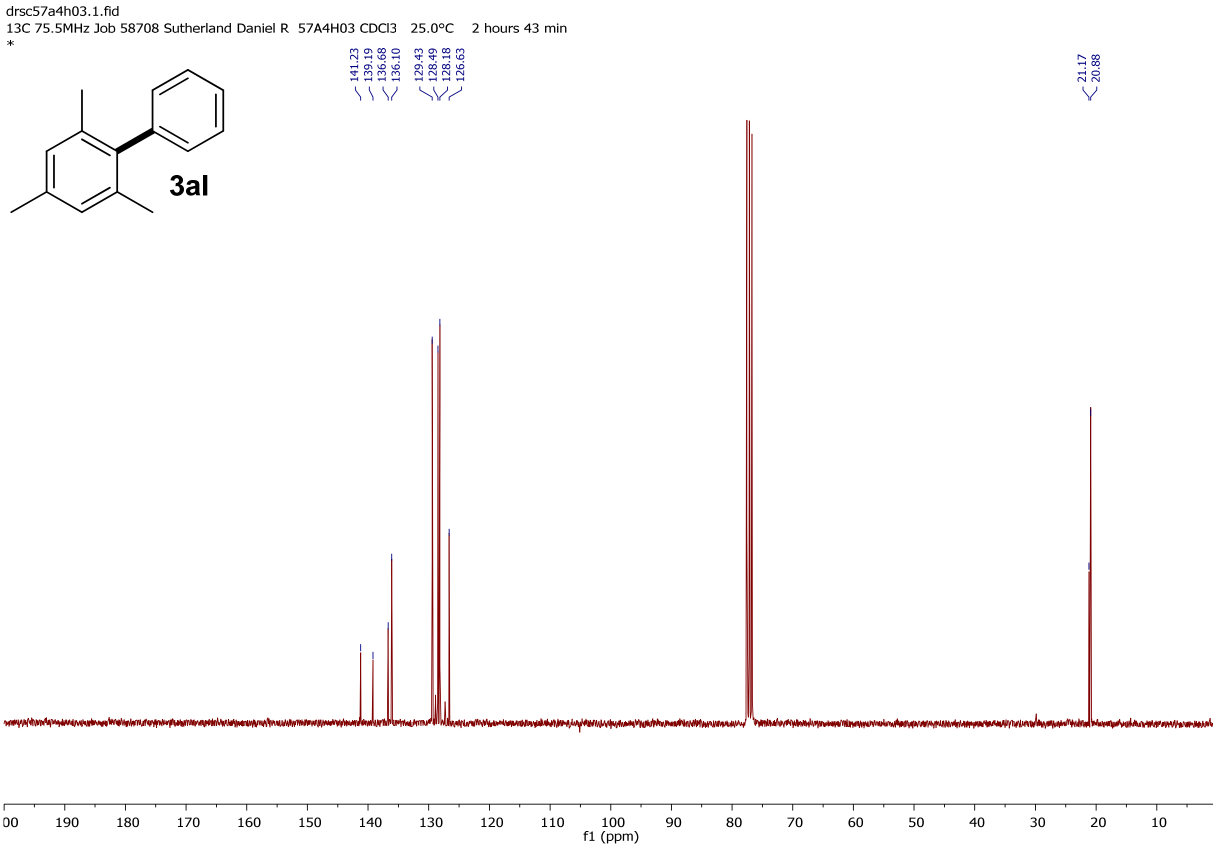
drsc57a4h03.1.fid
13C 75.5MHz Job 58708 Sutherland Daniel R 57A4H03 CDCl3 25.0°C 2 hours 43 min
141.23 139.19 136.68 136.10 129.43 128.49 128.18 126.63
21.17 20.88
3al
f1 (ppm)

drsh57a4c02.1.fid
1H 300.1MHz Job 58400 Sutherland Daniel R 57A4C02 CDCl3 24.9°C
*

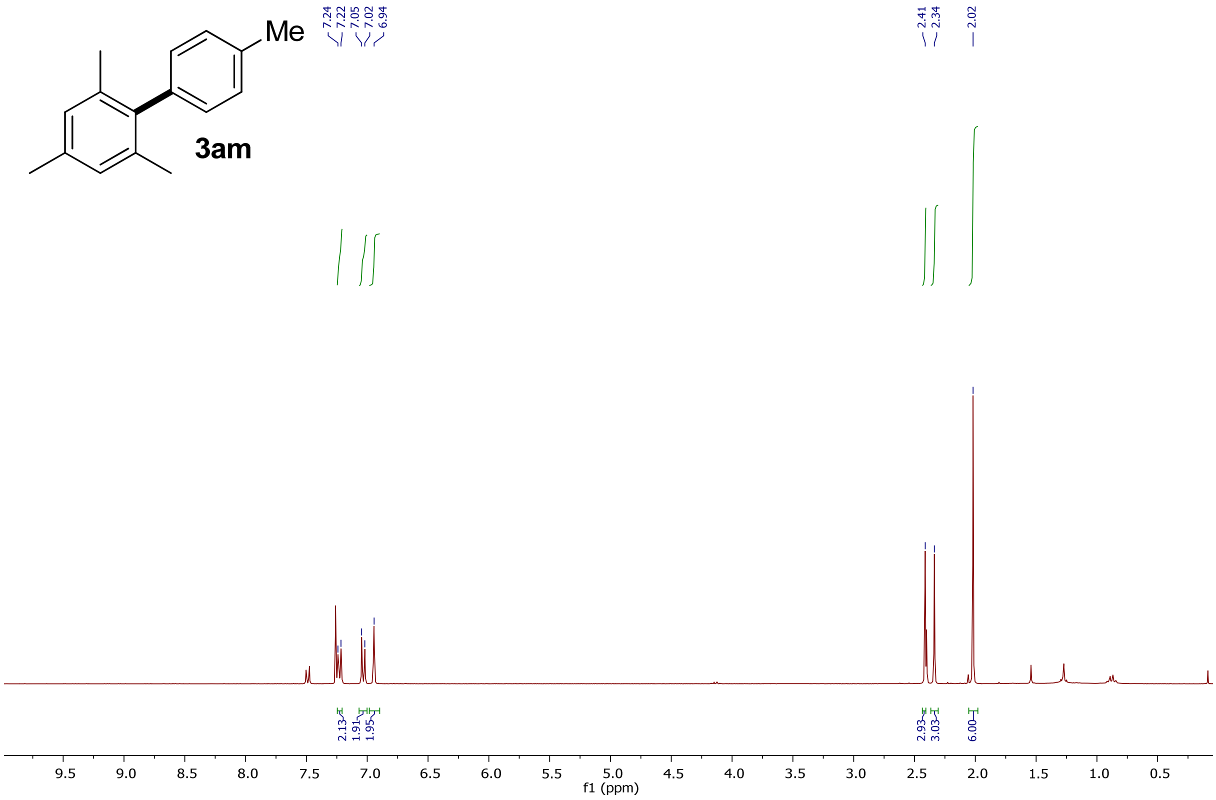

drsc57a4c02.1.fid
13C 75.5MHz Job 58546 Sutherland Daniel R 57A4C02 CDCl3 25.0°C 3 hours 1 min
*

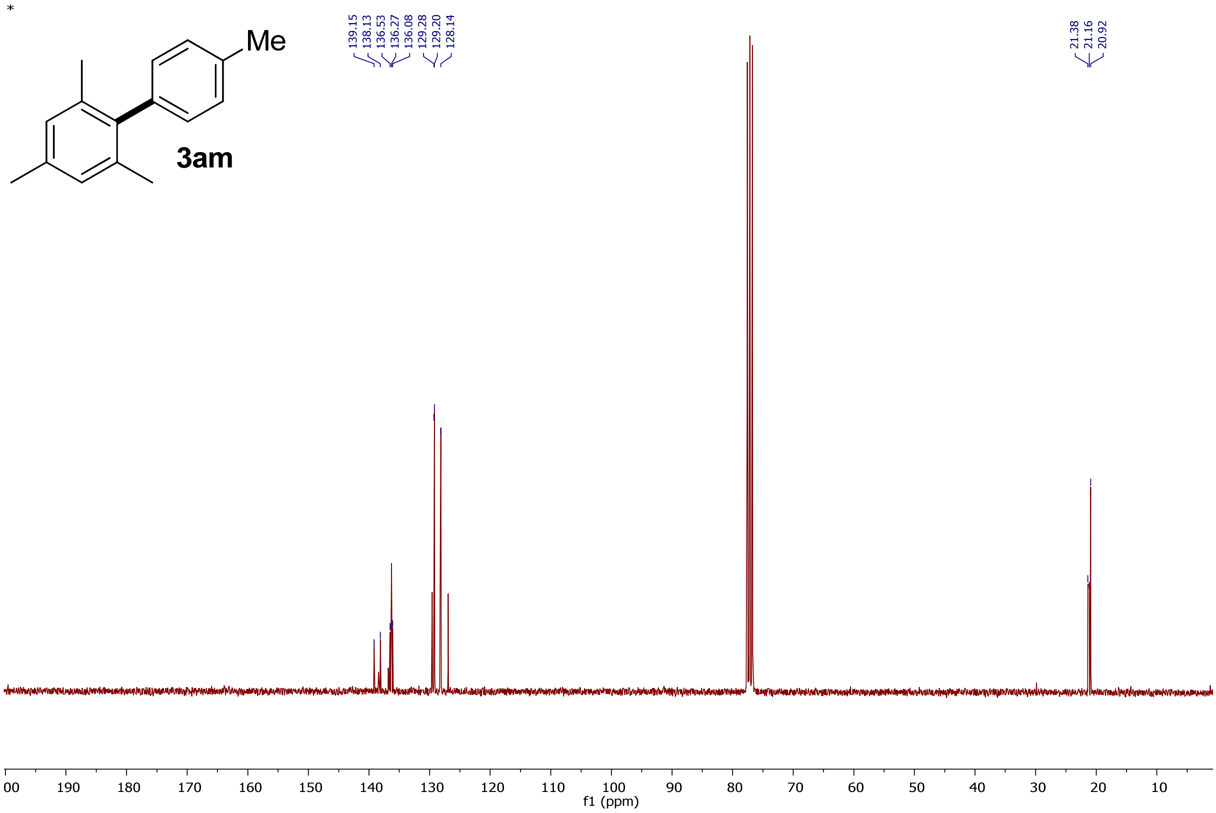

drsh57a4g02.1.fid
1H 300.1MHz Job 58419 Sutherland Daniel R 57A4G02 CDCl3 24.9°C
*

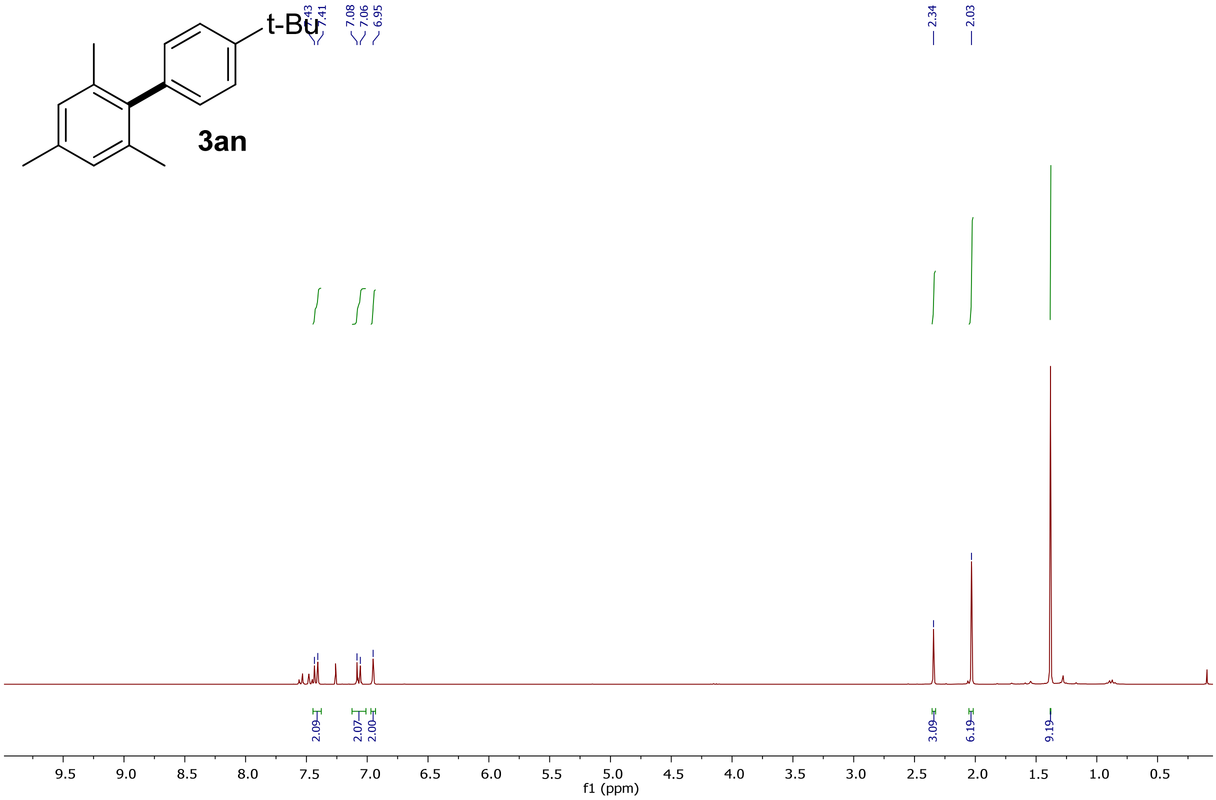

drsc57a4g02.1.fid
13C 75.5MHz Job 58548 Sutherland Daniel R 57A4G02 CDCl3 25.0°C 0 hour 54 min
*

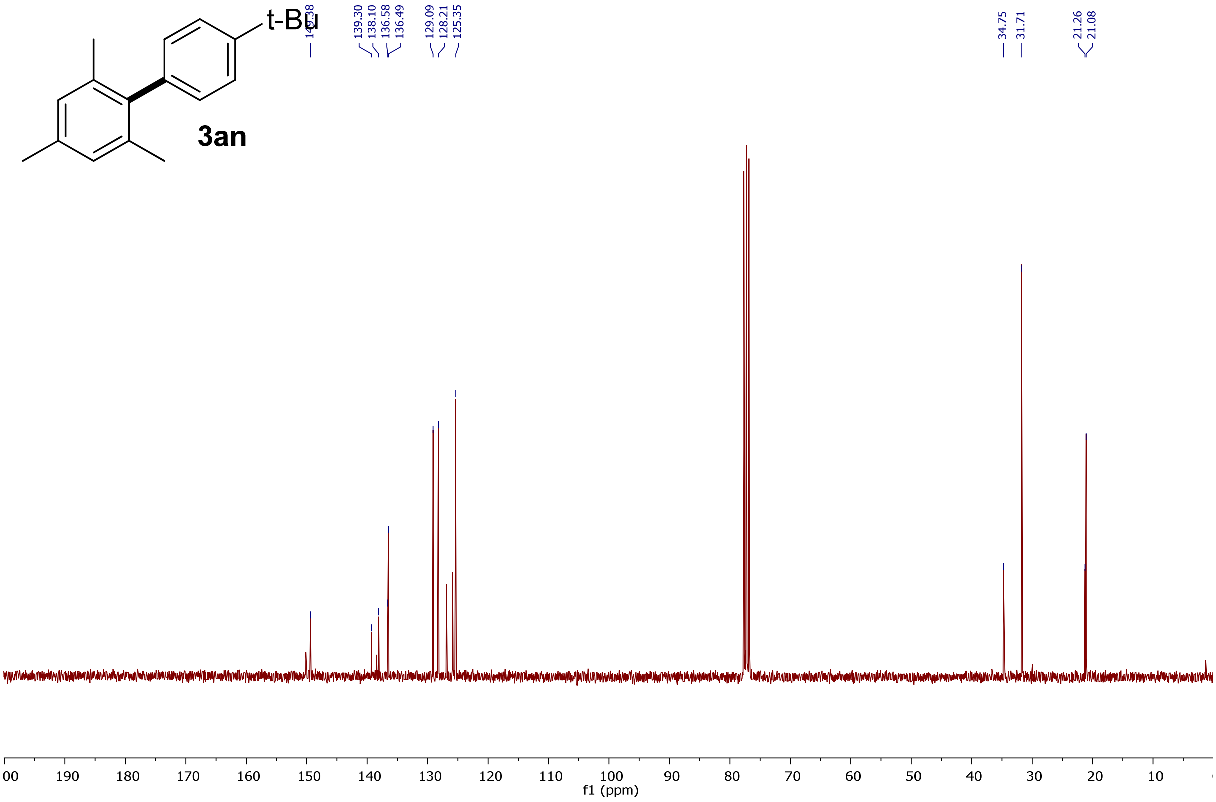

drsh57a4o02.1.fid
1H 300.1MHz Job 59365 Sutherland Daniel R 57A4O02 CDCl3 24.9°C
*

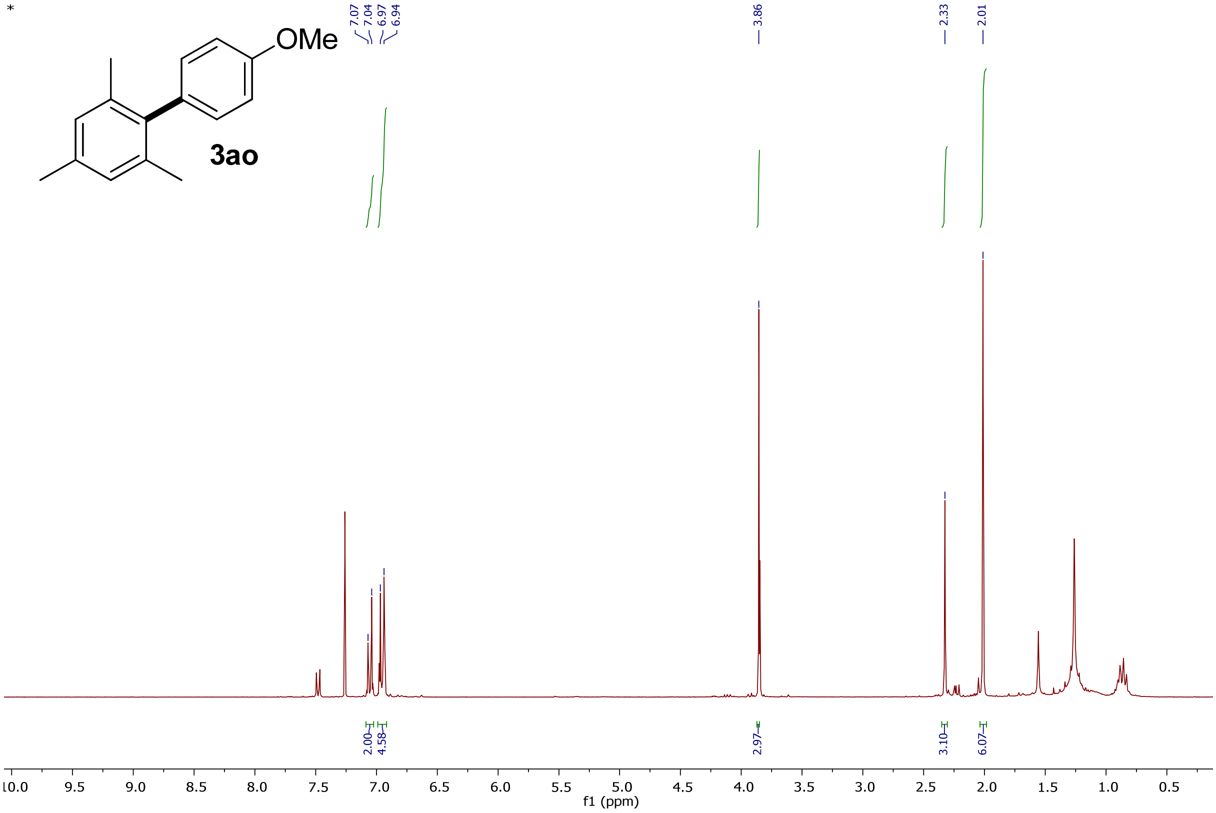

drsc57a4o02.1.fid
13C 75.5MHz Job 59405 Sutherland Daniel R 57A4O02 CDCl3 25.0°C 3 hours 1 min
*

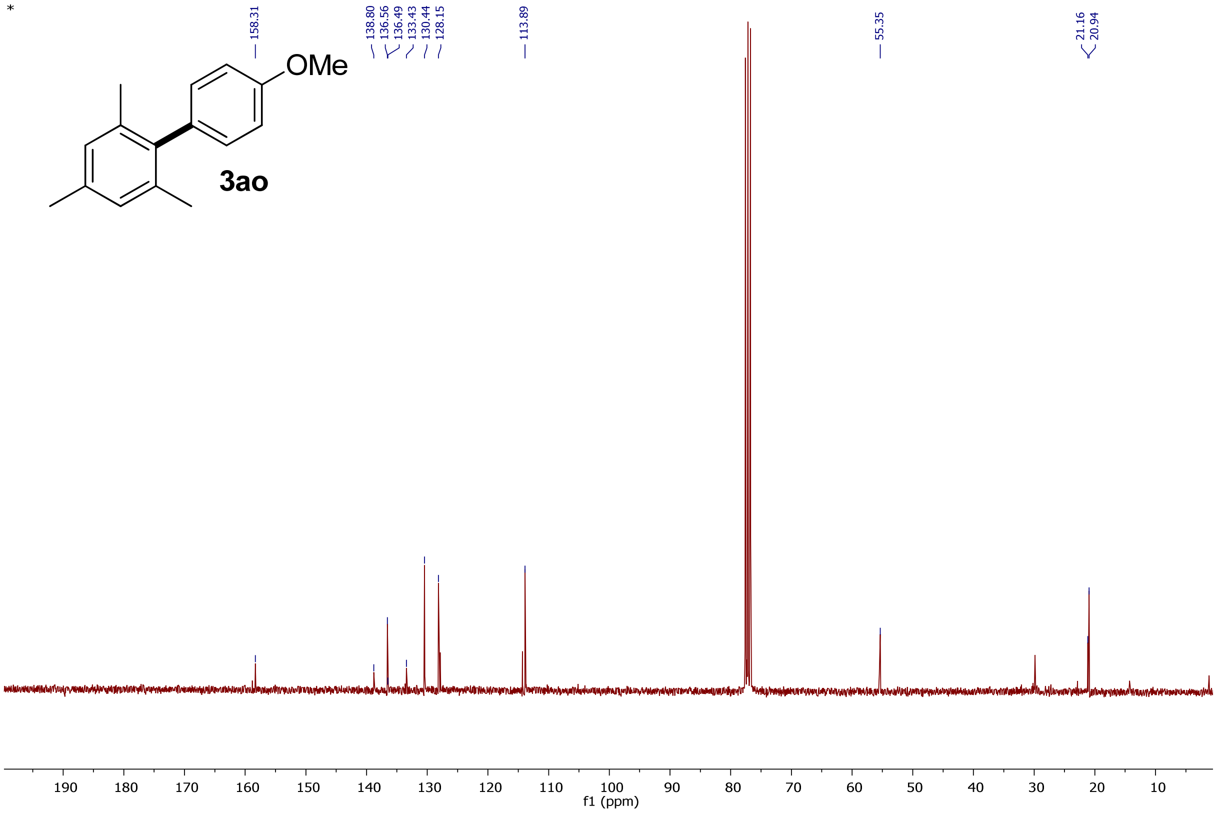

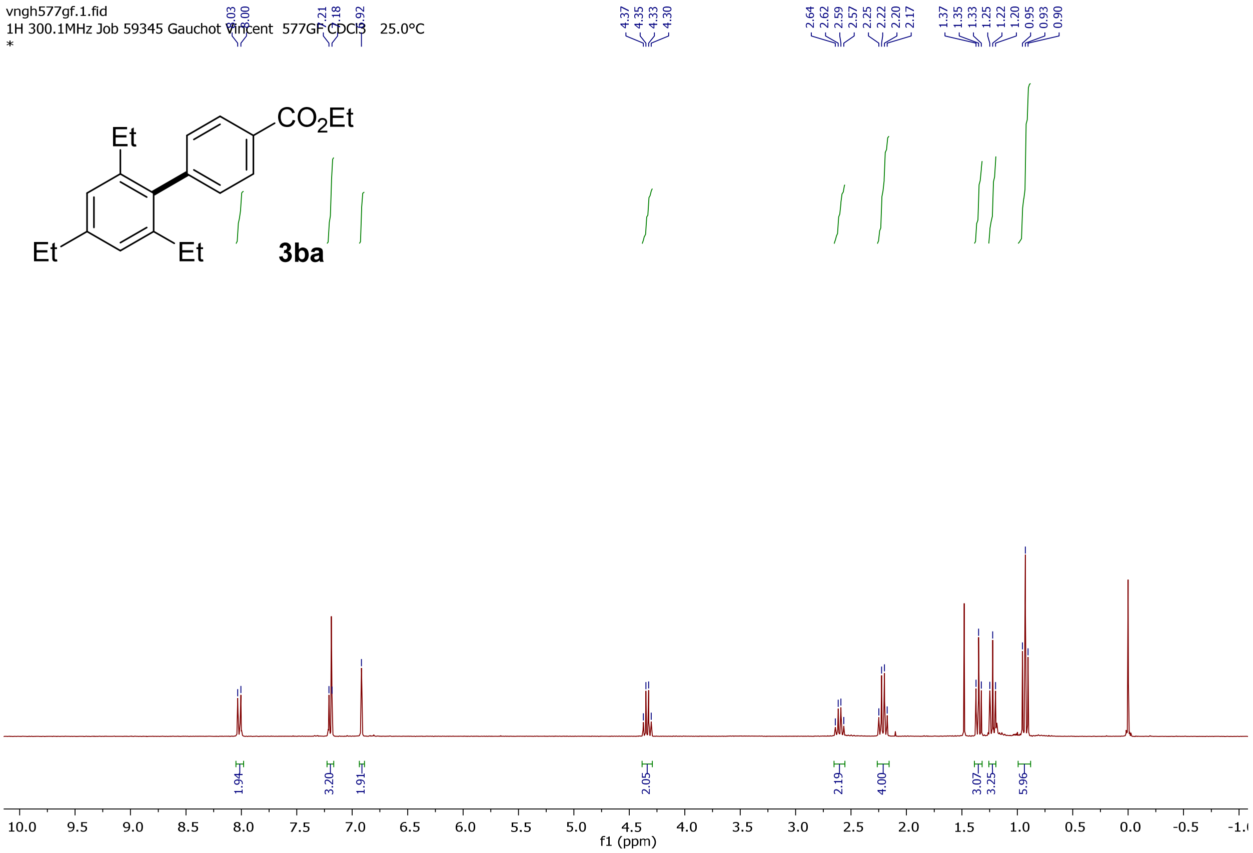
vngh577gf.1.fid
1H 300.1MHz Job 59345 Gauchot Vincent 577GF CDCl3 25.0°C
*
8.03
8.00
7.21
7.18
6.92
4.37
4.35
4.33
4.30
2.64
2.62
2.59
2.57
2.25
2.22
2.20
2.17
1.37
1.35
1.33
1.25
1.22
1.20
0.95
0.93
0.90
CO2Et
Et
Et
Et
3ba
1.94
3.20
1.91
2.05
2.19
4.00
3.07
3.25
5.96
f1 (ppm)

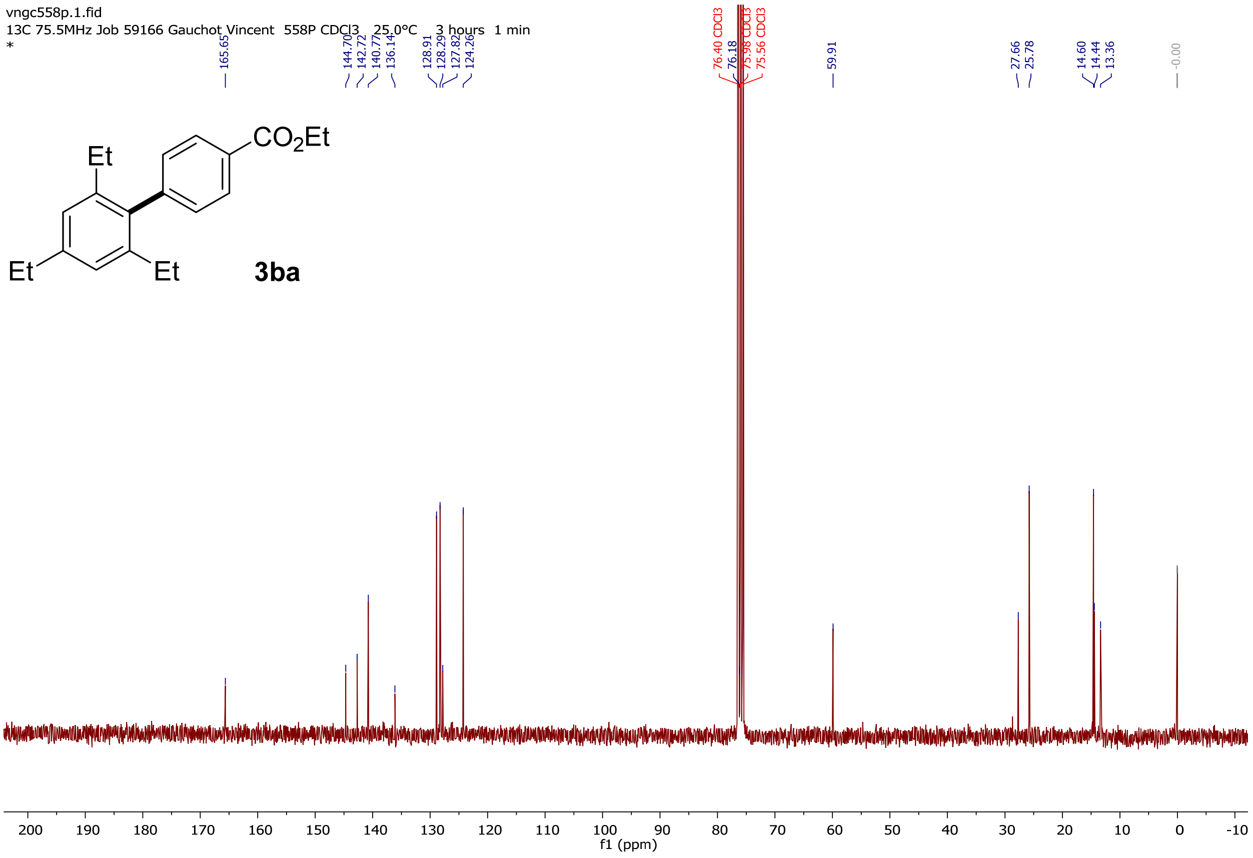
vngc558p.1.fid
13C 75.5MHz Job 59166 Gauchot Vincent 558P CDCl3 25.0°C 3 hours 1 min
*
165.65
144.70
142.72
140.77
136.14
128.91
128.29
127.82
124.26
76.40 CDCl3
76.18
75.98 CDCl3
75.56 CDCl3
59.91
27.66
25.78
14.60
14.44
13.36
-0.00
CO2Et
Et
Et
Et
3ba
f1 (ppm)

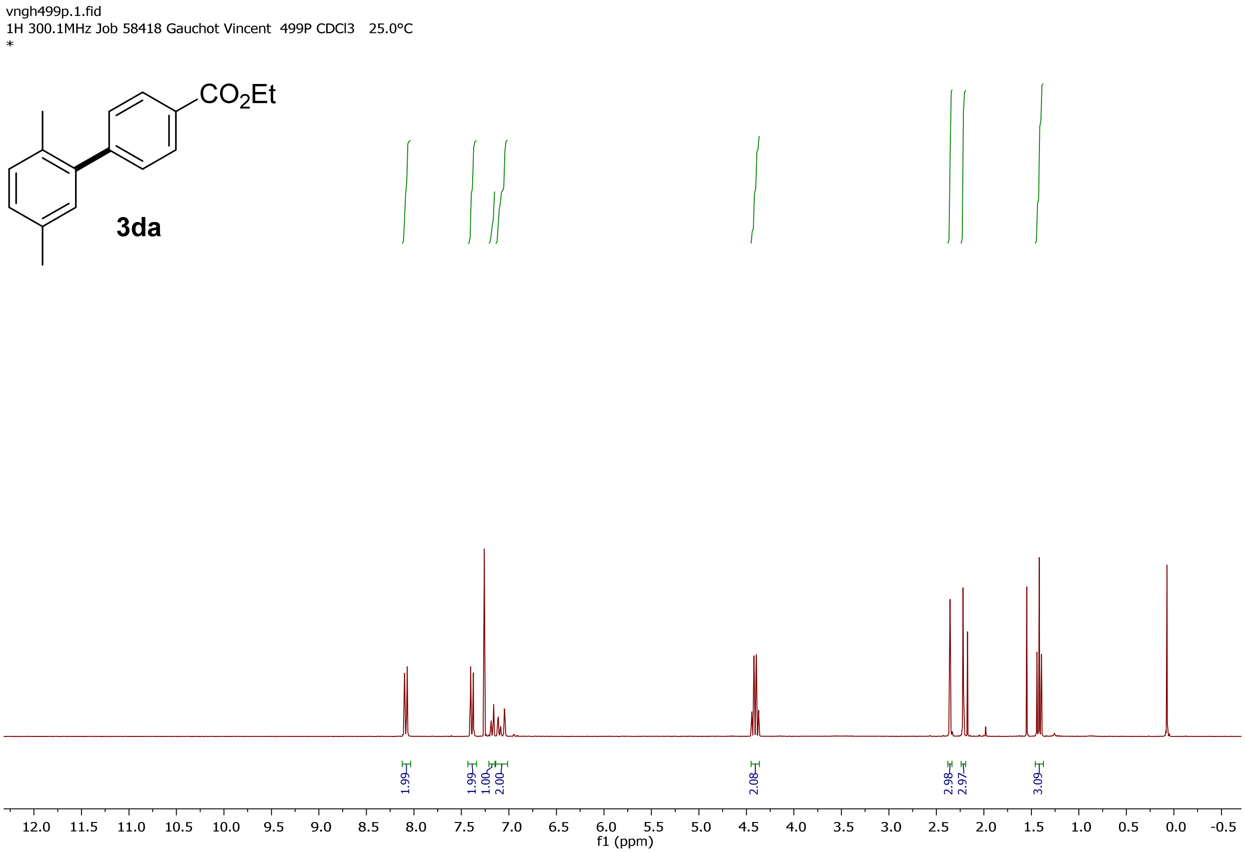
vngh499p.1.fid
1H 300.1MHz Job 58418 Gauchot Vincent 499P CDCl3 25.0°C
*
CO2Et
3da
1.99
1.99
1.00
2.00
2.08
2.98
2.97
3.09
12.0 11.5 11.0 10.5 10.0 9.5 9.0 8.5 8.0 7.5 7.0 6.5 6.0 5.5 5.0 4.5 4.0 3.5 3.0 2.5 2.0 1.5 1.0 0.5 0.0 -0.5
f1 (ppm)

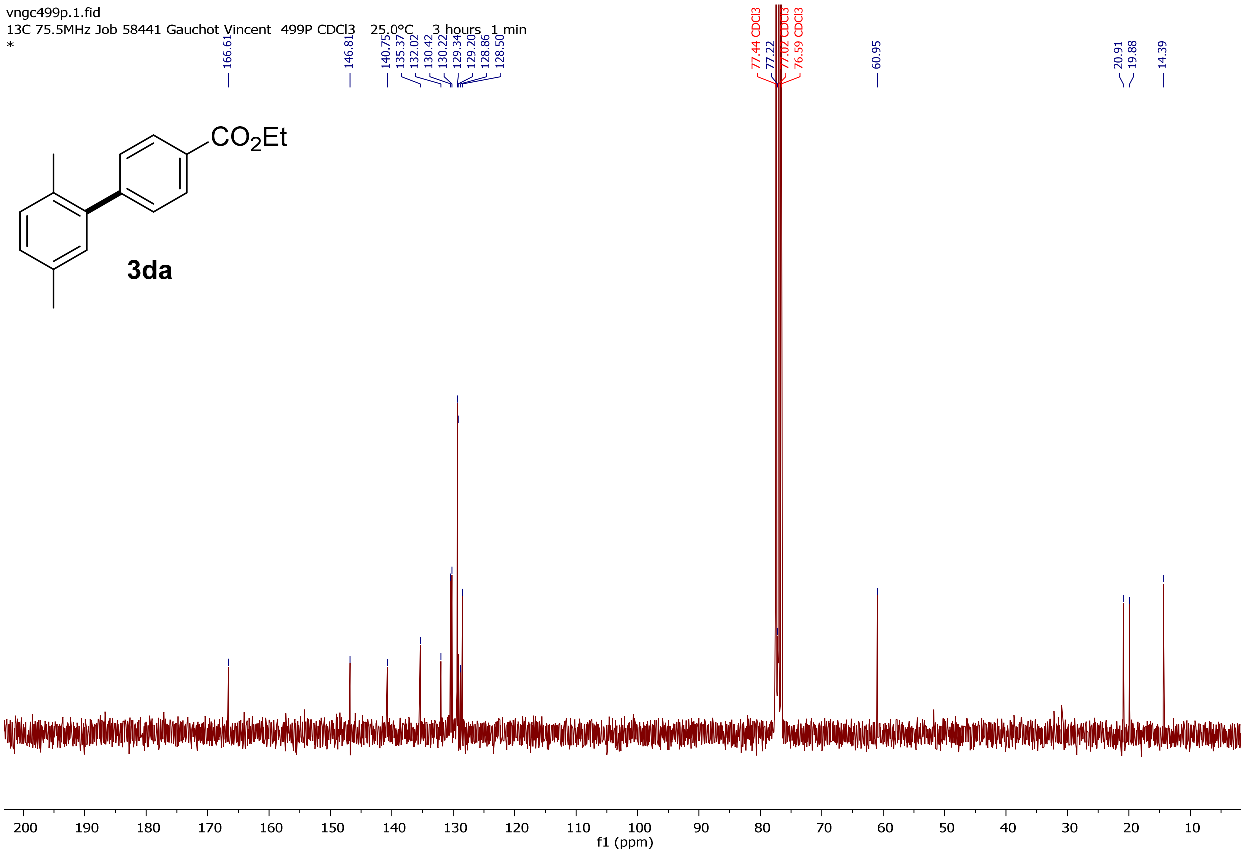
vngc499p.1.fid
13C 75.5MHz Job 58441 Gauchot Vincent 499P CDCl3 25.0°C 3 hours 1 min
*
166.61
146.81
140.75
135.37
132.02
130.42
130.22
129.34
129.20
128.86
128.50
77.44 CDCl3
77.22
77.02 CDCl3
76.59 CDCl3
60.95
20.91
19.88
14.39
CO2Et
3da
200 190 180 170 160 150 140 130 120 110 100 90 80 70 60 50 40 30 20 10
f1 (ppm)

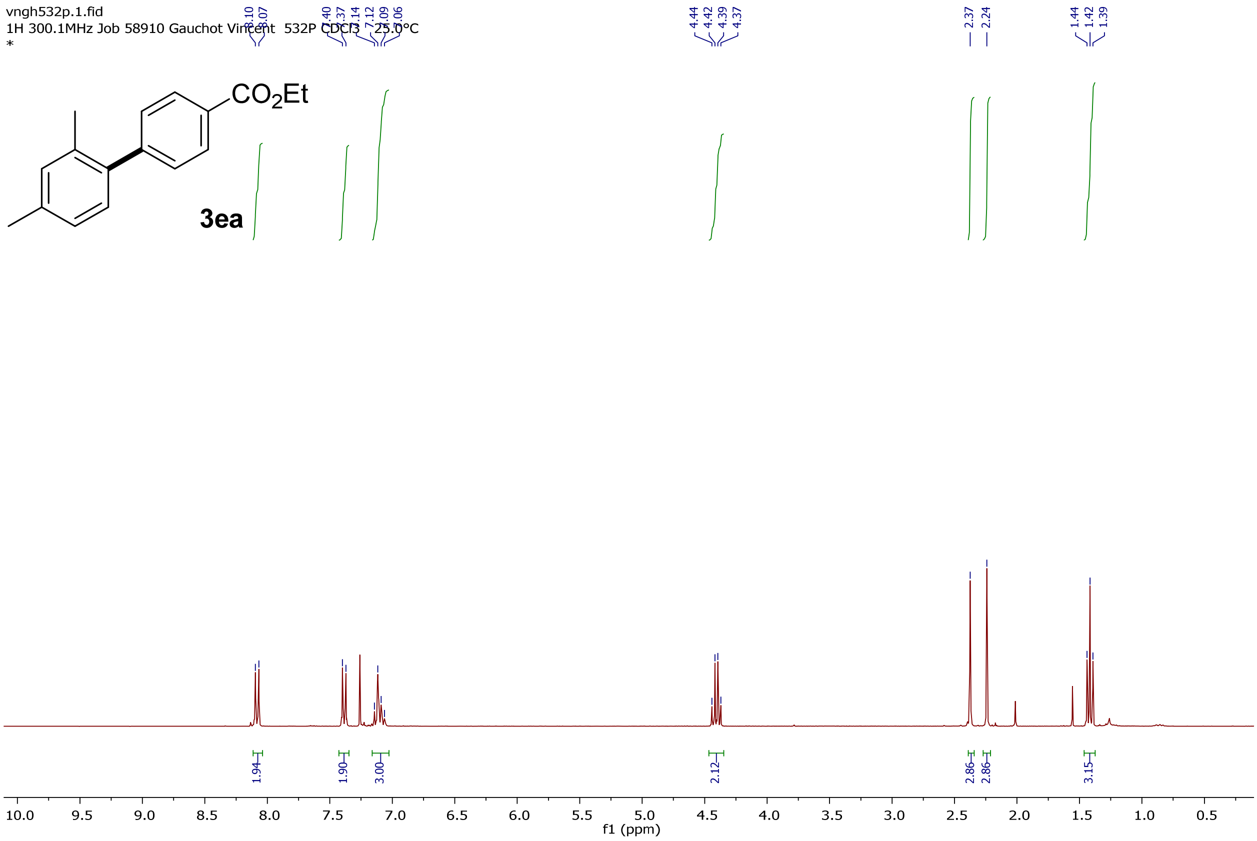
vngh532p.1.fid
1H 300.1MHz Job 58910 Gauchot Vincent 532P CDCl3 25.0°C
*
8.10
8.07
7.40
7.37
7.14
7.12
7.09
7.06
4.44
4.42
4.39
4.37
2.37
2.24
1.44
1.42
1.39
CO2Et
3ea
1.94
1.90
3.00
2.12
2.86
2.86
3.15
10.0 9.5 9.0 8.5 8.0 7.5 7.0 6.5 6.0 5.5 5.0 4.5 4.0 3.5 3.0 2.5 2.0 1.5 1.0 0.5
f1 (ppm)

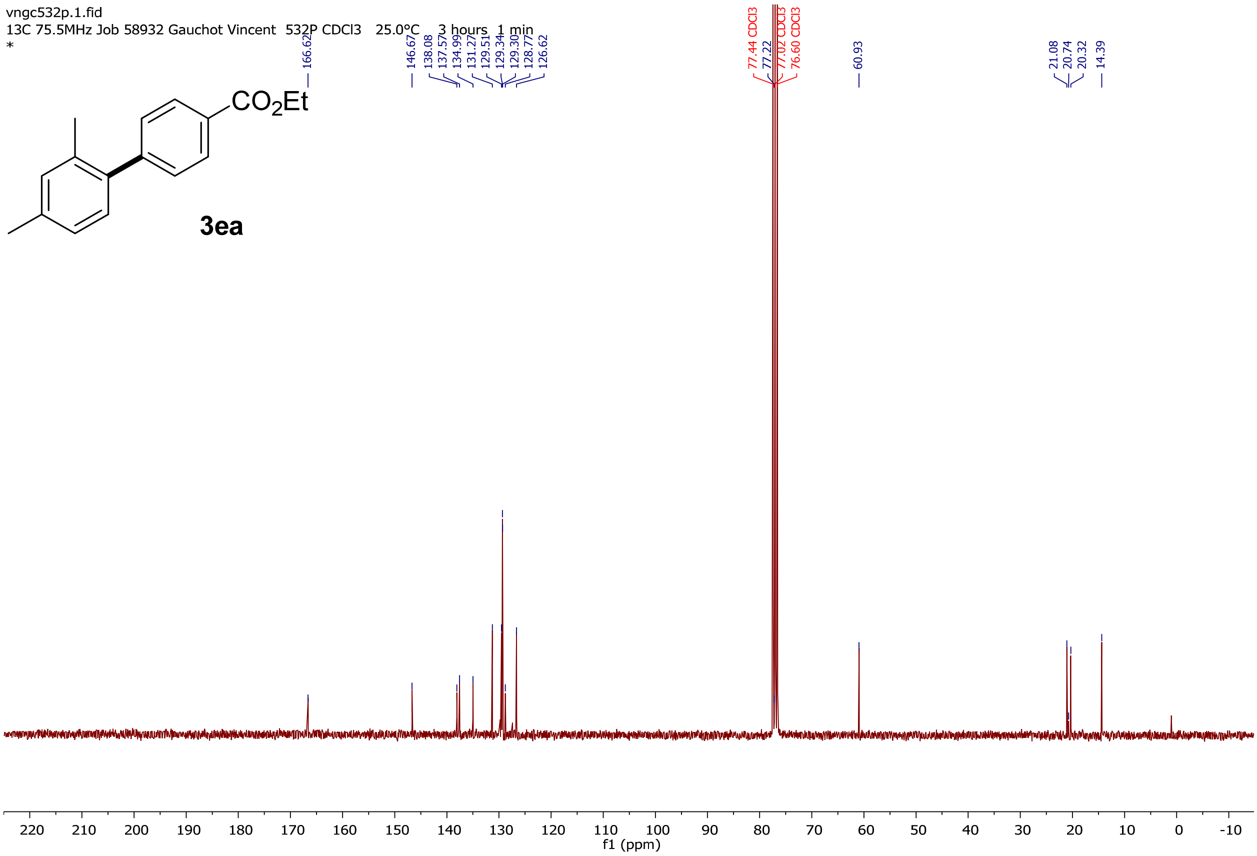
vngc532p.1.fid
13C 75.5MHz Job 58932 Gauchot Vincent 532P CDCl3 25.0°C 3 hours 1 min
*
166.62
146.67
138.08
137.57
134.99
131.27
129.51
129.34
129.30
128.77
126.62
77.44 CDCl3
77.22
77.02 CDCl3
76.60 CDCl3
60.93
21.08
20.74
20.32
14.39
CO2Et
3ea
220 210 200 190 180 170 160 150 140 130 120 110 100 90 80 70 60 50 40 30 20 10 0 -10
f1 (ppm)

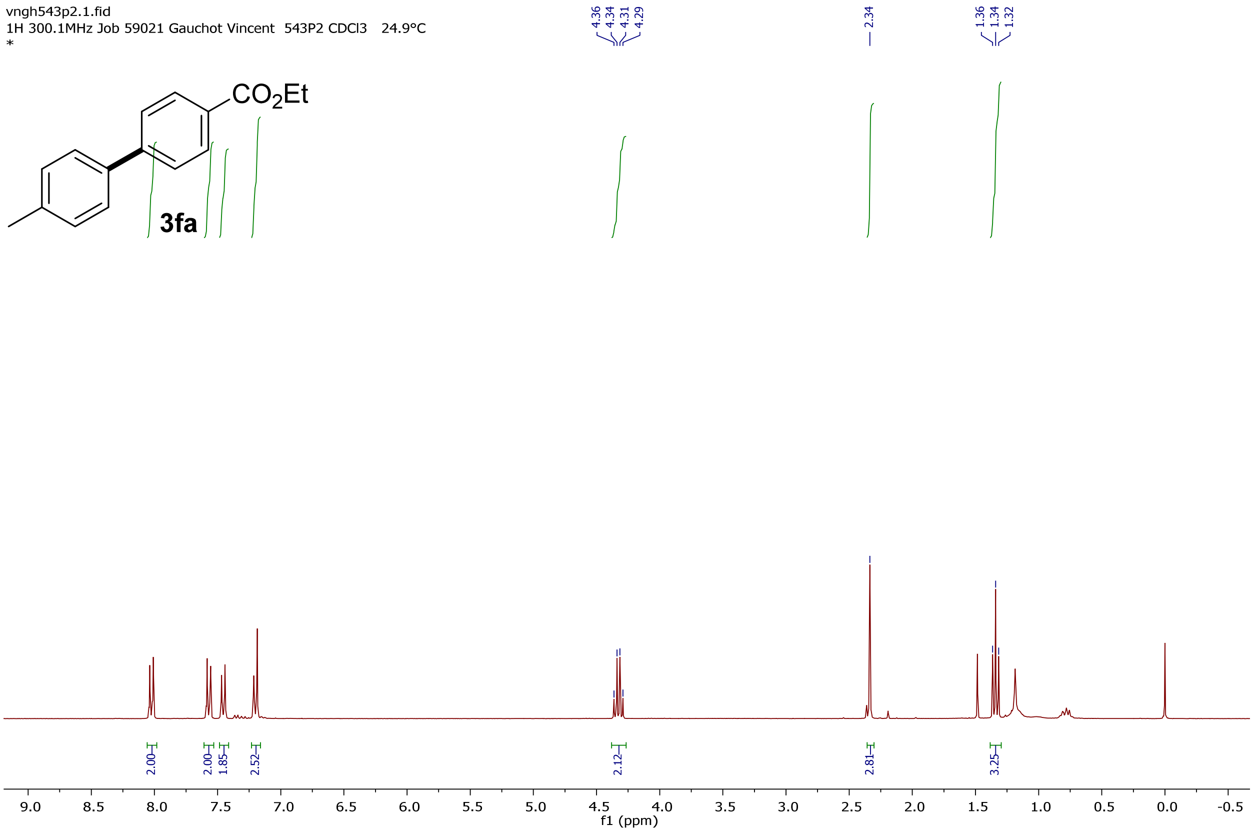
vngh543p2.1.fid
1H 300.1MHz Job 59021 Gauchot Vincent 543P2 CDCl3 24.9°C
*
4.36
4.34
4.31
4.29
2.34
1.36
1.34
1.32
CO2Et
3fa
2.00
2.00
1.85
2.52
2.12
2.81
3.25
9.0 8.5 8.0 7.5 7.0 6.5 6.0 5.5 5.0 4.5 4.0 3.5 3.0 2.5 2.0 1.5 1.0 0.5 0.0 -0.5
f1 (ppm)

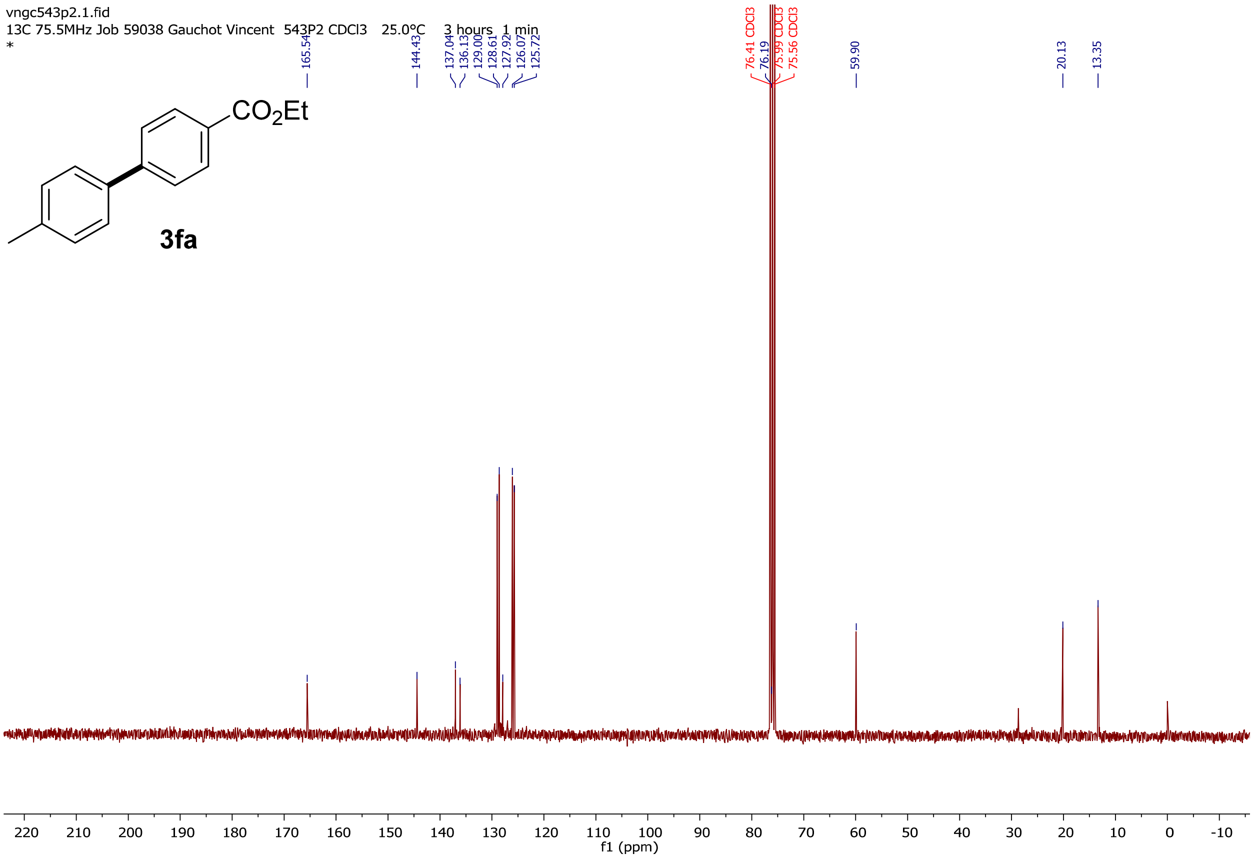
vngc543p2.1.fid
13C 75.5MHz Job 59038 Gauchot Vincent 543P2 CDCl3 25.0°C 3 hours 1 min
*
165.54
144.43
137.04
136.13
129.00
128.61
127.92
126.07
125.72
76.41 CDCl3
76.19
75.99 CDCl3
75.56 CDCl3
59.90
20.13
13.35
CO2Et
3fa
220 210 200 190 180 170 160 150 140 130 120 110 100 90 80 70 60 50 40 30 20 10 0 -10
f1 (ppm)

vngh586p.1.fid
1H 300.1MHz Job 59375 Gauchot Vincent 586P CDCl3 25.0°C
*

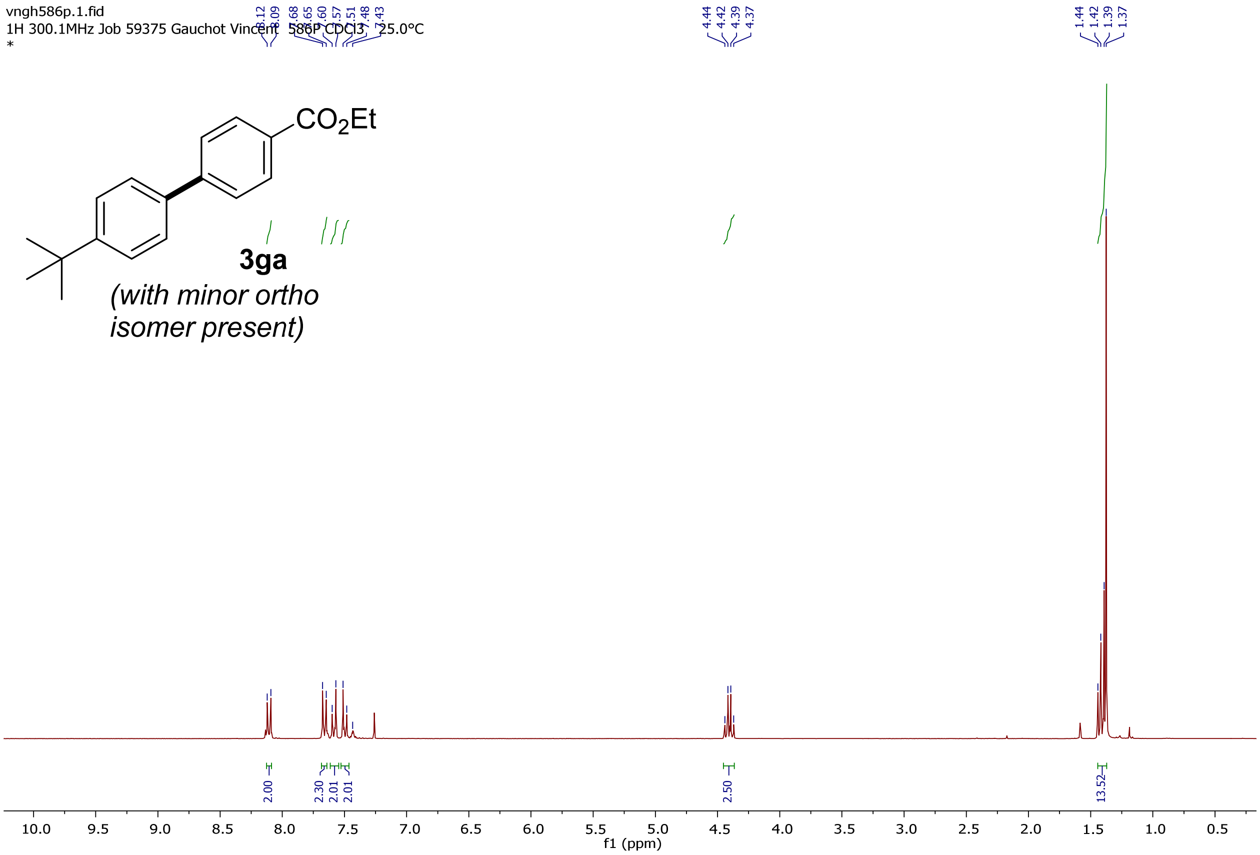

vngc586p.1.fid
13C 75.5MHz Job 59404 Gauchot Vincent 586P CDCl3 25.0°C 1 hour 30 min
*

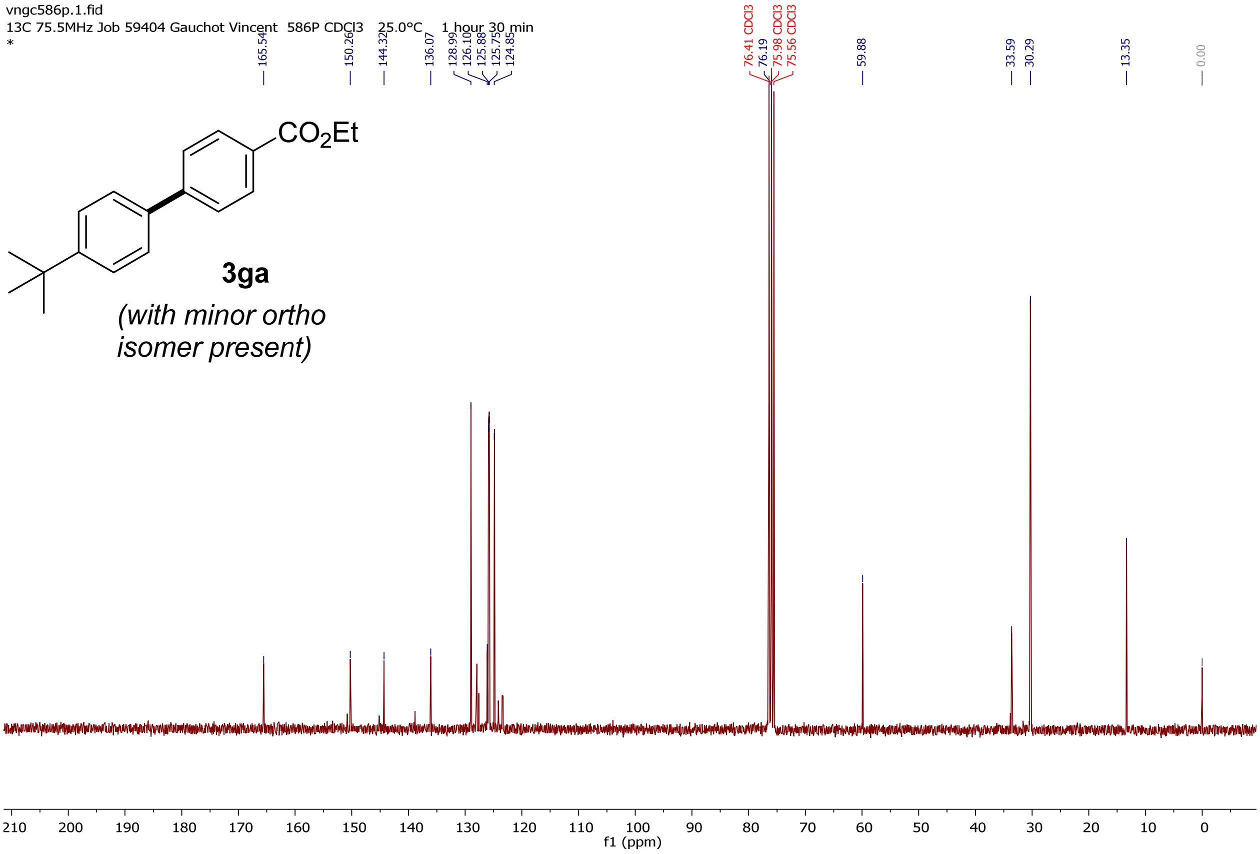

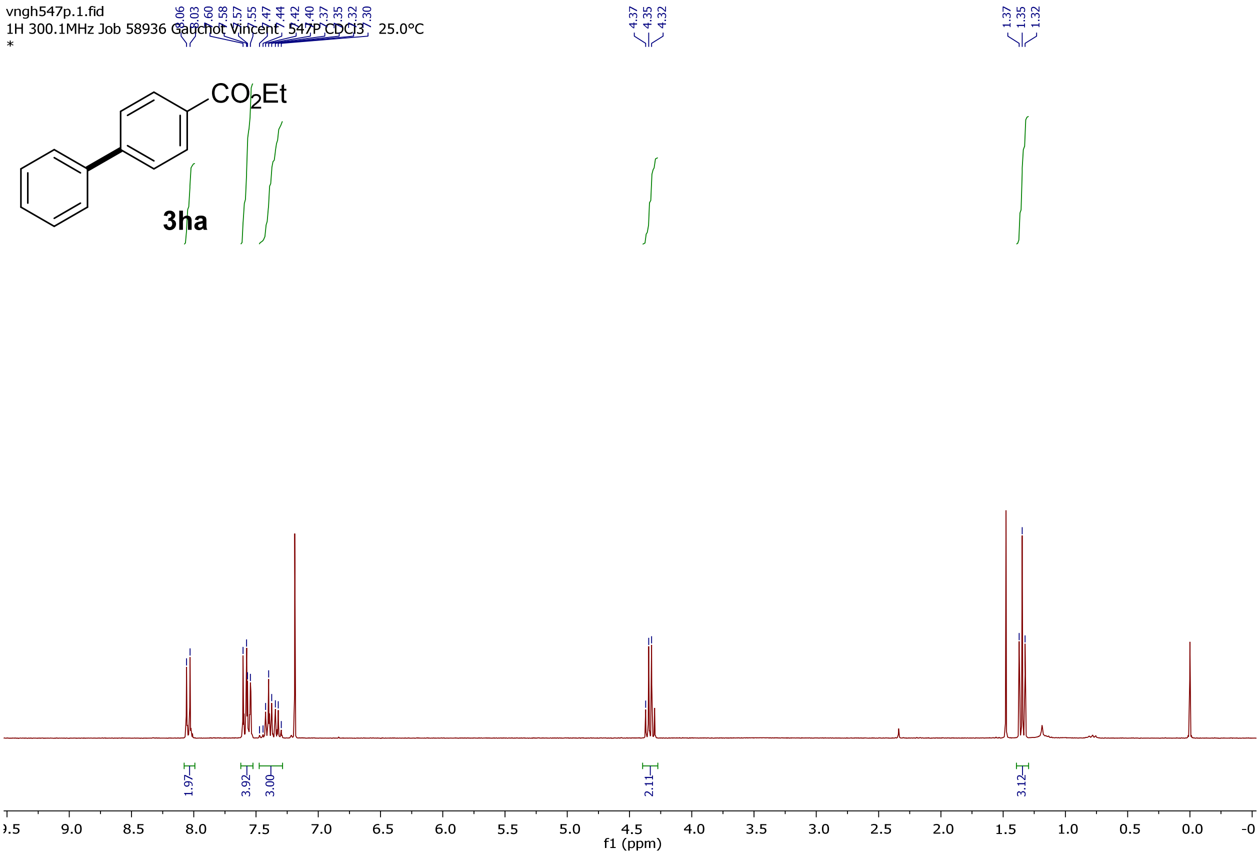
vngh547p.1.fid
1H 300.1MHz Job 58936 Gauchot Vincent 547P CDCl3 25.0°C
*
CO2Et
3ha
4.37
4.35
4.32
1.37
1.35
1.32
1.97
3.92
3.00
2.11
3.12
f1 (ppm)

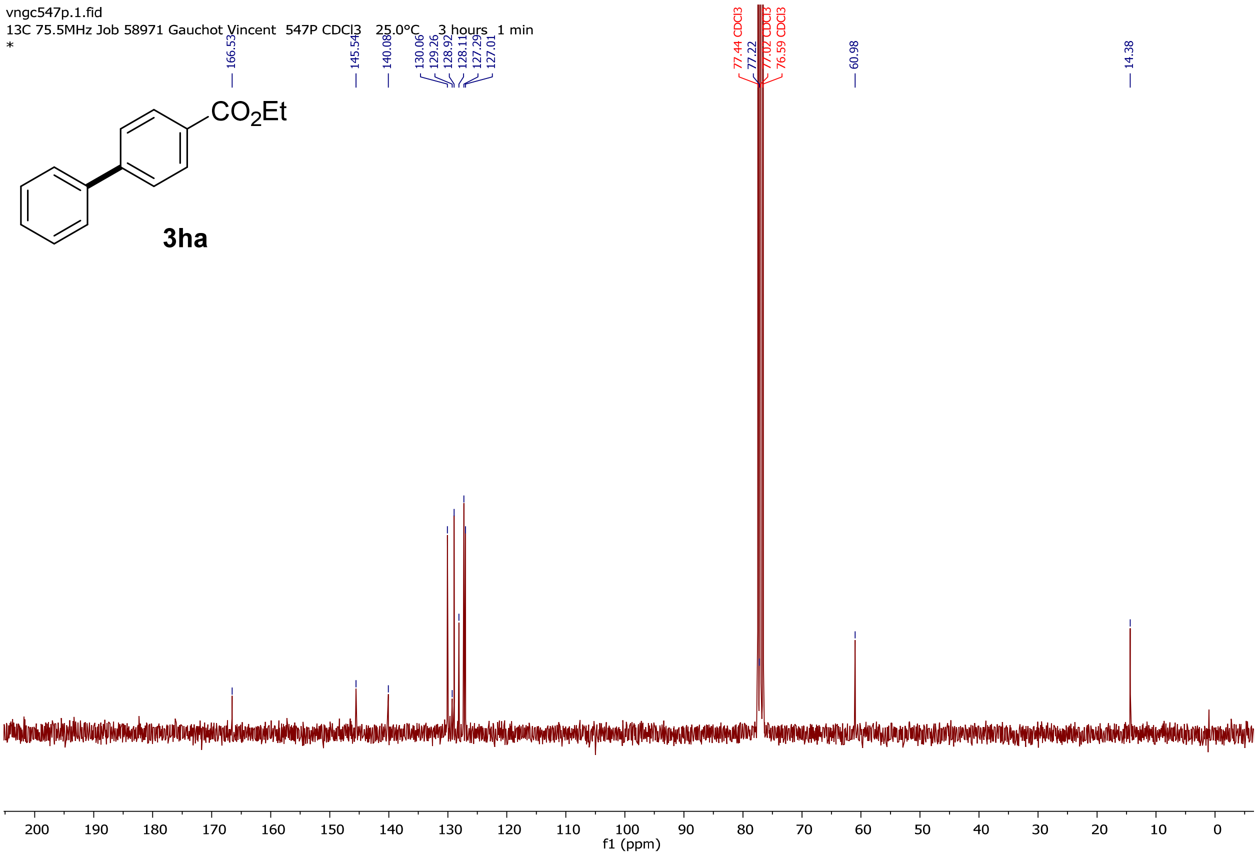
vngc547p.1.fid
13C 75.5MHz Job 58971 Gauchot Vincent 547P CDCl3 25.0°C 3 hours 1 min
*
166.53
145.54
140.08
130.06
129.26
128.92
128.11
127.29
127.01
77.44 CDCl3
77.22
77.02 CDCl3
76.59 CDCl3
60.98
14.38
CO2Et
3ha
f1 (ppm)

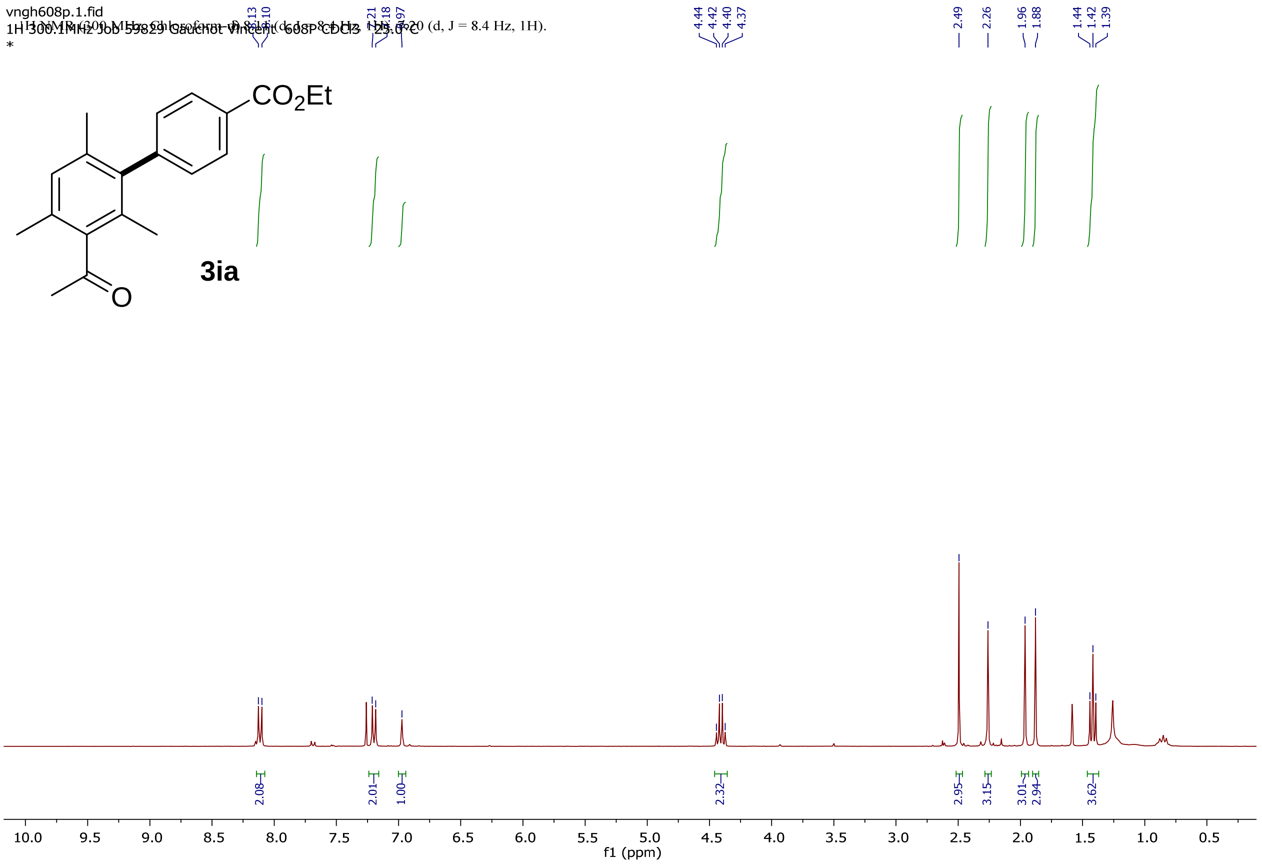

vngh608p.1.fid
1H 300.1MHz Job 59829 Gauchot Vincent 608P CDCl3 25.0°C
8.13
8.10
7.21
7.18
6.97
4.44
4.42
4.40
4.37
2.49
2.26
1.96
1.88
1.44
1.42
1.39
CO_2Et
O
3ia
2.08
2.01
1.00
2.32
2.95
3.15
3.01
2.94
3.62
10.0 9.5 9.0 8.5 8.0 7.5 7.0 6.5 6.0 5.5 5.0 4.5 4.0 3.5 3.0 2.5 2.0 1.5 1.0 0.5
f1 (ppm)

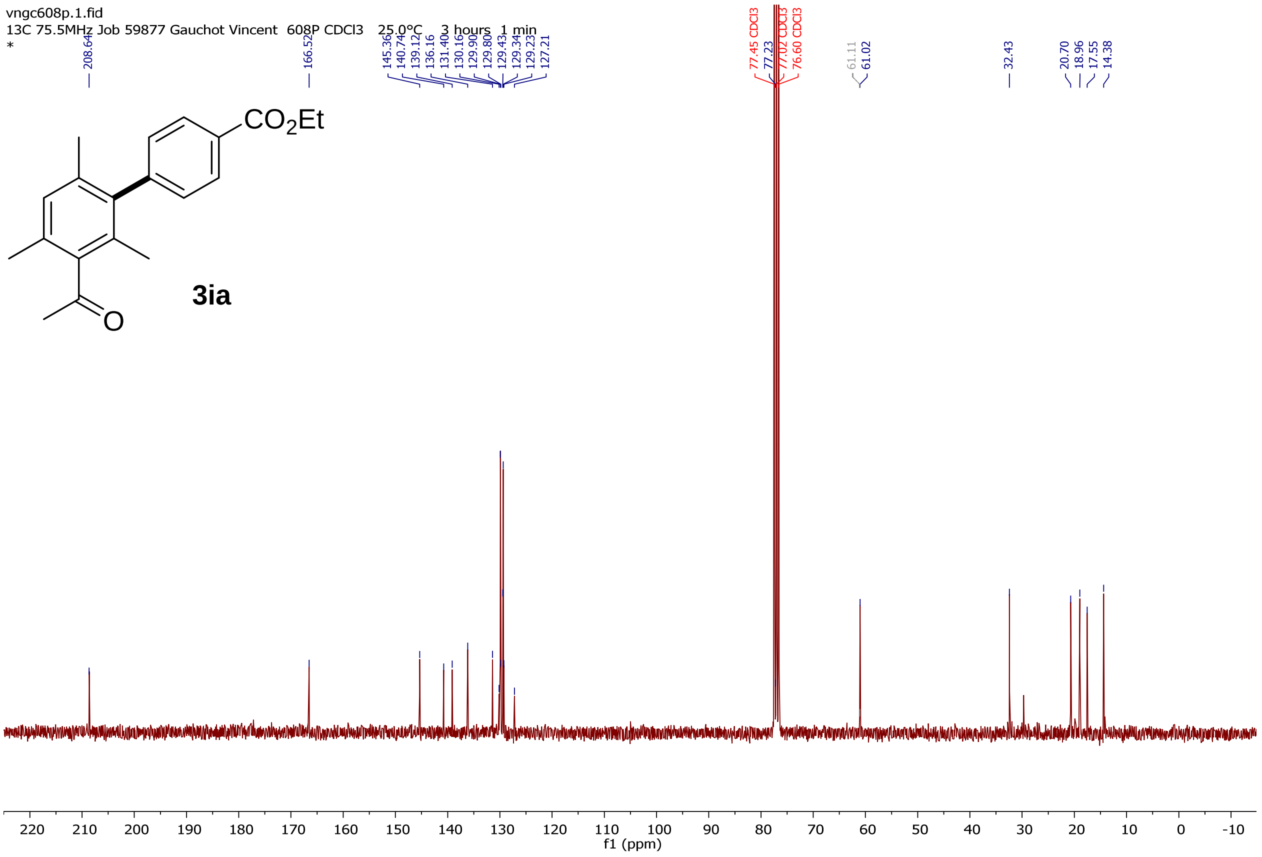

vngc608p.1.fid
13C 75.5MHz Job 59877 Gauchot Vincent 608P CDCl3 25.0°C 3 hours 1 min
208.64
166.52
145.36
140.74
139.12
136.16
131.40
130.16
129.90
129.80
129.43
129.34
129.23
127.21
77.45 CDCl3
77.23
77.02 CDCl3
76.60 CDCl3
61.11
61.02
32.43
20.70
18.96
17.55
14.38
CO_2Et
O
3ia
220 210 200 190 180 170 160 150 140 130 120 110 100 90 80 70 60 50 40 30 20 10 0 -10
f1 (ppm)

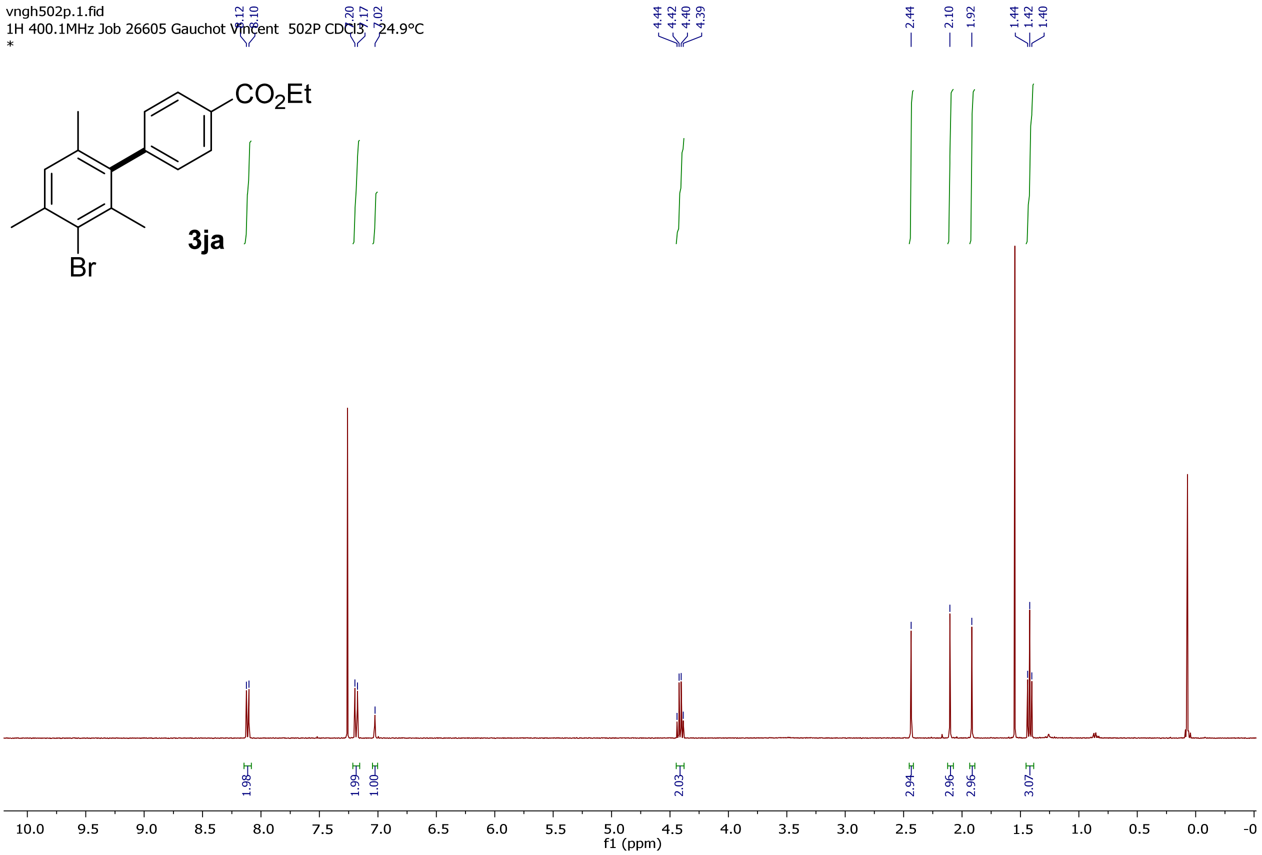
vngh502p.1.fid
1H 400.1MHz Job 26605 Gauchot Vincent 502P CDCl3 24.9°C
CO2Et
Br
3ja
f1 (ppm)

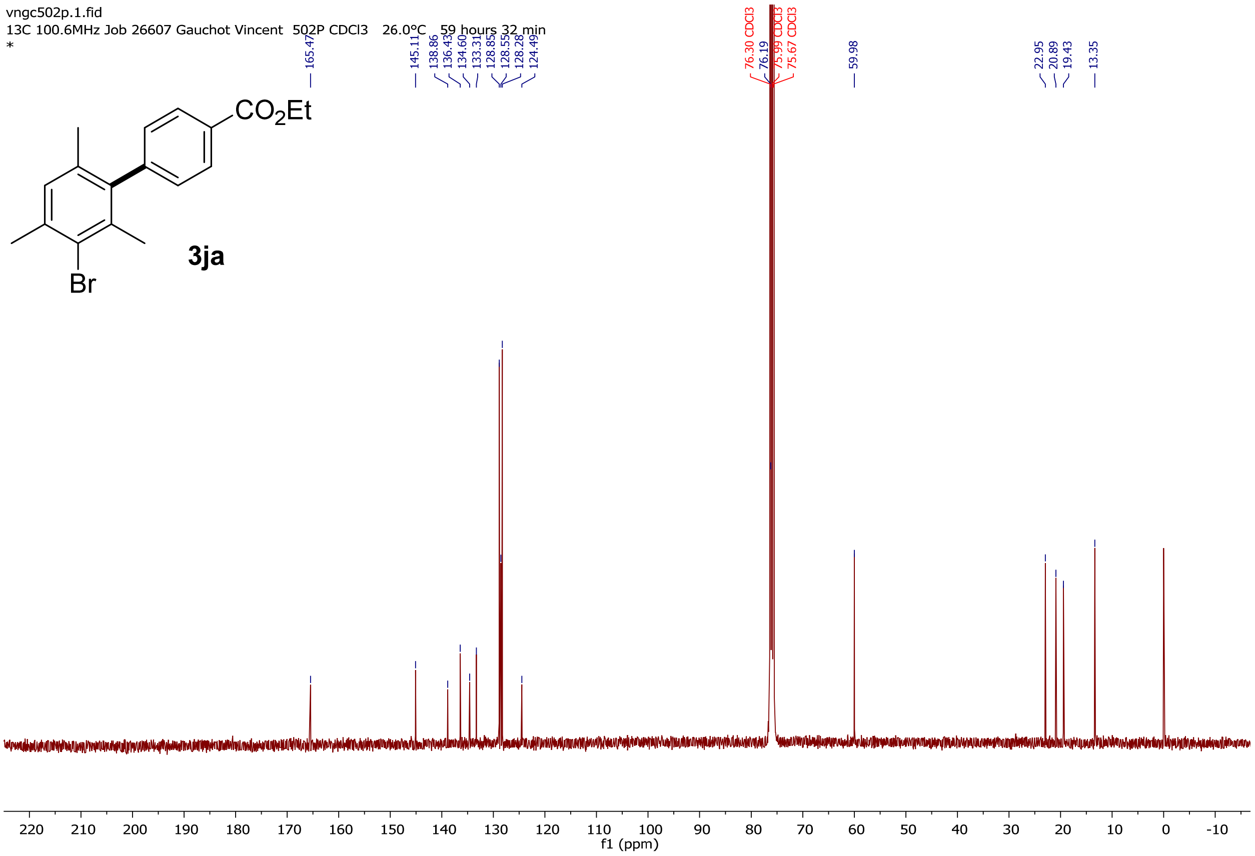
vngc502p.1.fid
13C 100.6MHz Job 26607 Gauchot Vincent 502P CDCl3 26.0°C 59 hours 32 min
CO2Et
Br
3ja
f1 (ppm)

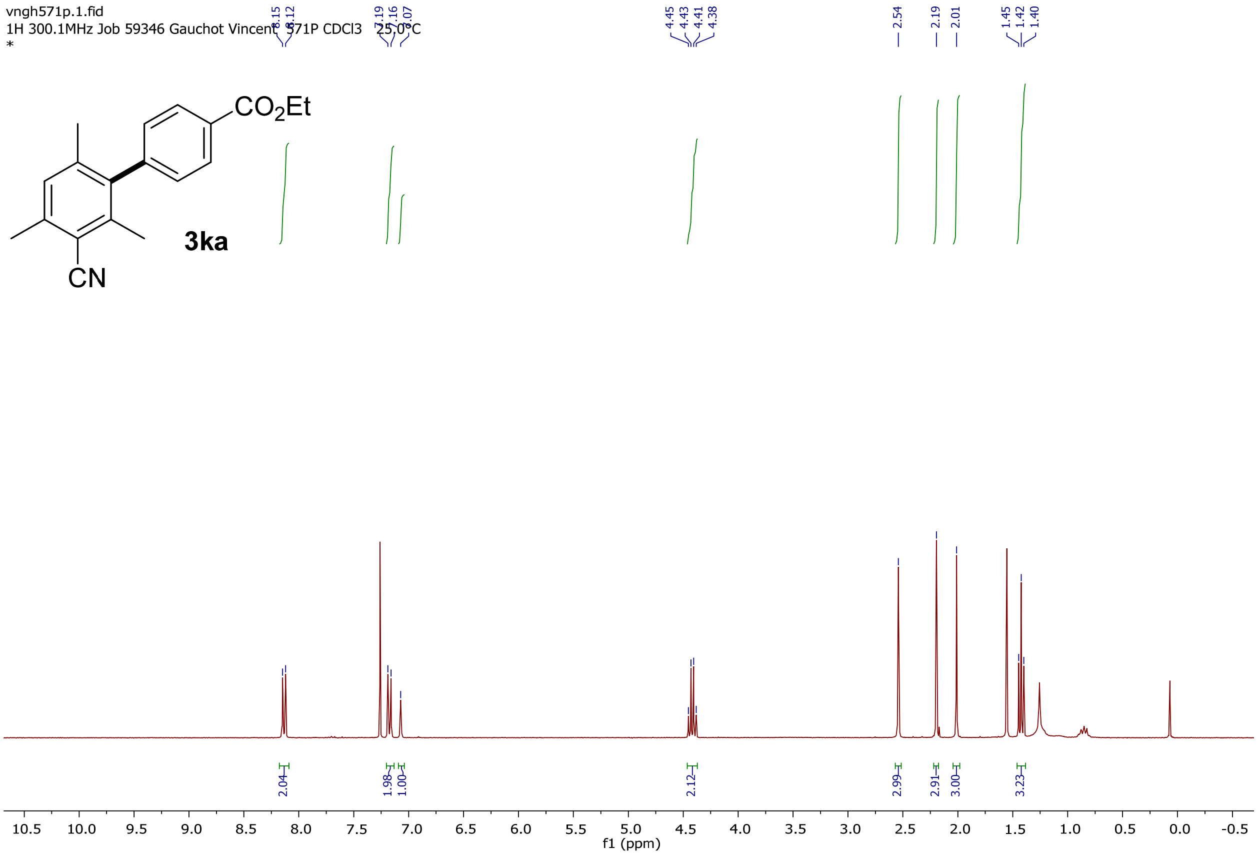
vngh571p.1.fid
1H 300.1MHz Job 59346 Gauchot Vincent 571P CDCl3 25.0°C
*
8.15
8.12
7.19
7.16
7.07
4.45
4.43
4.41
4.38
2.54
2.19
2.01
1.45
1.42
1.40
CO2Et
CN
3ka
2.04
1.98
1.00
2.12
2.99
2.91
3.00
3.23
f1 (ppm)

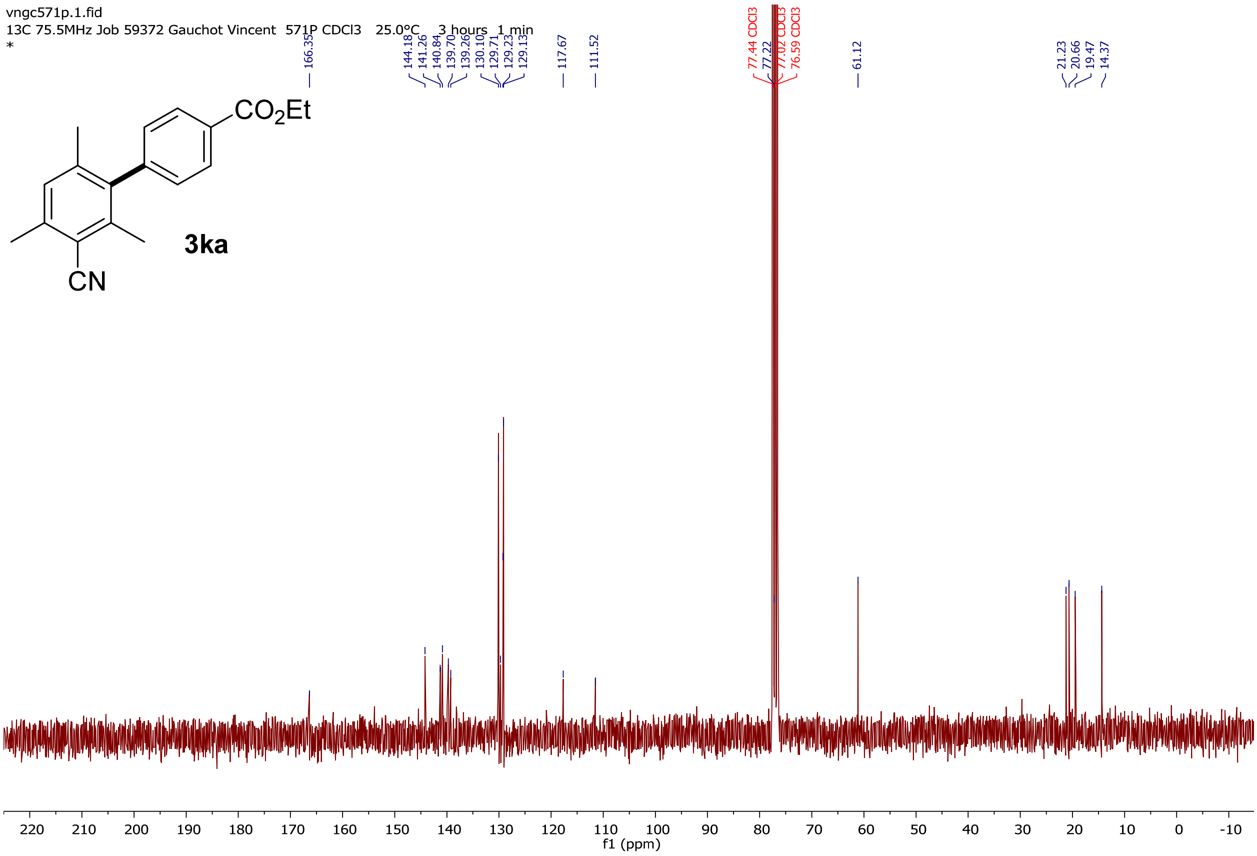
vngc571p.1.fid
13C 75.5MHz Job 59372 Gauchot Vincent 571P CDCl3 25.0°C 3 hours 1 min
*
166.35
144.18
141.26
140.84
139.70
139.26
130.10
129.71
129.23
129.13
117.67
111.52
77.44 CDCl3
77.22
77.02 CDCl3
76.59 CDCl3
61.12
21.23
20.66
19.47
14.37
CO2Et
CN
3ka
f1 (ppm)

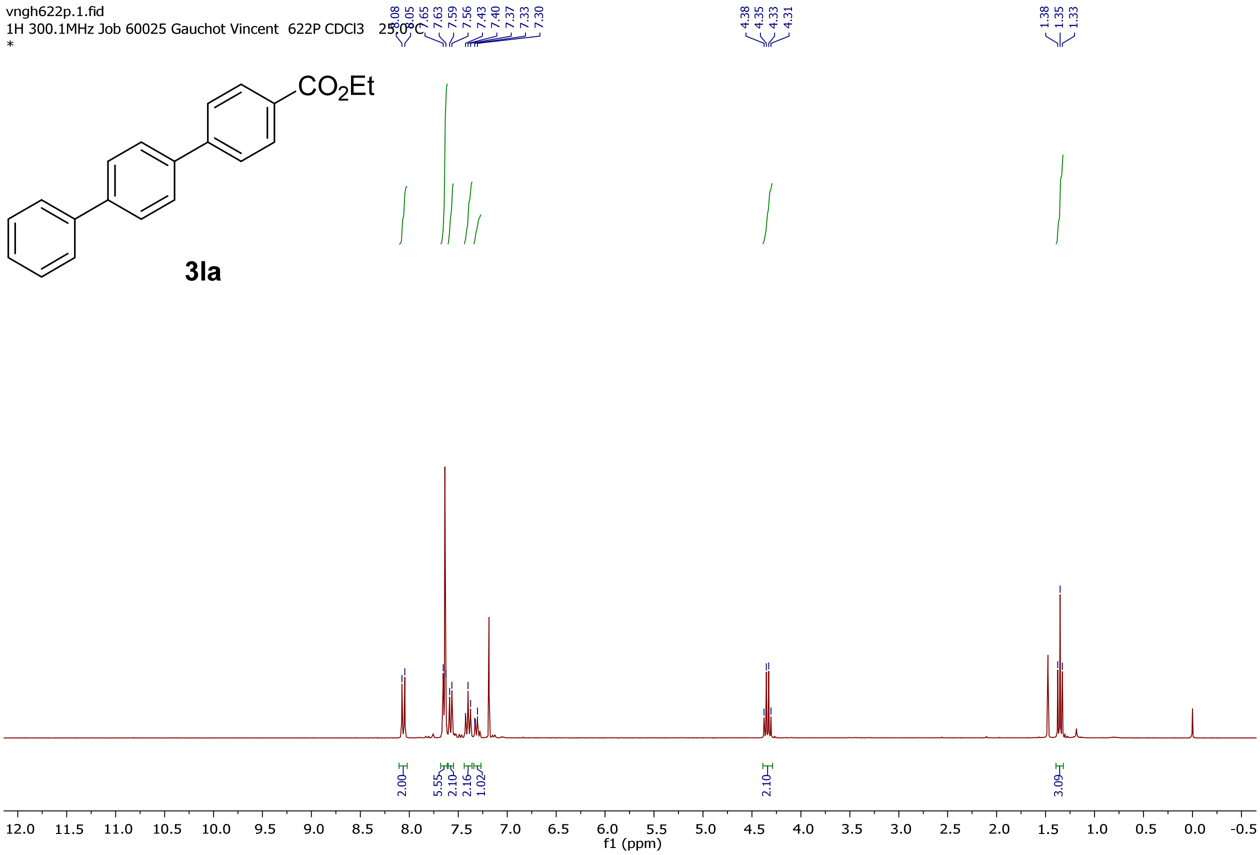
vngh622p.1.fid
1H 300.1MHz Job 60025 Gauchot Vincent 622P CDCl3 25.0°C
*
8.08
8.05
7.65
7.63
7.59
7.56
7.43
7.40
7.37
7.33
7.30
4.38
4.35
4.33
4.31
1.38
1.35
1.33
CO_2Et
3la
2.00
5.55
2.10
2.16
1.02
2.10
3.09
12.0 11.5 11.0 10.5 10.0 9.5 9.0 8.5 8.0 7.5 7.0 6.5 6.0 5.5 5.0 4.5 4.0 3.5 3.0 2.5 2.0 1.5 1.0 0.5 0.0 -0.5
f1 (ppm)

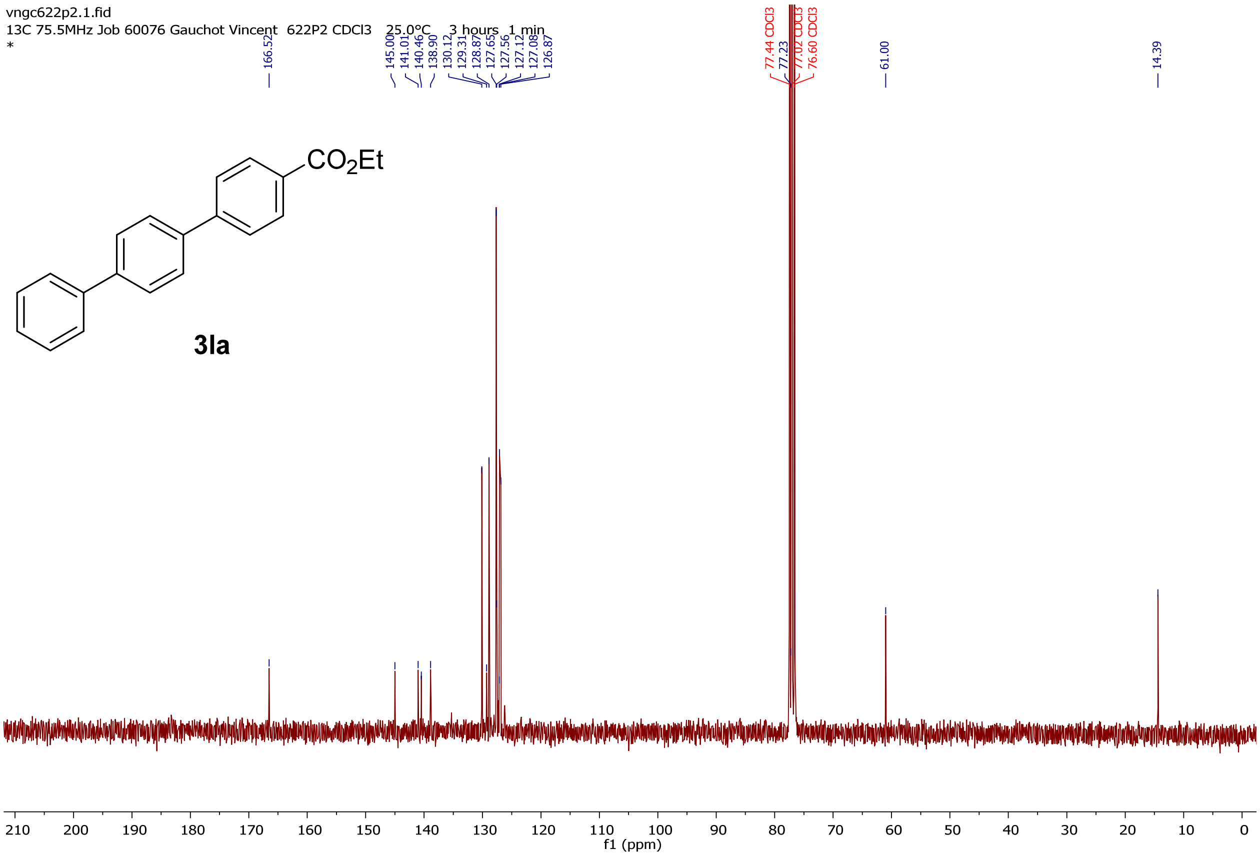
vngc622p2.1.fid
13C 75.5MHz Job 60076 Gauchot Vincent 622P2 CDCl3 25.0°C 3 hours 1 min
*
166.52
145.00
141.01
140.46
138.90
130.12
129.31
128.87
127.65
127.56
127.12
127.08
126.87
77.44 CDCl3
77.23
77.02 CDCl3
76.60 CDCl3
61.00
14.39
CO_2Et
3la
210 200 190 180 170 160 150 140 130 120 110 100 90 80 70 60 50 40 30 20 10 0
f1 (ppm)

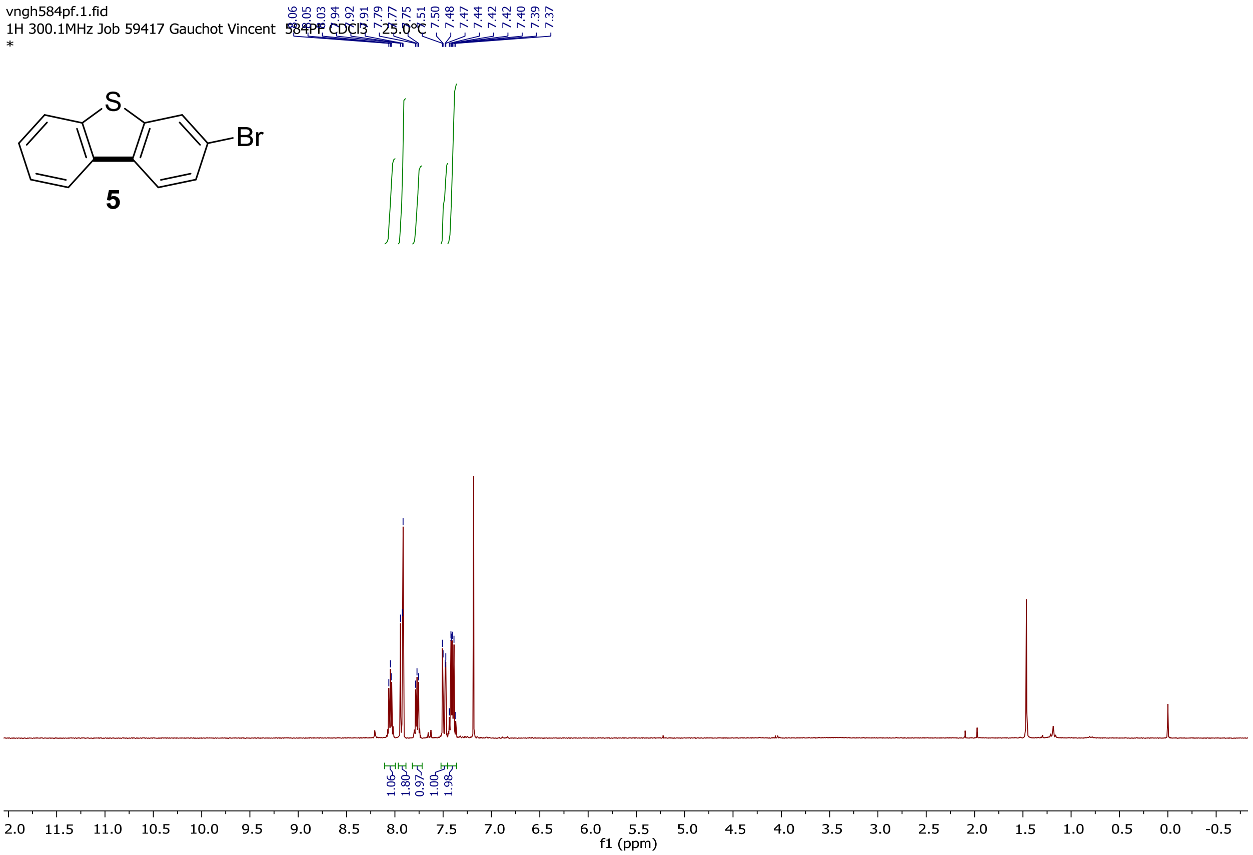
vngh584pf.1.fid
1H 300.1MHz Job 59417 Gauchot Vincent 584PF CDCl3 25.0°C
*
8.06 8.05 8.03 7.94 7.92 7.91 7.79 7.77 7.75 7.51 7.50 7.48 7.47 7.44 7.42 7.42 7.40 7.39 7.37
S
Br
5
1.06 1.80 0.97 1.00 1.98
2.0 11.5 11.0 10.5 10.0 9.5 9.0 8.5 8.0 7.5 7.0 6.5 6.0 5.5 5.0 4.5 4.0 3.5 3.0 2.5 2.0 1.5 1.0 0.5 0.0 -0.5
f1 (ppm)

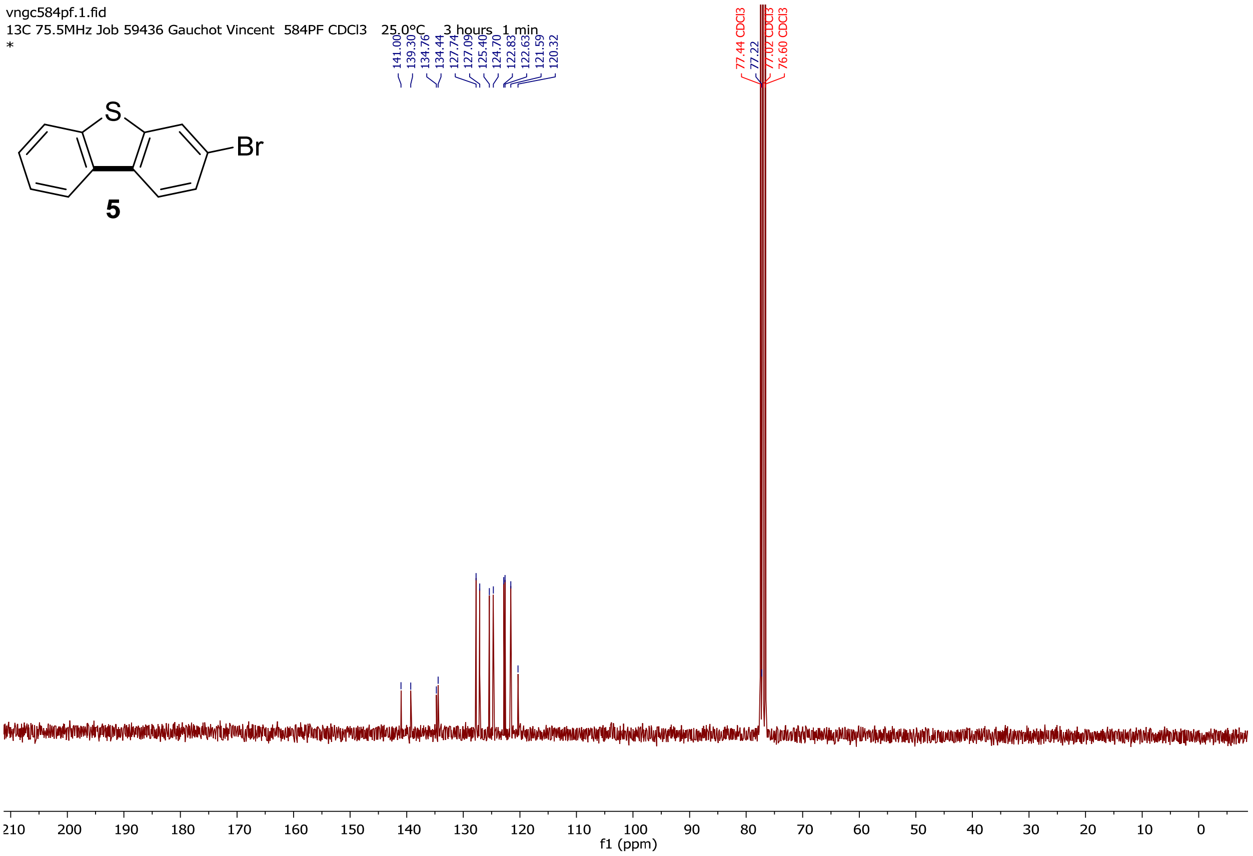
vngc584pf.1.fid
13C 75.5MHz Job 59436 Gauchot Vincent 584PF CDCl3 25.0°C 3 hours 1 min
*
141.00 139.30 134.76 134.44 127.74 127.09 125.40 124.70 122.83 122.63 121.59 120.32
77.44 CDCl3
77.22
77.02 CDCl3
76.60 CDCl3
S
Br
5
210 200 190 180 170 160 150 140 130 120 110 100 90 80 70 60 50 40 30 20 10 0
f1 (ppm)

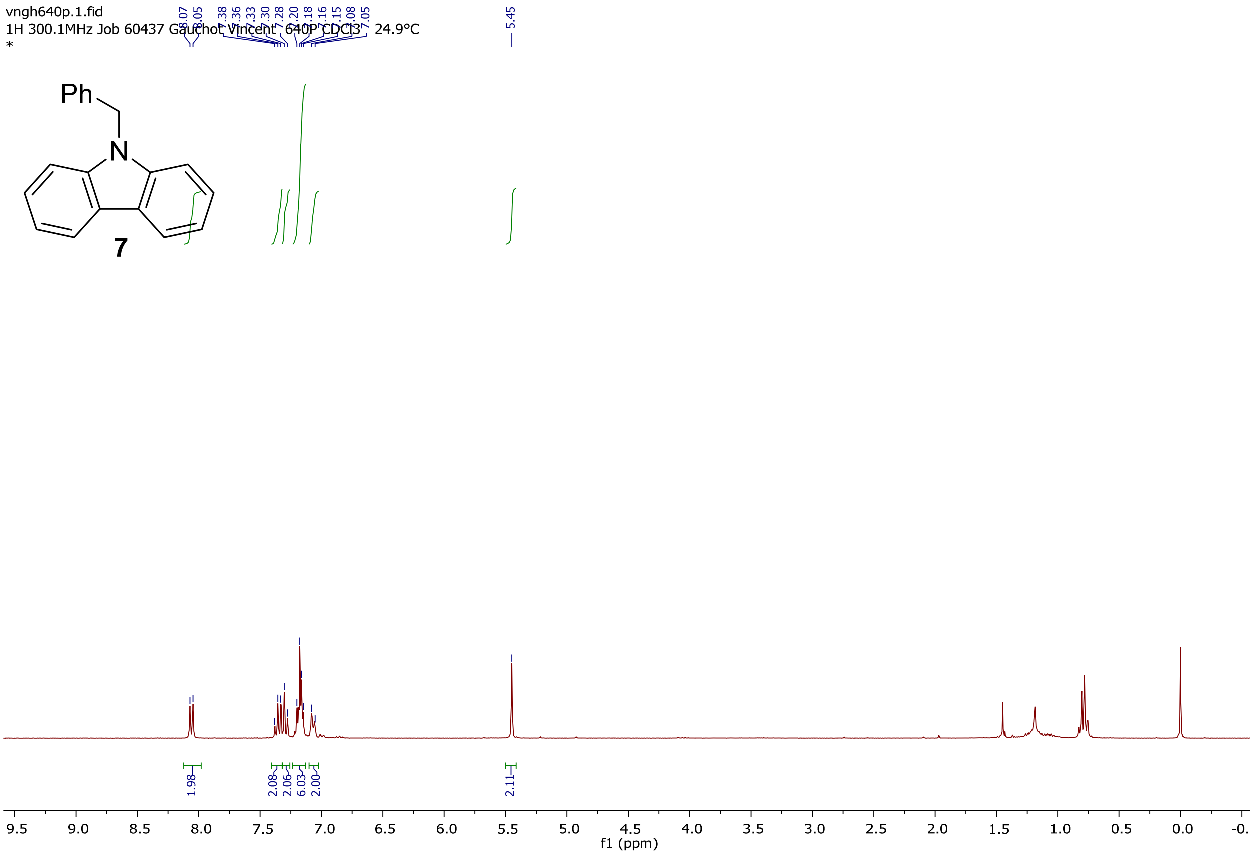

vngh640p.1.fid
1H 300.1MHz Job 60437 Gauchot Vincent 640P CDCl3 24.9°C
*
8.07
8.05
7.38
7.36
7.33
7.30
7.28
7.20
7.18
7.16
7.15
7.08
7.05
5.45
Ph
N
7
1.98
2.08
2.06
6.03
2.00
2.11
9.5
9.0
8.5
8.0
7.5
7.0
6.5
6.0
5.5
5.0
4.5
4.0
3.5
3.0
2.5
2.0
1.5
1.0
0.5
0.0
f1 (ppm)

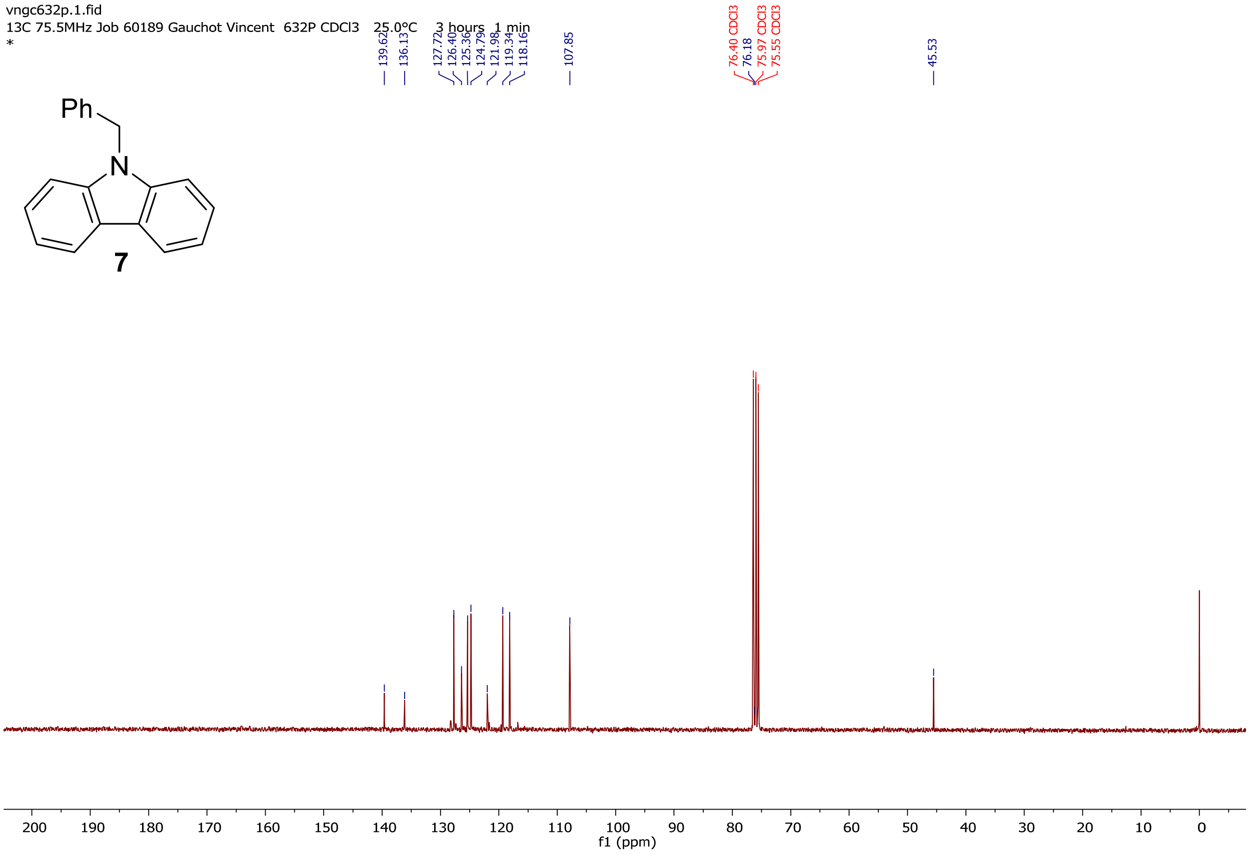

vngc632p.1.fid
13C 75.5MHz Job 60189 Gauchot Vincent 632P CDCl3 25.0°C 3 hours 1 min
*
139.62
136.13
127.72
126.40
125.36
124.79
121.98
119.34
118.16
107.85
76.40 CDCl3
76.18
75.97 CDCl3
75.55 CDCl3
45.53
Ph
N
7
200
190
180
170
160
150
140
130
120
110
100
90
80
70
60
50
40
30
20
10
0
f1 (ppm)

8 - REFERENCES

1 S. Pankajakshan and T. P. Loh, *Chem. - Asian J.*, 2011, **6**, 2291.
2 D. Marcoux and A. B. Charette, *Adv. Synth. & Catal.*, 2008, **350**, 2967.
3 F. Gomes, V. Narbonne, F. Blanchard, G. Maestri and M. Malacria, *Org. Chem. Front.*, 2015, **2**, 464.
4 R. J. Tang, Q. He and L. Yang, *Chem. Commun.*, 2015, **51**, 5925.
5 X. Z. Shu, M. Zhang, Y. He, H. Frei and F. D. Toste, *J. Am. Chem. Soc.*, 2014, **136**, 5844.
6 A. Ikeda, M. Omote, K. Kusumoto, M. Komori, A. Tarui, K. Sato and A. Ando, *Org. Biomol. Chem.*, 2016, **14**, 2127.
7 E. Sperotto, G. P. van Klink, J. G. de Vries and G. van Koten, *J. Org. Chem.*, 2008, **73**, 5625.
8 H. Tian, H. Yang, C. Zhu and H. Fu, *Adv. Synth. & Catal.*, 2015, **357**, 481.
9 F. Jahani, M. Tajbakhsh, S. Khaksar and M. R. Azizi, *Monatsh. Chem.*, 2011, **142**, 1035.
10 M. Findlater, F. Wekesa, N. Phadke, C. Jahier and D. Cordes, *Synthesis*, 2014, **46**, 1046.
11 Q. Liu, Y. Lan, J. Liu, G. Li, Y. D. Wu and A. Lei, *J. Am. Chem. Soc.*, 2009, **131**, 10201.
12 T. Niwa, H. Ochiai, Y. Watanabe and T. Hosoya, *J. Am. Chem. Soc.*, 2015, **137**, 14313.
13 J. Yang, Q. Y. Han, C. L. Zhao, T. Dong, Z. Y. Hou, H. L. Qin and C. P. Zhang, *Org. Biomol. Chem.*, 2016, **14**, 7654.
14 P. Wipf and J.-K. Jung, *J. Org Chem.*, 2000, **65**, 6319.
15 B. Lin, Z. Liu, M. Liu, C. Pan, J. Ding, H. Wu and J. Cheng, *Catal. Commun.*, 2007, **8**, 2150.
16 N. Rameau, S. Cadot, A. Paquet, C. Pinel and L. Djakovitch, *Topics in Catalysis*, 2014, **57**, 1430.
17 J. delPozo, J. A. Casares and P. Espinet, *Chem. - Eur. J.*, 2016, **22**, 4274.
18 B. Sreedhar, D. Yada and P. S. Reddy, *Adv. Synth. & Catal.*, 2011, **353**, 2823.
19 K. M. Liu, J. Wei and X. F. Duan, *Chem. Commun.*, 2015, **51**, 4655.
20 S. M. Wang, X. Y. Wang, H. L. Qin and C. P. Zhang, *Chem. - Eur. J.*, 2016, **22**, 6542.
21 Y. Zhou and J. G. Verkade, *Adv. Synth. & Catal.*, 2010, **352**, 616.
22 V. Michelet, P. Toullec, A. Pradal and P. Faudot dit Bel, *Synthesis*, 2012, **44**, 2463.
23 L. M. Anderson, A. R. Butler and A. S. McIntosh, *J. Chem. Soc., Perkin Trans. 2*, 1987, 1239.
24 G. N. Kurilo, N. I. Rostova, A. A. Cherkasova and A. N. Grinev, *Khimiya Geterotsiklicheskikh Soedinenii*, 1977, **3**, 353.
